# Particle-environment interactions in arbitrary dimensions: A unifying analytic framework to model diffusion with inert spatial heterogeneities

**DOI:** 10.1103/PhysRevResearch.5.043281

**Published:** 2023-12-22

**Authors:** Seeralan Sarvaharman, Luca Giuggioli

**Affiliations:** School of Engineering Mathematics and Technology, University of Bristol, Bristol BS8 1TW, United Kingdom

## Abstract

Inert interactions between randomly moving entities and spatial disorder play a crucial role in quantifying the diffusive properties of a system, with examples ranging from molecules advancing along dendritic spines to antipredator displacements of animals due to sparse vegetation. Despite the ubiquity of such phenomena, a general framework to model the movement explicitly in the presence of spatial heterogeneities is missing. Here, we tackle this challenge and develop an analytic theory to model inert particle-environment interactions in domains of arbitrary shape and dimensions. We use a discrete space formulation, which allows us to model the interactions between an agent and the environment as perturbed dynamics between lattice sites. Interactions from spatial disorder, such as impenetrable and permeable obstacles or regions of increased or decreased diffusivity, as well as many others, can be modelled using our framework. We provide exact expressions for the generating function of the occupation probability of the diffusing particle and related transport quantities such as first-passage, return, and exit probabilities and their respective means. We uncover a surprising property, the disorder indifference phenomenon of the mean first-passage time in the presence of a permeable barrier in quasi-1D systems. We demonstrate the widespread applicability of our formalism by considering three examples that span across scales and disciplines. (1) We explore an enhancement strategy of transdermal drug delivery. (2) We represent the movement decisions of an animal undergoing thigomotaxis, the tendency to remain at the peripheries of its enclosure, using a spatially disordered environment. (3) We illustrate the use of spatial heterogeneities to model inert interactions between particles by modeling the search for a promoter region on the DNA by transcription factors during gene transcription.

## I Introduction

Local interactions between mobile agents or particles and their environmental features plays a crucial role in the dynamics of many systems across disciplines and scales [1–5]. When such environmental features are inert heterogeneities, the local interactions only affect the movement dynamics of the agents. A wide array of spatial heterogeneities can be classed as inert, e.g., impenetrable or permeable barriers, areas of reduced or increased mobility, lattice defects such as disclinations, and traps that are reversible.

In some instances, the presence of such heterogeneities is by design, e.g., in manufacture engineering where materials are constructed to have specified diffusive characteristics [6,7]. In other scenarios, spatial heterogeneities occur naturally. In ecology, animals alter their foraging behavior due to variations in vegetation cover [8,9]. In molecular biology, particles undergo fence hindered motion in the lipid bilayer membranes of eukaryotes [10,11], and slow down dramatically when moving within the cell cytoplasm due to exclusion processes [12]. While the relationship between mobility and spatial disorder in these and other systems has always been a focus of scientific studies, it is the highly resolved nature of modern observations that has made apparent the need for a general framework to model inert particle-environment interactions.

Investigations on movement dynamics in spatially disordered systems date back as early as the 50’s [13–17]. Despite such a long history most analyses lack a rigorous quantitative description of the “microscopy” of the interaction events between the particle and the environment. In the past, transport in highly disordered media has been studied approximately, linking the Hausdorff–Besicovitch dimension of fractal structures to a diffusion constant via scaling arguments [18]. Other approaches have kept the geometry nonfractal utilizing random walks on regular lattices, the so-called random walk in random environments model [19–25]. These studies have been instrumental for bringing to light universal concepts such as weak ergodicity breaking and power-law waiting times [26,27] as properties of disordered environments. They also often pertain to 1D domains [28], and have used techniques such as the effective medium approximation to find statistical properties of the movement dynamics. It is precisely the absence of explicit spatiotemporal representation of higher-dimensional particle dynamics, that has hampered the widespread applicability of these various models to current high-fidelity observations.

More recent theoretical applications to movement in disordered environments have focused on the diffusive dynamics in the cell (e.g., see reviews in Refs. [29–31]). Many attempts in this area are macroscopic and tackle particle dynamics without representing the local interactions. Some efforts, giving importance to the very slow dynamics that emerge from overcrowding effects, have modelled particle movement via fractional diffusion [32]. The relative size of accessible versus inaccessible regions has been accounted for using diffusion on percolation clusters and has highlighted the difference between compact versus noncompact exploration of space [33]. Such theoretical efforts have provided valuable insights such as the emergence of subdiffusion [34] or non-Gaussian yet Brownian motion from quenched disorder [35].

Other investigations have put emphasis on the spatiotemporal dynamics of the environment and, inspired by recent experiments [36–38], have developed the so-called diffusing-diffusivity models, where the diffusion strength of the medium itself is a random variable [39–42] or more recently a correlated random variable [43]. Such models have also been the subject of theoretical investigations [44,45]. These approaches have brought important insights and have broadened the tools and techniques with which to study disordered systems. However, they too lack the mechanistic connection between the environmental heterogeneities and the moving particle [32]. With the advent of new experimental techniques such as super-resolution microscopy and single-particle tracking [46], the need for an explicit consideration of particle-environment interactions has also emerged in microbiology [47,48].

The challenge in fulfilling this need stems from the symmetry breaking role that disorder plays on the underlying diffusive dynamics. In most instances describing explicitly multiple heterogeneities is an unwieldy boundary value problem. The vast majority of theoretical studies have in fact been limited to highly symmetric scenarios, e.g., spherically symmetric domains with concentric layers of different diffusivity [49–52] and an array of periodically placed semipermeable barriers in 1D [53–57].

To bypass this challenge, and to avoid the use of computationally prohibitive stochastic simulations, we propose a unifying analytic framework to model interactions between diffusing agents and spatial disorder. We do so by developing a random walk theory where interactions with heterogeneities are represented as a perturbation of the transition probabilities of a homogeneous lattice. By extending the so-called defect technique [58–60], we are able to model explicitly any inert particle-environment interactions in arbitrary dimensions, e.g., the passage through porous or permeable barriers, the movement within regions of altered diffusivity, which we call sticky or slippery sites as well as shortcut jumps to far away locations.

The theory allows us to derive mathematical expressions for the random walker occupation probability, the so-called propagator. The generating function of these propagators are exact and obtained in terms of the occupation probability in the absence of spatial heterogeneities, thereby making our framework modular in its application. Multiple derived quantities, such as first-passage, return, and exit probabilities, which in the past were obtained either numerically or known only in asymptotic limits [61], can now be readily computed via the evaluation of certain matrix determinants.

Given the generality of our framework, we have opted to provide three examples of application. The first deals with an extracellular process, namely the potential optimization of transdermal drug delivery [62,63]. The second example is the modeling of thigmotaxis, the tendency of insects and other animals to remain preferentially close to physical boundaries whilst moving [64,65]. The third application concerns with the search dynamics in a two-particle coalescing process that is of relevance to early stages of gene transcription [66,67].

The remainder of the paper is organized as follows. In Sec. II we introduce the general mathematical formalism via a lattice random-walk Master equation, and show how we represent different kinds of heterogeneities. In Sec. III we solve the Master equation and find the exact propagator. Section IV deals with first-passage statistics and their associated mean, i.e., mean first-passage, mean exit, and mean return times. We discuss the computational advantage of evaluating firstpassage statistics over existing methods in Sec. V. The latter half of the paper, Secs. VI, VII, and VIII, are devoted to the three applications mentioned previously, which are transdermal drug delivery, thigmotaxis, and gene transcription. Lastly, conclusions and future applications form Sec. IX.

## II Movement in Heterogeneous Environments

We start by defining the dynamics of a Markov lattice random walk on a *d*-dimensional lattice via

(1) $$\varphi (n,t+1)=\underset{m}{\sum }{\underline{A}}_{n,m}\varphi (m,t),$$

where ***n*** is a *d*-dimensional vector and ***A***_***n***,***m***_ is the transition probability from site ***m*** to site ***n*** such that Σ_***m***_ ***A***_***m***,***n***_ = 1 for any site ***n*** on the lattice, i.e., with *d* = 1, ***A*** is a probability conserving transition matrix, and when *d >* 1, ***A*** is actually a tensor. For convenience in inverting generating functions, as compared to Laplace inversion, we use a discrete time formulation with the variable *t*. Changes to a continuous time description is straightforward [68], but is omitted here. We refer to this equation as the homogeneous Master equation and its solution, given a localized initial condition, as the *homogeneous propagator*. The underlying lattice is referred to as the homogeneous lattice whose size can be finite or infinite.

**Fig. 1:**
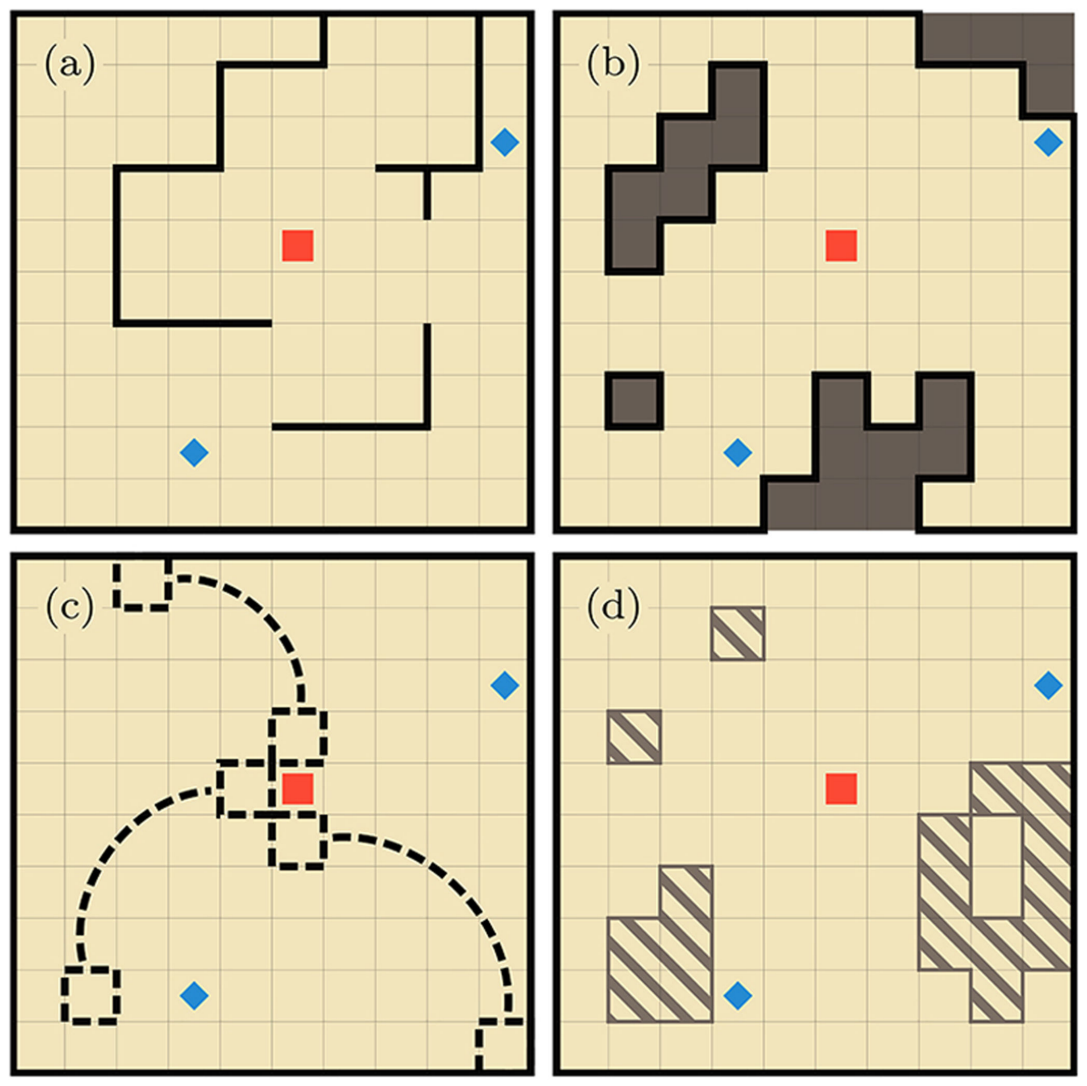
Examples of the spatial heterogeneities within a square lattice of width 10 with reflecting boundaries. Panel (a) depicts open partitions with the solid black lines indicating impenetrable barriers. When these barriers enclose a region, some space becomes inaccessible indicated by the sites colored dark grey in panel (b). Panel (c) shows a lattice where three pairs of non-neighboring sites have a long-range connection, i.e., transitions from a dashed site include the nearest-neighbors as well as the site connected via the dashed line. Panel (d) is an example of where the diffusivity of the striped sites is smaller than the regular (nonstriped) sites. The central site flagged by a red square and the two sites flagged by a blue diamond are, respectively, the initial condition and the absorbing targets for use in later sections.

Since spatially heterogeneous dynamics are defined relative to the homogeneous system, we define heterogeneities as locations or *defects* where the dynamics are different from the corresponding ones on the homogeneous lattice. Examples of heterogeneities are depicted in Fig. 1.

**Fig. 2:**
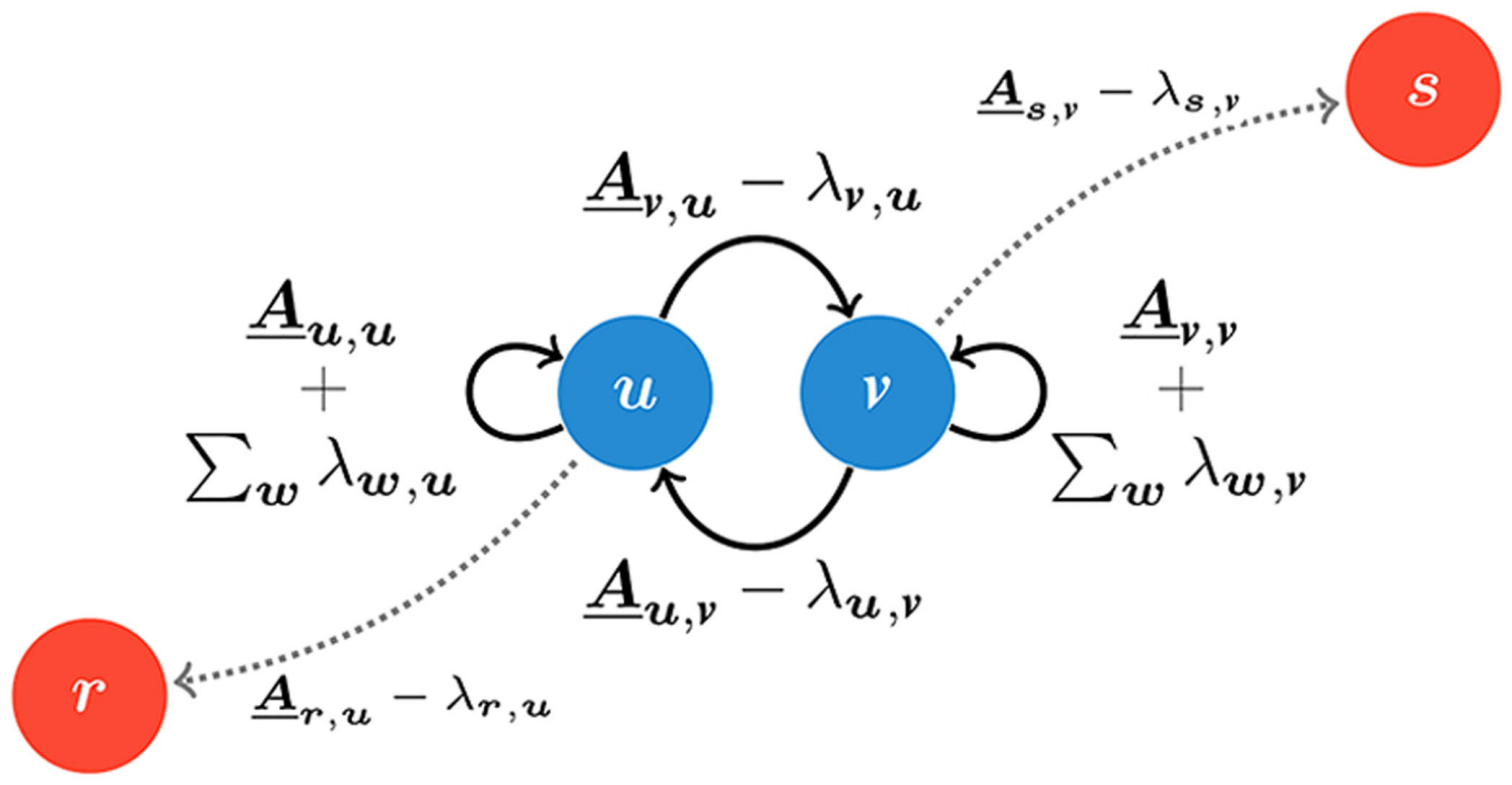
A schematic representation of the transition probabilities after the introduction of spatial heterogeneity or disorder. The probability of hopping from site ***u*** to ***v*** is given by ***A***_***v***,***u***_. When λ_***v***,***u***_ is positive, the probability of jumping from ***u*** to ***v*** decreases, while the probability of staying put increases. When λ_***v***,***u***_ is negative, the opposite effect occurs with a decrease in the probability of staying, while increasing the jump probability from ***u*** to ***v***. The parameter λ_***u***,***v***_ affects the transition probability from ***v*** to ***u*** and the probability of remaining at ***v*** in an equivalent manner.

The heterogeneities displayed in Fig. 1 emerge from the modification of the outgoing transitions from one or more sites, hence we refer to these altered transitions as heterogeneous connections. For example, given a partially reflecting barrier in between two neighboring sites, the jump probability from either of the two sites to the other is reduced, while the probability of staying put at either of the sites is increased. Conversely, by connecting together two non-neighboring sites, we may wish to reduce the probability of staying put at a given site, whilst adding the possibility of hopping to the site further away. One can represent conveniently these or any other heterogeneity through a modification of the transitions as depicted in Fig. 2. Formally, the outgoing connections of the sites ***u*** and ***v*** are adjusted by introducing the parameters λ_***v***,***u***_ and λ_***u***,***v***_ to create two heterogeneous connections. Although we choose to modify transitions in both direction, i.e., from ***u*** to ***v*** and ***v*** to ***u***, this does not have to be the case. Modifications of only outgoing connections are also permitted, e.g., see the dashed arrows connecting the additional sites ***r*** and ***s***.

The construction implicitly conserves probability, which can be evinced by picking a defect, e.g., ***u***, and summing over all of the outgoing probabilities. The changes induced by the λ parameters cancel each other out leaving Σ_***w***_ ***A***_***w***,***u***_, with ***w*** representing all the neighbors of ***u***, equal to the homogeneous outgoing probability. To ensure positive probability for a given heterogeneous site ***u***, we have the conditions

(2) $$\lambda_{\text{w},\text{u}}⩽{\underline{A}}_{w,\text{u}},$$

for all ***w*** with a heterogeneous connection in the direction ***u*** to ***w***, and

(3) $$0⩽{\underline{\text{A}}}_{\text{u},\text{u}}+\underset{\text{w}}{\sum }\lambda_{\text{w},\text{u}},$$

which enforces upper and lower bounds on the λ parameters although each one of them can be positive or negative. This formulation allows one to perturb arbitrarily the homogeneous lattice creating any type of probability conserving particle-environment interactions.

### A Quantitative representation of heterogeneities

To understand the practicality of the formalism, we focus on the three specific types of heterogeneities in Fig. 1, namely, barriers [Figs. 1(a) and 1(b)], long-range connections [Fig. 1(c)], and sticky sites [Fig. 1(d)]. In the following subsection, we present convenient parametrization for the constant λ’s to construct such heterogeneities.

#### 1 Barriers and anti-barriers

**Fig. 3:**
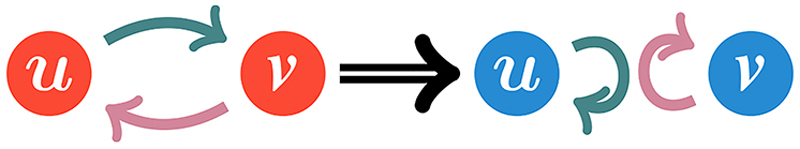
Example of a reflecting barrier between ***u*** and ***v*** generated by modifying the transition probabilities (from the left to the right of the schematic). The modified transitions are indicated by colored arrows. The modification in this case results in an impenetrable barrier between ***u*** and ***v***, with *α*_***v***_ = *α*_***u***_ = 1.

With ***u*** and ***v*** two neighboring sites, we construct a partially reflecting barrier by having λ_***v***,***u***_ = *α*_***v***_ ***A***_***v***,***u***_ and λ_***u***,***v***_ = *α*_***u***_ ***A***_***u***,***v***_ where *α*_***v***_, *α*_***u***_ ∈ [0, 1] is a measure of the reflectivity of the barrier. When *α*_***v***_, *α*_***u***_ = 1 we have an impenetrable barrier (shown in Fig. 3), while with *α*_***v***_, *α*_***u***_ = 0 we regain the homogeneous transition. Notice that the barrier does not need to be symmetric, i.e., *α*_***u***_ ≠ *α*_***v***_, with the extreme scenario being a barrier with λ_***v***,***u***_ = ***A***_***v***,***u***_ and λ_***u***,***v***_ = 0 yields a one-way barrier or gate. In such a case the movement from ***v*** to ***u*** is allowed but from ***u*** to ***v*** is not.

It is also possible to have dynamics opposite to the partially reflecting barrier. In this case, again with ***u*** and ***v*** two neighboring sites, one has λ_***v***,***u***_ = − *β*_***v***_ ***A***_***u***,***u***_ and λ_***u***,***v***_ = − *β*_***u***_ ***A***_***v***,***v***_ where *β*_***v***_, *β*_***u***_ ∈ [0, 1]. As the probability of jumping to the neighbors increases whilst the probability of staying put decreases, we have chosen the name antibarrier for this type of heterogeneity.

#### 2 Long-range connection

**Fig. 4:**
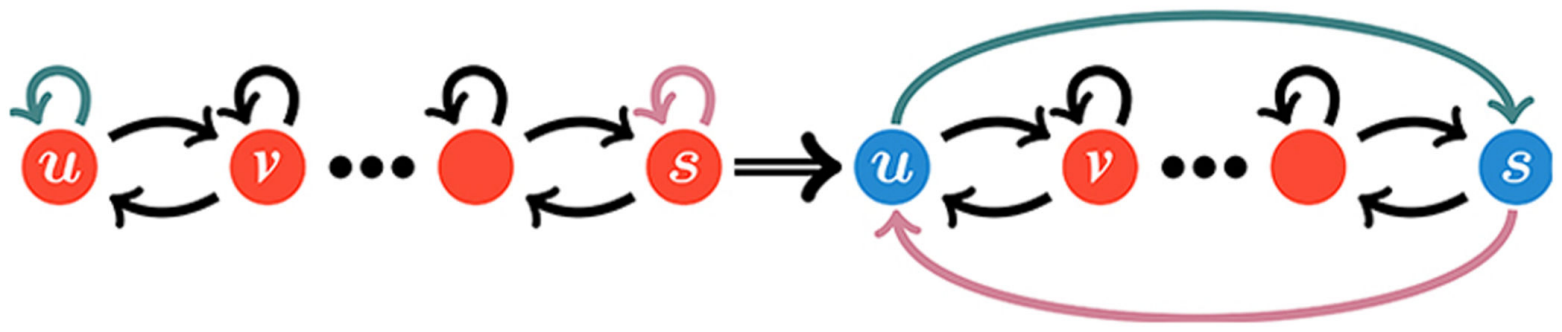
Example of a long-range connection obtained by rewiring the lazy probability of ***u*** and ***v*** to create a long-range connection between them (from left to right). In this case *β*_***u***_ = *β*_***s***_ = 1.

When adding an outgoing long-range connection one has to draw the probability from one or more of the existing transitions. Let us consider a site ***u*** and a non-neighboring destination site ***s***, where ***A***_***s***,***u***_ = ***A***_***u***,***s***_ = 0. One way of introducing the outgoing long-range connection is to draw upon the lazy (also called sojourn) probability using λ_***s***,***u***_ = − *β*_***s***_ ***A***_***u***,***u***_ and λ_***u***,***s***_ = − *β*_***u***_ ***A***_***s***,***s***_, where *β*_***u***_, *β*_***s***_ ∈ [0, 1] is the proportion of the lazy probability added to the long-range connection, see Fig. 4 for a pictorial representation.

Note this is not the only way; one can also rewire an existing connection from a neighbor to the non-neighbor. In such a case, with ***v*** a neighbor of ***u***, we let λ_***v***,***u***_ = ***A***_***v***,***u***_ and λ_***s***,***u***_ = − ***A***_***v***,***u***_. The former removes the possibility of jumping from ***u*** to the neighbor ***v***, whilst the latter adds the possibility of hopping from ***u*** to the non-neighbor ***s***.

#### 3 Sticky and slippery sites

**Fig. 5:**
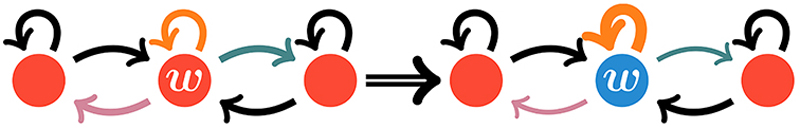
Example of a sticky site on a 1D lattice generated by reducing all of the outgoing probability to the neighbors as shown by the thinner arrows, whilst increasing the staying probability of ***w*** as shown by the thicker self loop (from left to right).

Adding a partially reflecting barrier between two neighboring sites naturally increases the probability of staying. By harnessing this property one can use multiple one-way partially reflecting barriers between a site ***w*** and all of its *k* nearest neighbors, ***r***_1_,…, ***r***_*k*_, giving $\lambda_{r_{i},w}=\alpha {\underline{A}}_{r_{i},w}$ and $\lambda_{w,r_{i}}=0$ with *α* ∈ [0, 1] for all *i* = 1,…, *k*. The result is a sticky site ***w***, where the probability of staying is increased, whilst the probability of jumping to any of its neighbors is decreased. The introduction of *α* is used to control and distribute the stickiness equally across the neighbors in a convenient manner. See Fig. 5 for a pictorial representation on a 1D lattice.

Conversely, keeping $\lambda_{w,r_{i}}=0$ and letting $\lambda_{r_{i},w}=-\frac{\beta }{k}{\underline{A}}_{w,w}$ with *β* ∈ [0, 1] for all *i* = 1,…, *k* yields a slippery site with opposite dynamics. As for the sticky site, the introduction of *β* is used to control the slippery quality of the site ***w*** equally among its neighbors. Note that we have chosen to divide *β* by *k* so that Eq. (3) is automatically satisfied.

## III Heterogeneous Propagator

We consider an arbitrary collection of heterogeneous connections given by a set of *M* paired defective sites or defects, *S* {{***u***_1_, ***v***_1_},…, {***u***_*M*_, ***v***_*M*_}}. We use ***u***_*i*_ and ***v***_*i*_ with subscripts to indicate the two members of the *i*th pair, while ***u*** and ***v*** without subscripts refers to a generic pair in *S*. The pairs are unique, i.e., {***u***_*i*_, ***v***_*i*_} ≠ {***u***_*j*_, ***v***_*j*_} for any *i* ≠ *j*; however, a site can be part of multiple pairs. For example, the set of pairs, which represents the schematic depicted in Fig. 2 is *S* = {{***u, v***}, {***u, r***}, {***v, s***}}, with the sites ***u*** and ***v*** being part of two pairs while the sites ***r*** and ***s*** being part of only one pair each. The evolution of the occupation probability is given by the Master equation

(4) $$\begin{array}{c} \text{Φ}(n,t+1)=\underset{\text{m}}{\sum }{\underline{A}}_{\text{n},\text{m}}\text{Φ}(m,t)+\sum_{k=1}^{M}\left(\delta_{n,u_{k}}-\delta_{n,v_{k}}\right)\\ \times \left[\lambda_{v_{k},u_{k}}\text{Φ}\left(u_{k},t\right)-\lambda_{u_{k},{\text{v}}_{k}}\text{Φ}\left(v_{k},t\right)\right], \end{array}$$

where the second summation is over all pairs of heterogeneous connections. When all λ parameters are set equal to zero, Eq. (4) reduces to Eq. (1) and the occupation probability on the heterogeneous lattice Φ(***n***, *t*) reduces to that of the homogeneous lattice *φ*(***n***, *t*).

One can find the generating function (*z*-domain) solution of Eq. (4) by generalizing the so-called defect technique to obtain

(5) $${\tilde{\Phi }}_{n_{0}}(n,z)={\tilde{\varphi }}_{n_{0}}(n,z)-1+\frac{|\underline{H}\left(n,n_{0}\right)|}{\left|\underline{H}\right|},$$

where $\tilde{f}(z)={\text{Σ}}_{t=0}^{\infty }f(t)z^{t}$ is the generating function of the time-dependent function $f(t),{\tilde{\varphi }}_{n_{0}}(n,z)$ is the propagator generating function of Eq. (1), while |***H***| and |***H*** (***n, n***_0_)| are determinants with

(6) $${\underline{\text{H}}}_{i,j}=\lambda_{{\text{v}}_{i},{\text{u}}_{i}}{\tilde{\varphi }}_{\langle {\text{u}}_{j}-{\text{v}}_{j}\rangle }\left({\text{u}}_{i},z\right)-\lambda_{{\text{u}}_{i},{\text{v}}_{i}}{\tilde{\varphi }}_{\langle {\text{u}}_{j}-{\text{v}}_{j}\rangle }\left(v_{i},\text{z}\right)-{\text{z}}^{-1}\delta_{i,j},$$

(7) $$\begin{array}{c} \underline{H}{\left(n,n_{0}\right)}_{i,j}={\underline{H}}_{i,j}-{\tilde{\varphi }}_{\langle u_{j}-v_{j}\rangle }(n,z)\\ \times \left[\lambda_{v_{i},u_{i}}{\tilde{\varphi }}_{n_{0}}\left(u_{i},z\right)-\lambda_{u_{i},v_{i}}{\tilde{\varphi }}_{n_{0}}\left(v_{i},z\right)\right]. \end{array}$$

In Eqs. (6) and (7) we have used the notation ${\tilde{\varphi }}_{\langle u-v\rangle }(n,z)={\tilde{\varphi }}_{u}(n,z)-{\tilde{\varphi }}_{v}(n,z)$. From here onwards we refer to ${\tilde{\varphi }}_{n_{0}}(n,z)$ as the homogeneous propagator, which are known in closed form in finite domains and in a variety of scenarios [68–70], while ${\tilde{\Phi }}_{n_{0}}(n,z)$ is referred to as the heterogeneous propagator. When *t* = 0, that is *z* = 0, we have ${\tilde{\varphi }}_{n_{0}}(n,0)=\delta_{n_{0},n},$ while |***H*** (***n, n***_0_) | */* | ***H*** | _=_ 1 and we recover the appropriate initial condition, ${\tilde{\Phi }}_{n_{0}}(n,0)=\delta_{n_{0},n}$.

In general, the size of matrices ***H*** and ***H*** (***n, n***_0_) depend on the number of paired defects *M*. A *d*-dimensional walk with one sticky (or slippery) site requires two paired defects for each of the *d* dimensions. However, in this case, one can make a simplification and reduce the size of the matrices by a factor of 2*d*. Those simplified matrices, as well as the derivation of the solution and details of efficient evaluation of the solution, can be found in Appendices D and H.

**Fig. 6:**
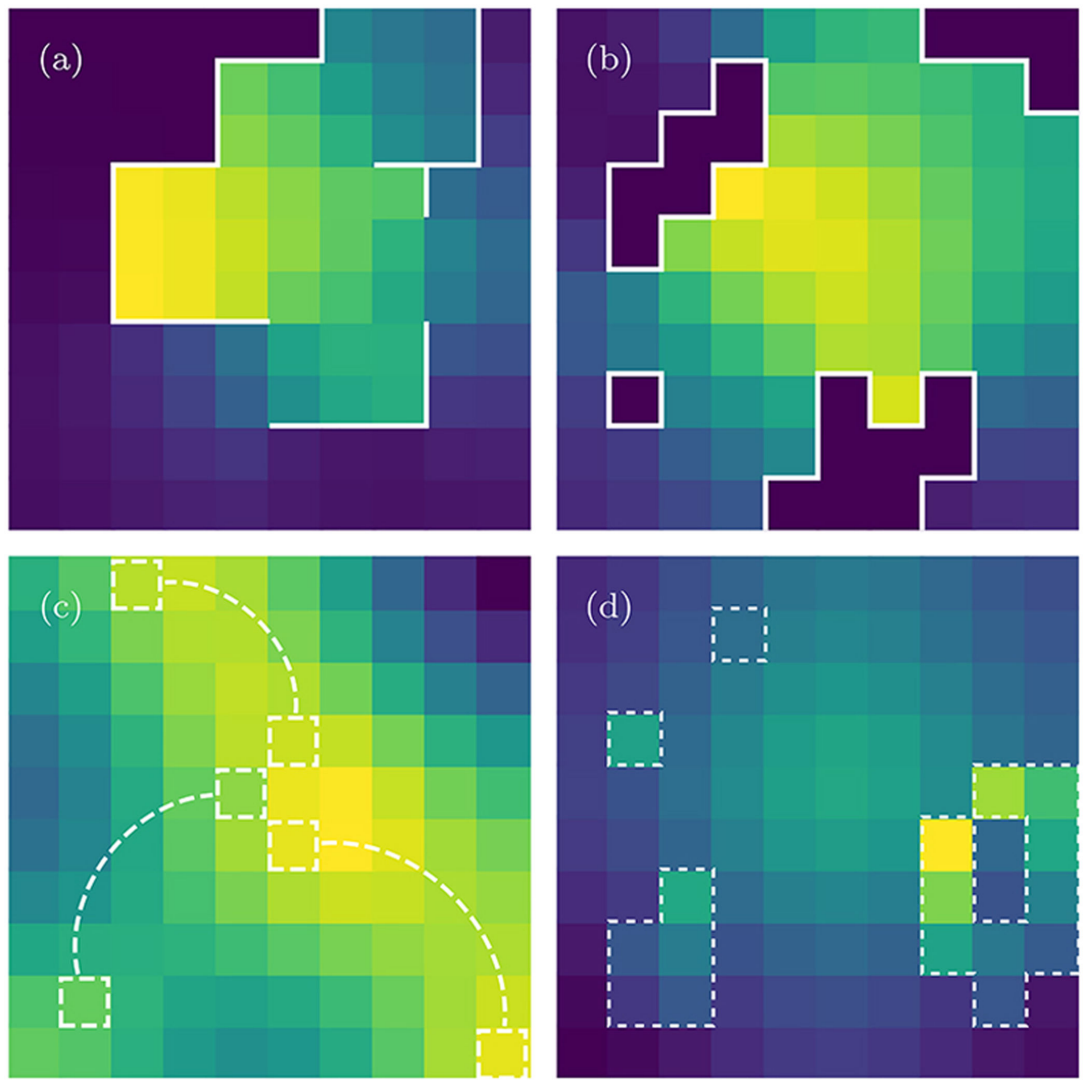
A snapshot of ${\text{Φ}}_{n_{0}}(n,t)$ at time *t* = 100 obtained from Eq. (5) with standard numerical methods [71,72]. Propagator with different configurations of defects, corresponding with Fig. 1, at *t* = 100. For all panels, the homogeneous propagator, ${\tilde{\varphi }}_{n_{0}}(n,z)$ is the 2D propagator with reflecting boundaries given in Eq. (23) of Ref. [68] (see also Appendix H). The parameters used are: a domain of size ***N*** = (10, 10), a localized initial condition with ***n***_0_ = (6, 6). We a use diffusion parameter of value ***q*** = (0.2, 0.2), which gives the following transition probabilities: in the bulk of the homogeneous lattice the probability of jumping to one of the four neighbors is ***A***_***r***,***s***_ = 0.05 (with ***r*** ≠ ***s***), while the probability of staying at the same site is ***A***_***r***,***r***_ = 0.8. The reflecting barriers and other heterogeneities are super imposed on top of the probability. For panels (a) and (b), λ_***v***,***u***_ = ***A***_***v***,***u***_ and λ_***u***,***v***_ = ***A***_***v***,***u***_ (for all {***u, v***}∈ *S*) yielding perfectly reflecting barriers. For panel (c) $\lambda_{\text{v},\text{u}}=-\frac{1}{2}{\underline{A}}_{\text{u},\text{u}}$, and $\lambda_{\text{u},\text{v}}=-\frac{1}{2}{\underline{A}}_{\text{v},\text{v}}$. With this perturbation, when on one of the defective sites, the probability of staying is reduced to ***A***_***u***,***u***_ = ***A***_***v***,***v***_ = 0.4, while the probability of jumping to the non-neighbor is increased (from zero) to ***A***_***u***,***v***_ = ***A***_***v***,***u***_ = 0.4. Lastly, for panel (d), for each of the sticky sites ***w*** with *k* neighbors ***r***_1_,…, ***r***_*k*_ we use $\lambda_{r_{i},w}=\frac{1}{4}{\underline{A}}_{r_{i},w}$ (see Sec. II A 3). For convenience we have omitted color bars for each panel as we are interested only in the relative differences of the occupation probability.

In Fig. 6 we plot a snapshot of ${\tilde{\Phi }}_{n_{0}}(n,t)$ for the heterogeneities depicted in each of the panels of Fig. 1. We use this figure to demonstrate the qualitative features in the dynamics, therefore we have omitted the color bars and have chosen a small domain where such features are more apparent. In panel (a) the lattice is partitioned by impenetrable barriers represented by the solid white lines. Here, one can observe the lowest probabilities in the top-left corner since the walker has not had the time to travel around the barriers. Panel (b) contains areas enclosed by impenetrable barriers, with occupation probabilities that are always zero. The long-range connection shown in panel (c) has enabled the walker to spread further than in other panels. Small peaks in the probability can be observed away from the initial condition, in the top-left, bottom-left and bottom right corners. In panel (d) the sticky regions tend to show a higher occupation probability compared to the homogeneous sites.

Note that we have not placed any restriction on whether (the homogeneous propagator) ${\tilde{\varphi }}_{n_{0}}(n,z)$ conserves probability or note. When there are fully or partially absorbing sites, one may proceed in two ways. (i) In the first approach one account for the absorbing dynamics by finding the propagator ${\tilde{\varphi }}_{n_{0}}(n,z)$ that satisfies appropriate boundary conditions, before adding inert disorder via Eq. (5). (ii) In the second approach one would take ${\tilde{\varphi }}_{n_{0}}(n,z)$ without any absorbing locations, construct ${\tilde{\varphi }}_{n_{0}}(n,z)$ and then add the absorbing sites using the standard defect technique in the presence of absorbing sites [60]. While the choice makes no impact on the final dynamics, depending on the situation one procedure may be more convenient than the other.

## IV First-Passage Processes

An important quantity derived from the propagators is the first-passage statistics to a set of targets. It is relevant to stochastic search in movement ecology [73], swarm robotics [74] and many other areas [75].

The first-passage probability $F_{n_{0}}(n,t)$ that is the probability to reach ***n*** for the first time at *t* having started at ***n***_0_, is related to the propagator, ${\text{Φ}}_{n_{0}}(n,t)$ by the renewal equation. When ***n* ≠ *n***_0_, the well-known relation in *z* domain is given by ${\tilde{F}}_{n_{0}}(n,z)={\tilde{\Phi }}_{n_{0}}(n,z)/{\tilde{\Phi }}_{n}(n,z)$. Having the first-passage probability in closed form allows one to substitute the heterogeneous first-passage probability 𝔽_*n*_0__(*n*, *t*) [or ${\tilde{F}}_{n_{0}}(n,z)$] in place of the homogeneous counterpart in other established contexts where homogeneous space was previously assumed.

One such context is a first passage in the presence of multiple targets, where one is interested in the probability of being absorbed at any of the targets. We use recent findings [68] to determine the dynamics of a lattice walker to reach either of two sites, ***n***_1_, and ***n***_2_, for the first time at *t* in the presence of spatial heterogeneities, given by $F_{n_{0}}\left(n_{1},n_{2},t\right)$. The generating function of this probability, given by ${\tilde{F}}_{n_{0}}\left(n_{1},n_{2},z\right)=\{{\tilde{F}}_{n_{0}}\left(n_{1},z\right)\left[1-{\tilde{F}}_{n_{1}}\left(n_{2},z\right)\right]+{\tilde{F}}_{n_{0}}\left(n_{2},z\right)\left[1-{\tilde{F}}_{n_{2}}\left(n_{1},z\right)\right]\}\times {[1-{\tilde{F}}_{n_{1}}\left(n_{2},z\right){\tilde{F}}_{n_{1}}\left(n_{2},z\right)]}^{-1}$ {taken from Eq. (38) in Ref. [68]}, is expressed in terms of the first-passage probabilities to single targets.

**Fig. 7:**
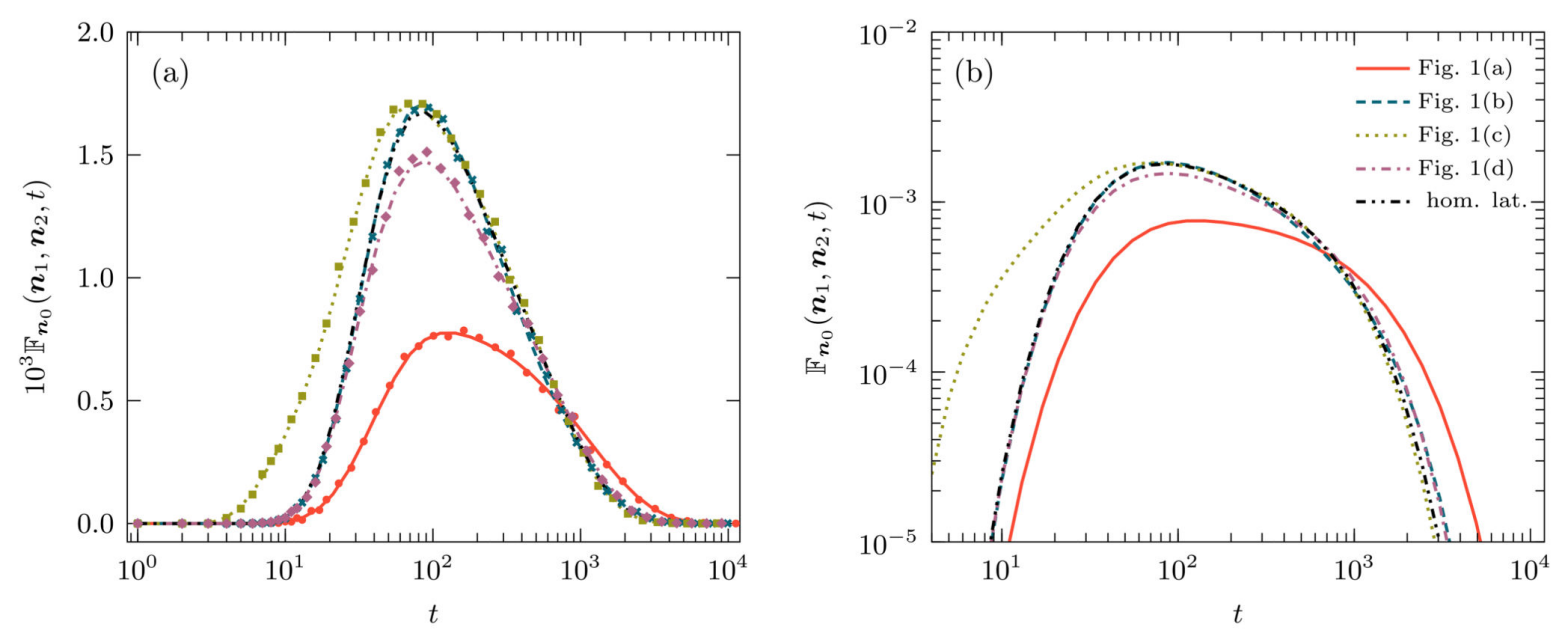
Time-dependent first-passage probability to either of two targets in the presence of heterogeneities. The location of the targets ***n***_1_ = (4, 2) and ***n***_2_ = (10, 7) in relation to the initial condition ***n***_0_ = (6, 6) and visualization of the heterogeneities present can be seen in the schematic diagram in Fig. 1. We use a homogeneous propagator ${\tilde{\varphi }}_{n_{0}}(n,z)$ with a reflecting domain of size ***N*** = (10, 10) and a diffusion parameter of value ***q*** = (0.2, 0.2). The explicit form of ${\tilde{\varphi }}_{n_{0}}(n,z)$ is given by Eq. (23) of Ref. [68]. The lines are obtained through numerical inversion of the generating function of the first-passage probability to either of two targets (see text), while the corresponding marks—shown only in panel (a)—are obtained through 1.5 × 10^6^ stochastic simulations.

In Fig. 7 plot the time-dependent probability for the heterogeneity examples shown in Fig. 1. The first nonzero probability corresponds to the length of the shortest path to either of the targets, which in the absence of heterogeneities and for the examples in Figs. 1(a), 1(b), and 1(d) is 6, whereas for the Fig. 1(c) one can reach the target ***n***_1_ from ***n***_0_ in 4 steps as a result of the nearest long-range connection. It is clearly visible in the earlier rise of the curve related to Fig. 1(c). Interestingly, the first-passage probability curves corresponding with excluded regions, shown in Fig. 1(b), and the homogeneous case are almost indistinguishable from each other for two decades. While excluding parts of the lattice increases the lengths of some paths to the targets, it also reduces the overall space that can be explored. For the setup chosen, these two effects counteract each other at short and intermediate timescales. One mode of providing a mathematical basis for this effect would be to study the eigenvalues of two systems, one homogenous and one with a reflecting point, i.e., excluded area. Since the eigenvalues of the first-passage probability distributions are known to be interlaced [76], one would expect to see that the interlacing is only marginally affected.

Among all the curves, the case with open partitions related to Fig. 1(a), results in a first-passage probability, which is the slowest to rise and with the broadest tail in the distribution. The reasons for such characteristics compared to all other curves is due to the location of the initial site relative to the targets. As the latter ones are partially behind partitions, the more directed paths take more time to reach the targets and the walker remains confined in the region around the initial site for much longer.

The sticky sites in Fig. 1(d) have limited effect on the more directed paths connecting the starting site and the targets. This is why $F_{n_{0}}\left(n_{1},n_{2},t\right)$ in Fig. 7(b) is identical to the homogeneous case at short times. However, sticky sites can be both a hindrance or a benefit to the searcher. While it can partially trap the walker and stop it from reaching the target site, it can also stop the walker from exploring regions away from the targets. Since there are sticky sites close to the targets, these two effects counteract one another and we observe marginal difference in the tail of the distribution when compared with the homogeneous curve.

### A Explicit mean first-passage quantities

The first moment of $F_{n_{0}}(n,t)$, that is the mean first-passage time (MFPT), $F_{n_{0}\to n}={\frac{\text{d}}{\text{d}z}{\tilde{F}}_{n_{0}}(n,z)|}_{z=1}$ , is given by

(8) $$F_{n_{0}\to n}=\frac{{ℱ}_{n_{0}\to n}\left|\underline{𝓗}-1/{ℱ}_{n_{0}\to n}{\underline{𝓗}}^{\left(1\right)}\right|}{\left|\underline{𝓗}-{\underline{𝓗}}^{\left(2\right)}\right|},$$

where ${ℱ}_{n_{0}\to n}$ is the homogeneous MFPT from ***n***_0_ to ***n*** and the elements of the matrices, 𝓗,𝓗^(1)^, and 𝓗^(2)^ are defined in terms of homogeneous MFPTs. They are given, respectively, in Eqs. (A3)–(A5) for general heterogeneities. In the coming sections we use the mathfrak notation, e.g., 𝔉, ℜ, and 𝔈, for statistics involving the heterogeneous dynamics, while the mathcal notation, e.g., *ℱ, ℛ*, and *ℰ*, is reserved for the homogeneous counterpart. The dependence on the target at ***n*** is only present in the matrices 𝓗^(1)^ and 𝓗^(2)^; the dependence on the initial condition ***n***_0_ is only present in 𝓗^(1)^; and the dependence on the location of the heterogeneities is in all three matrices.

The probability distribution of the first-return time is related to the propagator via $\tilde{\mathbb{R}}(n,z)=1-{\tilde{\Phi }}_{n}^{-1}(n,z)$, with a mean return time (MRT) given by

(9) $${ℜ}_{\text{n}}=\frac{{ℛ}_{\text{n}}|𝓗|}{\left|\underline{𝓗}-{\underline{𝓗}}^{\left(2\right)}\right|},$$

where *ℛ*_***n***_ is the homogeneous mean return time.

When the heterogeneities preserve the symmetric properties of the homogeneous lattice, i.e., the disorder does not add any bias to a diffusive system or remove any bias present in a system with drift, then the ratio ***A***_***u***,***v***_ */****A***_***v***,***u***_ = (***A***_***u***,***v***_ − λ_***u***,***v***_)*/*(***A***_***v***,***u***_ − λ_***v***,***u***_) is satisfied, for all {***u, v***} ∈ *S*, and the heterogeneous system maintains the steady state of the homogeneous system. In this case, 𝓗^(2)^ = 0, the MFPT given by Eq. (8), can be simplified to $F_{n_{0}\to n}={ℱ}_{n_{0}\to n}-1+|\underline{𝓗}-{\underline{𝓗}}^{\left(1\right)}|/|\underline{𝓗}|$, while the MRT remains the same as the homogeneous MRT, ℜ_***n***_ = *ℛ*_***n***_ as expected from Kac’s lemma [77] (see Appendix A 1).

In the presence of multiple targets at the outer boundary of the domain, we relate the first-passage probability to any of the targets to a propagator with the appropriate absorbing boundaries. In this case, the first passage is referred to as the first exit, and its probability generating function is related to the propagator through the relation ${\tilde{E}}_{n_{0}}(z)=1-(1-\;z)\;{\tilde{S}}_{n_{0}}(z)$, where ${\tilde{S}}_{n_{0}}(z)$ is the survival probability. Taking the mean of the distribution (see Appendix A 2) gives

(10) $$E_{n_{0}}={\tilde{S}}_{n_{0}}(z=1)={\frac{{ℰ}_{n_{0}}\left|\underline{H}-1/{ℰ}_{n_{0}}\underline{S}\left(n_{0}\right)\right|}{\left|\underline{H}\right|}|}_{z=1}$$

where ${ℰ}_{n_{0}}$ is the mean exit time starting at ***n***_0_ without any heterogeneities, and the matrix ***S***(***n***_0_) is given explicitly in Eq. (A11). The presence of one or more absorbing boundaries on the homogeneous propagator ${\tilde{\varphi }}_{r}(s,z)$ allows for a simple evaluation at *z* = 1. That is to say ${\tilde{\varphi }}_{r}(s,z=1)$ is finite for any *r* and *s* in the domain; and therefore ***H*** and ***S*** (***n***_0_) also remains finite and can be easily evaluated.

**Fig. 8:**
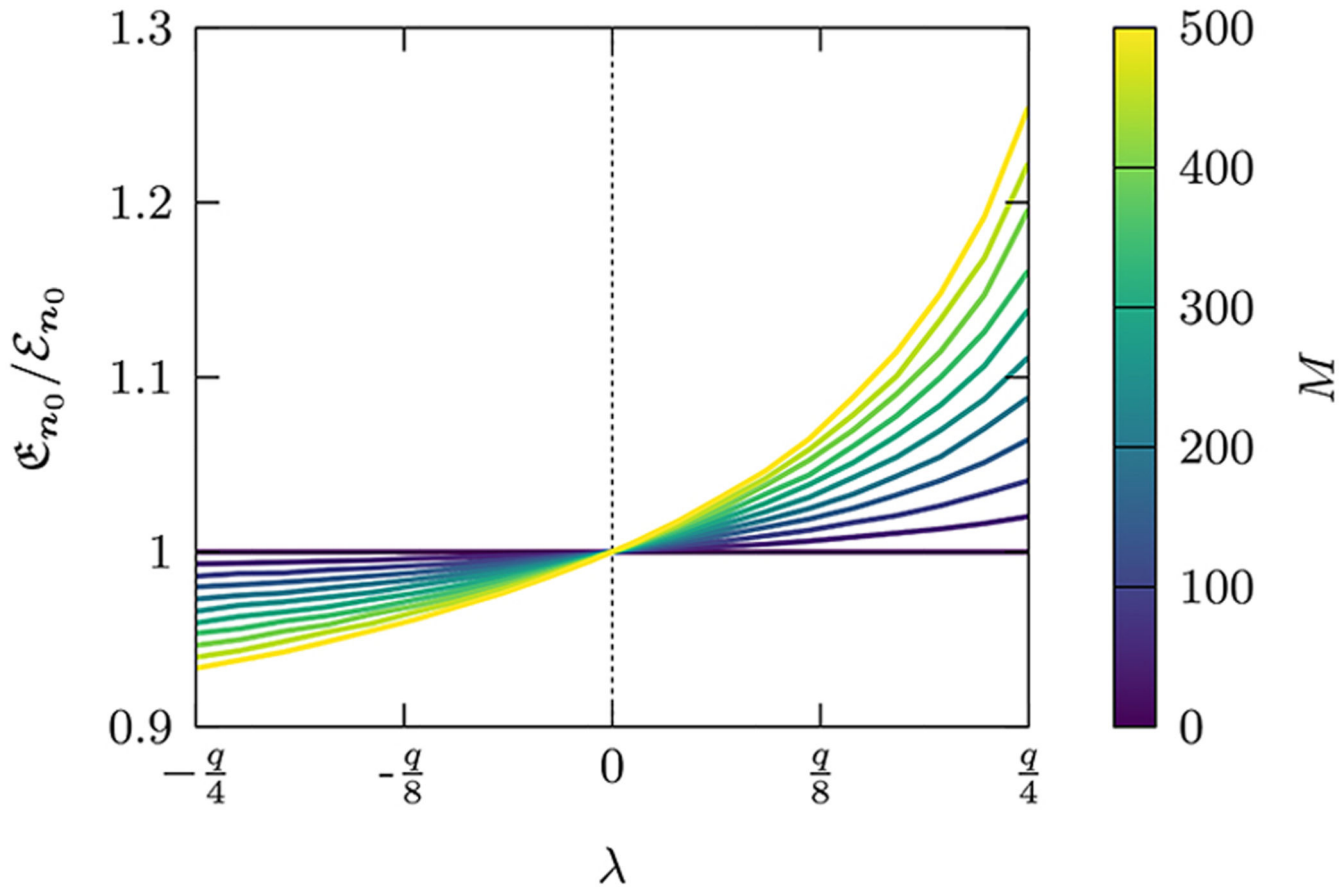
The ratio of the heterogeneous mean exit time $E_{n_{0}}$ to the homogeneous mean exit time ${ℰ}_{n_{0}}$ for randomly distributed barriers and antibarriers. We use a homogeneous propagator with a domain of size ***N*** = (51, 51) with absorbing boundary conditions, an initial condition at the center of the domain ***n***_0_ = (26, 26) and a diffusion parameter of ***q* =** (0.8, 0.8). The explicit form of ${\tilde{\varphi }}_{n_{0}}(n,z)$ is given by the *z* transform of Eq. (23) of Ref. [68]. Each curve is obtained using Eq. (10) and performing an ensemble average with 10^2^ sample realizations of locations of barriers (*λ >* 0) or antibarriers (*λ >* 0) for each λ.

In Fig. 8 we show the effect of randomly distributed barriers and antibarriers as a function of the barrier strength in a 2D domain with absorbing boundaries. The *M* neighboring defective site pairs are uniformly distributed on the lattice with λ = λ_***v***,***u***_ = λ_***u***,***v***_ for all {***u, v*}** ∈ *S*. One can see that for λ > 0, $E_{n_{0}}$ increases, as the heterogeneous connections behave as a partially reflecting barrier slowing down the walker. Furthermore, an increase in the number of heterogeneities results in larger exit times. Conversely, when *λ <* 0 the heterogeneous connections become antibarriers increasing the probability of jumping across compared to the homogeneous case, which effectively increases the spread of the walker leading to shorter exit times. When the barriers are impenetrable, increasing the number of barriers also increases the likelihood of the walker being trapped and unable to reach the boundary and will cause the mean exit time (MET) to diverge. Although we do not study it here, a similar setup could be used to analyze percolation in finite multidimensional domains.

### B First-passage processes in 1D with a single barrier and the phenomenon of disorder indifference of the MFPT

We consider a simple spatial heterogeneity in a 1D domain with a partially reflecting barrier between *u* and *u* + 1. To study the dependence of the position and strength of the barrier (or anti-barrier) on the first-passage dynamics, we first fix the position of the target and initial sites with *n > n*_0_; assume a reflecting boundary between *n* = 0 and *n* = 1; and take λ_*u*,*u* + 1_ = λ_*u* + 1,*u*_ = λ with λ ∈ [ − (1 − *q*), *q/*2]. In this case, the first-passage probability can be written using the convenient notation

(11) $${\tilde{F}}_{n_{0}}(n,z)=\{\begin{array}{c} \frac{a\left(n_{0},z\right)-\frac{2\lambda }{q}b\left(n_{0},u,z\right)}{a(n,z)-\frac{2\lambda }{q}b(n,u,z)}\;\;\;\;\;\;\;\;\;\;\;u<n_{0}\\ \frac{a\left(n_{0},z\right)-\frac{2\lambda }{q}a\left(n_{0},z\right)}{a(n,z)-\frac{2}{q}b(n,u,z)}\;\;\;\;\;\;\;\;\;\;\;\;\;u⩾n_{0} \end{array},$$

where $a(n,z)=\text{cosh}\left[\left(\frac{1}{2}-n\right)\zeta \right]\text{cosh}\left[\frac{1}{2}\zeta \right],\;b(n,u,z)=\text{cosh}[(1-n)\zeta ]+\text{sinh}\left[\left(n-2u-\frac{1}{2}\right)\zeta \right]\text{sinh}\left[\frac{1}{2}\zeta \right],\;\zeta =\text{acosh}\left[1-\frac{1}{q}\left(1-\frac{1}{z}\right)\right]$, and with the probability of moving given by *q* ∈ (0, 1]. The homogeneous first-passage probability ${\tilde{F}}_{n_{0}}(n,z)$ can be recovered from Eq. (11) by letting λ → 0. When the barrier is to the left of the initial condition, the limit $\lambda \to \frac{q}{2}$ creates an impenetrable barrier, the behavior is equivalent to shifting both the target and the initial condition to the left by *u* giving ${\tilde{F}}_{n_{0}}(n,z)={\tilde{F}}_{n_{0}-u}(n-u,z)$ . Whereas, when *n*_0_ ⩽ *u < n*, the same limit gives ${\tilde{F}}_{n_{0}}(n,z)=0$ as the walker becomes blocked by the barrier and can never reach the target.

**Fig. 9:**
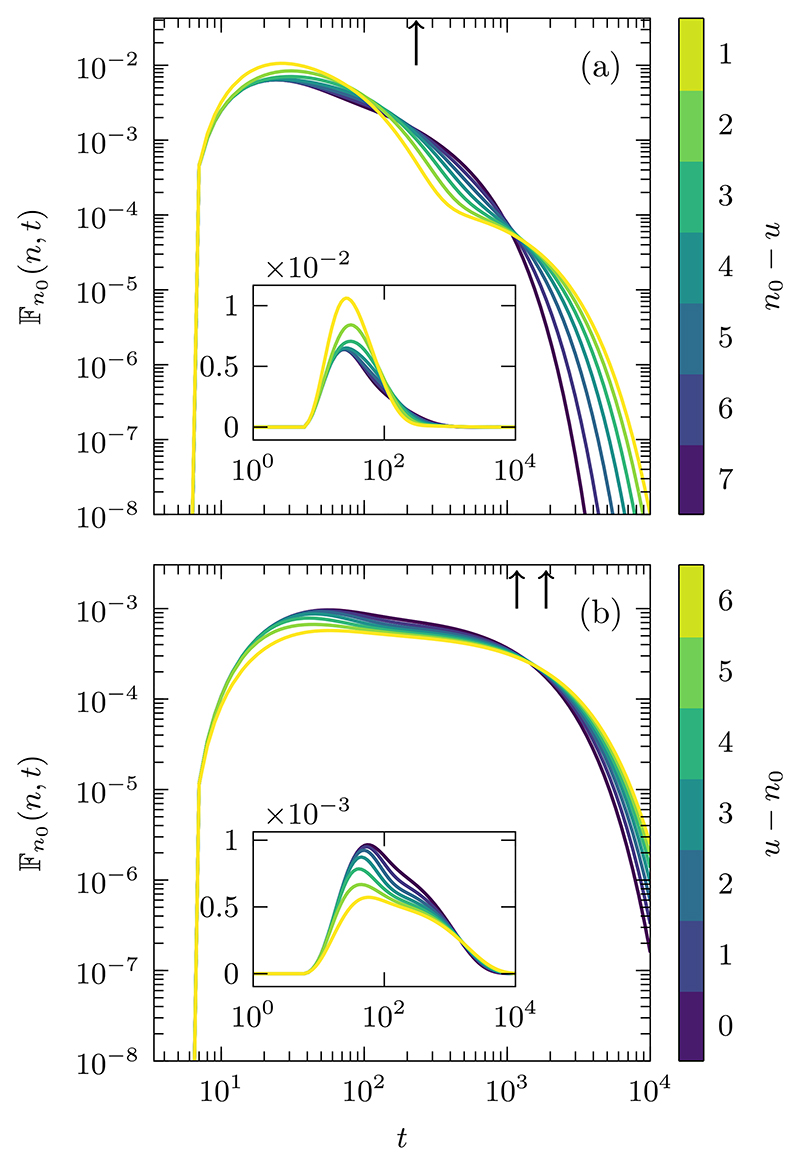
The time-dependent first-passage probability distribution is given through the numerical inversion of Eq. (11). Panel (a) represents the scenario *u < n*_0_ *< n*, while panel (b) is the case when *n*_0_ ⩽ *u < n*. The values of the other parameters are: $\lambda =0.975\times \frac{q}{2}$; a diffusion parameter of value $q=\frac{2}{3}$; the initial condition *n*_0_ = 8; the target site *n* = 15; and a reflecting boundary between *n* = 0 and *n* = 1. The arrows indicate the MFPTs: in panel (a) all of the curves have the same MFPT of $F_{n_{0}\to n}=231$ (disorder indifference), whereas in panel (b) the two arrows indicate the minimum and maximum MFPTs of the curves, which are $F_{n_{0}\to n}=1167$ and $F_{n_{0}\to n}=1869$, and obtained, respectively, when *u* = 8 and *u* = 14

In Fig. 9, we plot the time dependence of Eq. (11) for the two different scenarios *u < n*_0_ and *u ⩾ n*_0_ represented, respectively, by panels (a) and (b). With *u < n*_0_ and *λ < q/*2, that is the barrier to the left of the initial condition, as one increases *u* from *u* = 1 one observes an increase in the modal peak. When the walker is reflected by the permeable barrier, it stops the walker from straying further left and effectively reduces the space that can be explored, increasing the probability of reaching the target at an earlier time. However, if the walker passes through the barrier, the partial reflection dynamics becomes a hindrance: the walker is kept in the range [1, *u*], causing the probability of reaching the target at long times to increase also. As probability in the tail and the mode increases, the probability conserving $F_{n_{0}}(n,t)$ demands a reduction at intermediate times, which is clearly visible from the figure. This permeability induced mode-tail enhancement can also be witnessed by fixing *u* and changing λ ∈ [0, *q/*2), and we have also observed the inverse effect, mode-tail compression by having anti-barrier with λ ∈ [*q* −1, 0]. We have chosen not to display these latter cases for want of space. Similar features have been observed in a diffusing-diffusivity model in Ref. [42], where increases in the probability at short and long timescales were attributed to the dynamic diffusivity. Our findings point to the fact that such richness can also emerge from a static disorder at a single location.

Differently from the case when the barrier is to the left of the initial condition, is the case when *u* ⩾ *n*_0_. In this scenario, the barrier is always acting to slow the search process down, reducing the probability of reaching the target at early times and increasing the probability at long times as seen by the flattening of the mode and the broadening of the tail, as shown in Fig. 9(b).

Computing the mean via either Eq. (11) or from simplifying Eq. (8) yields the compact expression

(12) $$F_{n_{0}\to n}={ℱ}_{n_{0}\to n}+\frac{2}{q}\frac{\lambda }{\frac{q}{2}-\lambda }\{\begin{array}{c} 0,\;\;\;\;\;\;\;\;\;u<n_{0},\\ u,\;\;\;\;\;\;\;\;\;\;u⩾n_{0}, \end{array}$$

where ${ℱ}_{n_{0}\to n}=\left(n-n_{0}\right)\left(n+n_{0}-1\right)/q$ is the 1D homogeneous MFPT for *n*_0_ ⩽ *n* {given by Eq. (12) of Ref. [68]}. Astonishingly, the mode-tail enhancement present in the time-dependent probability when *u < n*_0_ has no effect on the mean. This is what we have termed the disorder indifference phenomenon.

To explain why there is such an effect of disorder indifference, we split the first-passage trajectories into two mutually exclusive subsets: the trajectories that never return to the initial condition before reaching the target site on the right and those that return at least once before reaching the target site. Clearly, the former trajectories are unaffected by the presence of a barrier. The latter trajectories *can* be affected by the barrier; however, in computing the mean one deals with mean return times, which are unaffected from the homogeneous case when λ_*u*,*u*+1_ = λ_*u*+1,*u*_ as stated in the previous section (see Appendix B1 for the mathematical details).

An analog of this indifference phenomenon was observed in Ref. [50], where the MFPT in a quasi-1D domain in continuous space with two layers of different diffusivity was studied. When the initial condition was in between the interface of the layers and the target, they observed that the MFPT was indifferent to the diffusivity of the media beyond the interface. In that study, the first-passage probability was not considered and the cause of this indifference could not be quantified. However, one can relate the location of the interface of their system with the position of the barrier in ours. Through this relation, we believe that the behavior observed in Ref. [50], is closely related to the dynamics presented in Fig. 9.

Given a barrier between the initial condition *n*_0_ and the target *n*, the effect on the MFPT increases linearly as the displacement from the boundary increases. While the effect, which can be to speed up (*λ <* 0) or to slow down (*λ >* 0), is due to the disorder, the linear dependence is not. This linear dependence is present in all 1D situations and is proportional to the distance between the initial condition and the reflecting boundary (see Appendix B 2).

To explore the effects of asymmetry in the heterogeneities we consider the MRT with λ_*u*+1,*u*_ *≠ λ*_*u*,*u*+1_. In this case, the steady state is no longer homogeneous. To illustrate this point, we consider the MRT of a 1D walker within a segment of length *N* with reflecting boundaries and with a barrier between *u* and *u* + 1, (λ_*u*+1,*u*_ *≠ λ*_*u*,*u*+1_). In this case, Eq. (9) simplifies to

(13) $${ℜ}_{n}=\{\begin{array}{c} N\left[\frac{q/2-\lambda_{u+1,u}}{q/2-\lambda_{u,u+1}}\right]-u\left[\frac{\lambda_{u,u+1}-\lambda_{u+1,u}}{q/2-\lambda_{u,u+1}}\right],\;\;\;\;\;\;\;n<u+1,\\ N-u\left[\frac{\lambda_{u,u+1}-\lambda_{u+1,u}}{q/2-\lambda_{u+1,u}}\right],\;\;\;\;\;\;\;\;\;\;\;\;\;\;\;\;\;\;\;\;\;\;\;\;\;\;\;\;\;\;\;\;\;\;\;\;n⩾u+1, \end{array}.$$

One can see that when λ_*u*+1,*u*_ = λ_*u*,*u*+1_, the MRT reduces to *N* regardless of whether *n ⩽ u* or *n > u*. In the extreme case, where the barrier is impenetrable in both directions, λ_*u*+1,*u*_ = λ_*u*,*u*+1_ = *q/*2, one can recover the appropriate MRTs when *n ⩽ u* and *n > u*, which are, respectively, *u* and *N* − *u*.

## V Computational Advantage Over Existing Methods When Computing First-Passage Statistics

It is possible to gain a significant computational advantage when calculating first-passage statistics using the explicit expressions given in Eqs. (8)–(10), instead of employing numerical or Monte Carlo approaches. To illustrate this aspect, consider for the sake of simplicity, a *d* lattice with a width of *N* sites and periodic or reflecting boundary conditions. In this case the computation to determine the MFPT to a single target via Eq. (8) has a time complexity of *M*^2^*N*^*d*^ ⩽ *N* ^2.373*d*^ using a naive implementation as the one given in Appendix H.

For alternative procedures that is in the absence of explicit knowledge of the first-passage statistics expressions, one can approach the search problem in one of two ways: either via the numerical scheme described in Ref. [78] or via Monte Carlo agent-based simulations. The first procedure requires one to compute first the time-dependent first-passage dynamics via the iteration of the Master equation, giving a time complexity of *N* ^2*d*^*t*, where *t* is determined by an appropriate criterion, e.g., after when the first-passage probability is below some threshold. While this is already computationally more expensive than the naive implementation of Eq. (8), one has to also consider the nontrivial task of defining a stopping criterion, which in general varies depending on the specific scenario due to the richness of first-passage dynamics, e.g., bimodality [69], and multiple timescales [79].

While Monte Carlo simulations may appear not to suffer from the challenges of the numerical scheme described above, they have two fundamental drawbacks. The first is that stochastic spatiotemporal simulations, having large trajectory-to-trajectory fluctuations, require a very large ensemble size leading to long simulation times. The second and more pertinent issue is that one cannot systematically reduce the error between the true observable and the ensemble estimate. It is thus difficult to define *a priori* the size of the ensemble required to get a prescribed minimum accuracy. Since this problem is already present when the space is homogenous [80], it is exacerbated when different heterogeneities are present, making it difficult to explore the entire parameter space.

Faster computational procedure can also be exploited to evaluate (5), (8) and (9), i.e., the homogeneous propagator and mean first-passage times, by casting them as an inverse cosine transform problems [81]. This allows one to expedite the computation of the matrix elements using the inverse fast Fourier transform [82], reducing drastically the complexity to *N*^*d*^ log_2_ *N*^*d*^*t* for the propagator and *N*^*d*^ log_2_ *N*^*d*^ for the first-passage statistics.

Thus far we have focused on technical development and theoretical insights. As we move forward, the remainder of the article is devoted to practical examples and is used to demonstrate the applicability of the framework. For practical convenience, the details of the modeling set-up are given in the Appendixes and only the results are discussed.

## VI Transdermal Drug Delivery

In the first application we consider the problem of optimizing transdermal drug delivery, that is the transfer of drugs through the skin. One of the challenges of transdermal drug delivery is traversal of the outermost layer of the epidermis called the stratum corneum (SC) by hydrophilic molecules [83]. This layer is made up of dead cells called corneocytes, which are arranged in a dense “brick-and-mortar” like pattern [84]. Inspired by some of the recent strategies proposed to enhance drug absorption [85], we consider the use of microneedles to pierce first the SC before applying a drug patch. We study the effectiveness of this method by using our modeling framework to represent the SC as heterogeneities on a lattice and modeling the movement of drug molecules as a random walk.

**Fig. 10:**
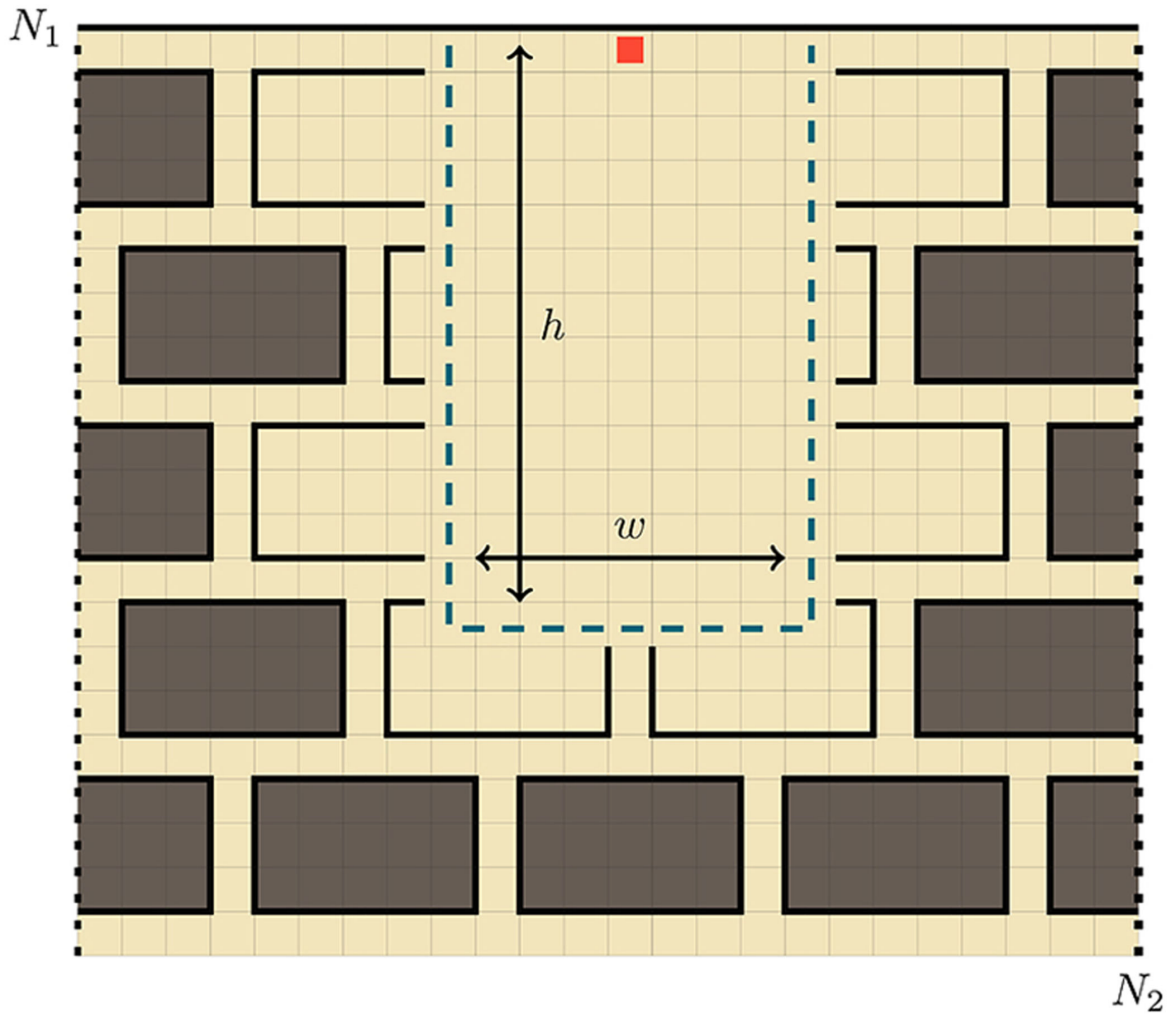
Representation of the “brick-and-mortar” arrangement of the corneocytes in the stratum corneum (SC). The red square depicts the starting location of the random walker. The geometry is given by an an absorbing boundary at *n*_1_ = 1, a reflecting boundary at *n*_2_ = *N*_1_, shown as a thick solid black line, and a periodic boundary in the second dimension, depicted as dashed black lines. By using a number of paired defects, one is able to cordon off sites (shaded grey), creating the “brick-and-mortar” pattern of the SC. The dashed blue rectangle with width *w* and height *h* models the destruction of the SC structure via a microneedle puncture, with *h* and *w* representing, respectively, the puncture height and width. This destruction may open up some of the “bricks”, allowing the walker to easily travel inside. The initial position of the walker is at the center-top of the puncture, ***n***_0_ = (*N*_1_, *N*_2_*/*2).

We use a homogeneous 2D nearest-neighbor random walker subject to mixed boundary conditions: an absorbing boundary located at *n*_1_ = 1 and a reflecting boundary located at *n*_1_ = *N*_1_, for the first dimension and a periodic boundary condition on the second dimension. The heterogeneities are impenetrable barriers representing the lipid matrix. These are arranged in a manner to create excluded regions that form the brick-and-mortar pattern of the SC, see Fig. 10. The pattern is partially destroyed to represent the needle piercing in a rectangle with height *h* and width *w* resulting in an area absent of barriers as shown by the blue dashed rectangle in Fig. 10.

**Fig. 11:**
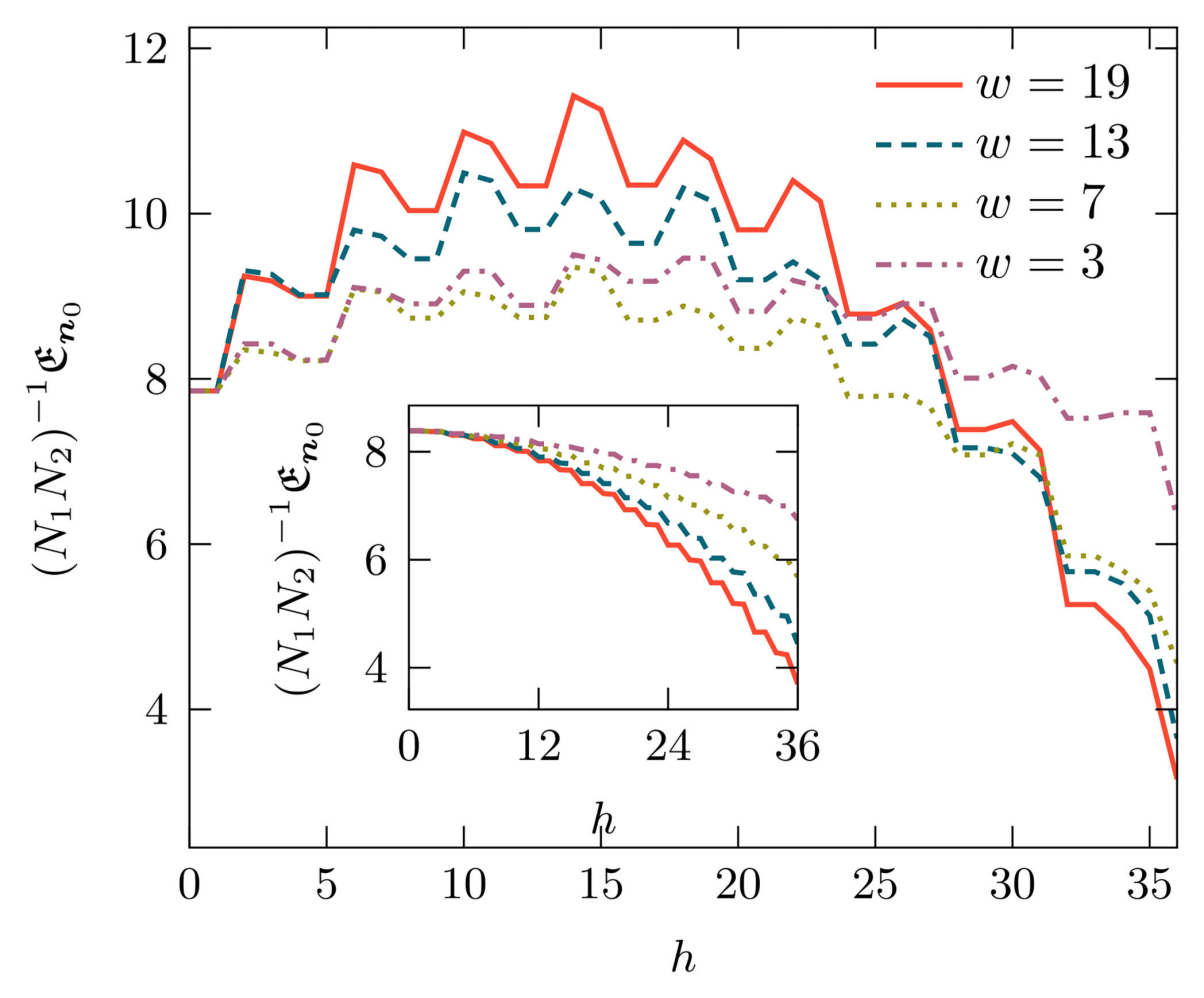
Mean exit time as a function of the puncture height *h* for different values of puncture width *w* (see Fig. 10 for description of the setup). We use a rectangular domain of size ***N*** = (37, 36), a diffusion parameter of ***q*** = (0.8, 0.8), “bricks” of size (3, 5) resulting in nine layers with six bricks per layer, an initial condition of ***n***_0_ = (1, 19). The main panel depicts the scenario where the barriers encapsulating the “bricks” are impenetrable, i.e., *α*_***v***_ = *α*_***v***_ = 1 leading to λ_***v***,***u***_ = λ_***u***,***v***_ = 0.2 for all ***u, v***, while the inset shows the scenario where the barriers are partially permeable with *α*_***v***_ = *α*_***v***_ = 1 giving λ_***v***,***u***_ = λ_***u***,***v***_ = 0.18.

The quantity of interest is the MET with an initial condition starting at the reflecting end of the domain. We plot the MET as a function of the puncture depth and width in Fig. 11. The overarching qualitative changes in the MET can be explained by two competing effects. The first is the breaking of enclosed bricks to create open partitions. The additional sites available for exploration make the paths to reach the absorbing boundary longer.

The second effect is that the puncture allows the walker more direct movement towards the bottom layers leading to smaller MET. The removal of some of the impenetrable barriers allows for more direct paths to the absorbing boundary, which leads to smaller mean exit times. The strength of this effect is dependent on the size of the puncture *hw*. For small values of *h*, the first effect has greater influence leading to an increase in the METs. As *h* is increased the second effect becomes more prominent and drives down the METs resulting in a global maximum. The interplay between the two effects also gives rise to the oscillations. The puncturing of a brick layer opens it up, leading to larger exit times as the walker becomes temporarily confined inside a brick. Increasing the puncture height further destroys the brick structure of a layer and allows the walker to traverse the latter via a direct route thereby decreasing the exit times.

The global maximum and the oscillations are only present when the barriers are highly reflecting or impenetrable, i.e., 0 ⪡ λ_***v***,***u***_ λ_***u***,***v***_ ***⩽*** *q/*2 for all {***u, v*}** ∈ *S*. The maximum is lost when the permeability gets larger as the random walker is only partially confined by the barriers, leading to a monotonic decrease in the MET as seen in the inset of Fig. 11. With permeable barriers all the sites are always accessible independently of *h* and *w*, puncturing only creates more direct routes to the absorbing boundary leading to smaller exit times.

## VII Thigmotaxis

For the second application, we look at thigmotaxis, which broadly speaking, is the movement of an organism due to a touch stimulus. We are interested specifically in the tendency of animals to remain close to the walls of an environment, a behavior that is observed in many species from insects to mammals [86,87]. We quantify the thigomotactic tendency by appropriately selecting defects location and λ to represent regions, which are more easily accessible when moving in one direction (approaching boundaries) versus another (moving away from boundaries).

Since we are able to construct arbitrary shapes with the formalism, we consider two concentric circles within a square domain. The first is used to restrict the walker to a circular reflecting domain of radius *R*. The second has a radius *r*, with *r < R*, and is used to partition the domain into two regions: an inner region; and an outer region, which is the annulus between *r* and *R*, representing the preferred area of occupation. By placing one-way partially reflecting barriers along the radius *r*, we allow the walker to leave the inner region to enter the outer region without any resistance, while the tendency of remaining in the outer region is controlled by the parameter *α*_*i*_ ∈ [0, 1]. With *α*_*i*_ = 1 the walker never leaves the outer region once it gets there, whereas with *α*_*i*_ = 0, the partially reflecting barriers are removed and all areas of the circular domain become easily accessible. For the details on the placement of the defects and the construction of the circular domain see Appendix C 1.

**Fig. 12:**
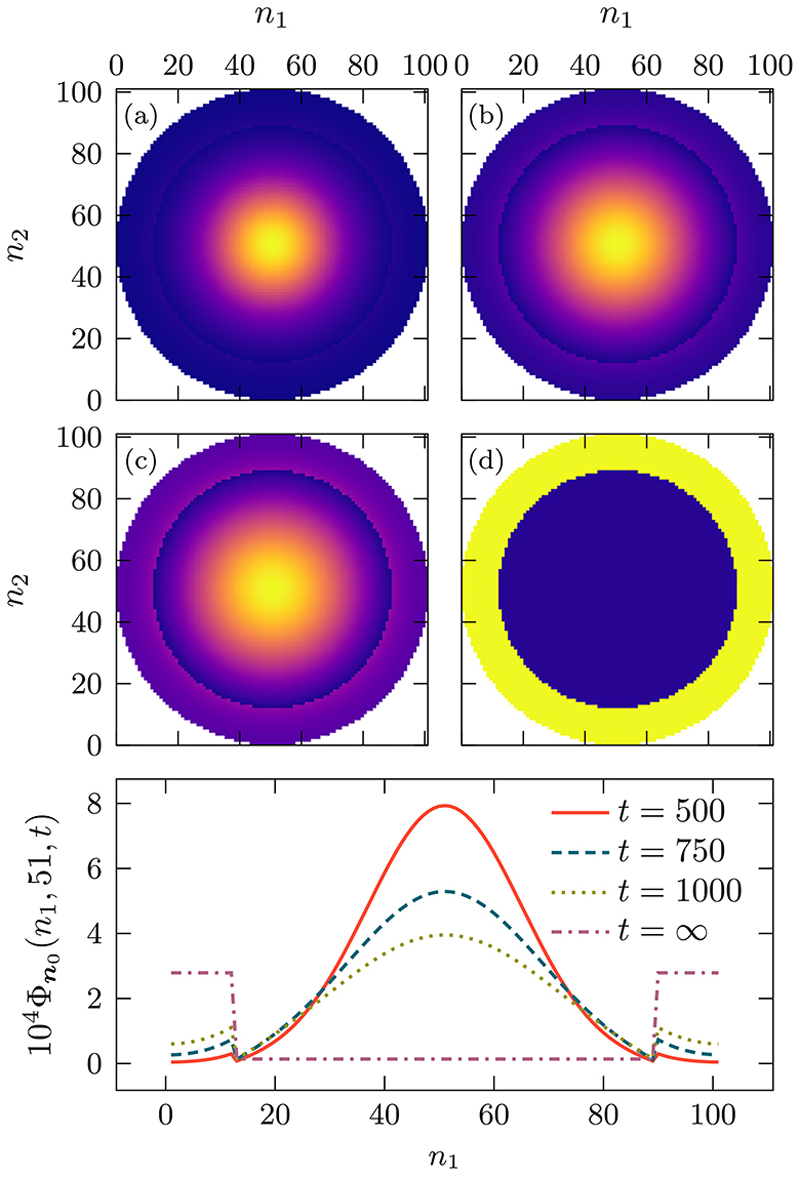
The propagator ${\text{Φ}}_{n_{0}}(n,t)$ at different moments in time, *t* = 500, 750, 1000, ∞ where the walker is initially at the center of the domain, ***n***_0_ = (51, 51). When inside the inner region the walker can freely enter the outer region without any resistance, that is λ_***v***,***u***_ = 0 and when in the outer region the probability to move inward is modified via $\lambda_{u,v}=\alpha_{i}{\underline{A}}_{v,u}$. Other parameters used are the diffusivity of value ***q*** = (0.8, 0.8) and a square domain of size ***N*** = (101, 101), (see Appendix C1 for details on the placement of defects)

Given these constraints, we study the dynamics as a function of the *α*_*i*_. In Fig. 12, we plot the probability ${\text{Φ}}_{n_{0}}(n,t)$ for different values of *t* = 500, 750, 1000, ∞ and. The walker is initially at the center of the domain ***n***_0_ = (51, 51) and can freely move inside the inner region and is able to enter into the outer region without any resistance. However, once inside the outer region there is a greater tendency not to leave, due to the high value of *α*_*i*_ = 0.95. We observe this effect when going from panel (a) to (d). Initially, the separation between the inner and outer regions is barely visible but as time progresses this separation becomes increasingly clear, culminating with a sharp step at the steady state. In panel (e) we plot a cross section of the probability at *n*_2_ = 51 for the times corresponding with panels (a)–(d).

**Fig. 13:**
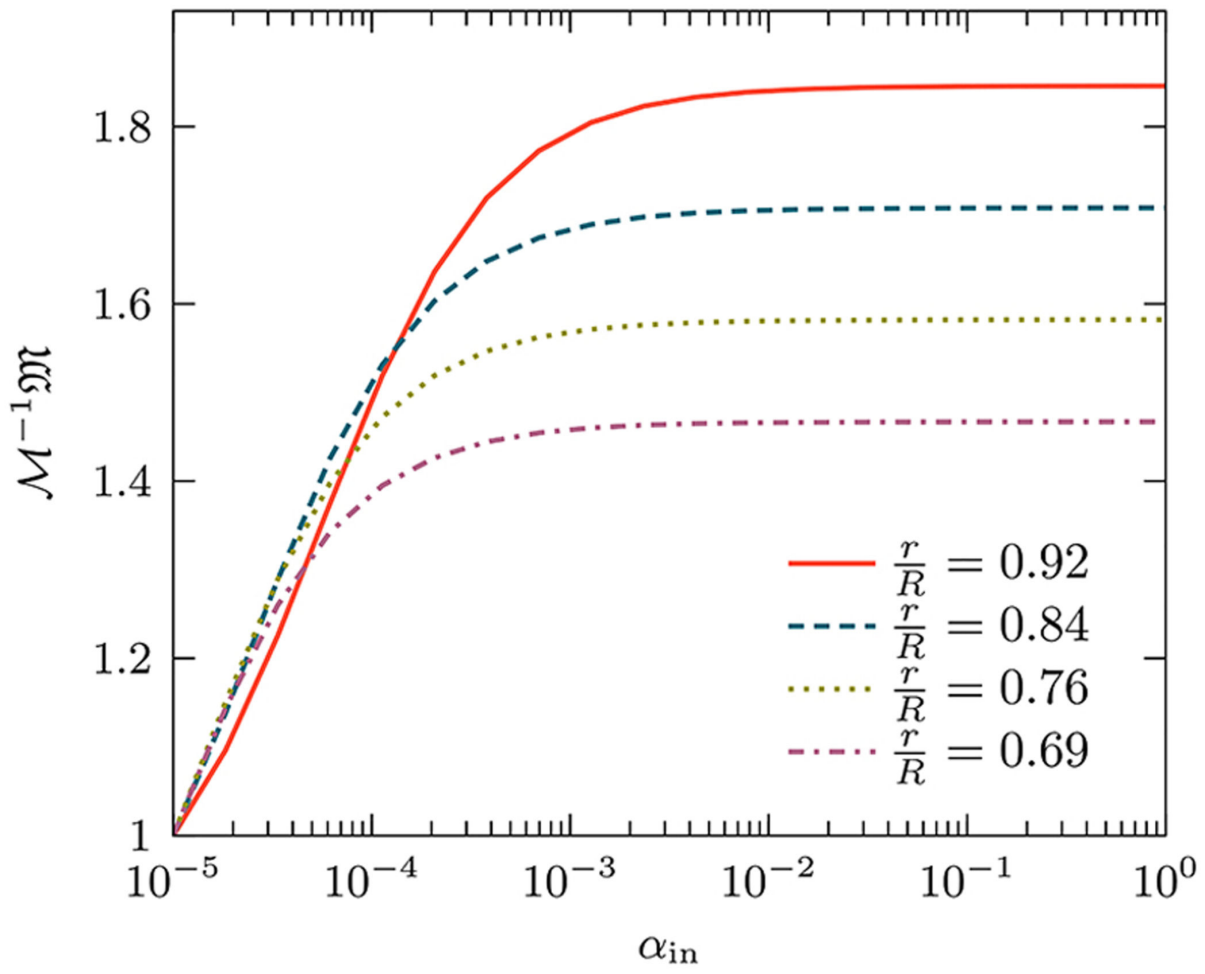
Saturated mean-squared displacement for a thigmotaxis process for different values of the ratios of *r/R*. We study the dynamics as a function of the normalized parameter *α*_*i*_ ∈ [0, 1], which represents the tendency of the walker to remain close to the boundary. When *α*_*i*_ = 0, there are no outer or inner regions, while with *α*_*i*_ = 1, the walkers never leave the outer region once they get there. The saturation MSD is normalized by ℳ, which is the saturation value when *α*_*i*_ = 0. Other parameters used are described in the caption of Fig. 12.

To examine the system further, we plot in Fig. 13 the meansquared displacement (MSD) at steady state 𝔐 as a function of *α*_*i*_, for four different ratios of inner and outer regions. The MSD at steady state is given by $M={\text{Σ}}_{\text{n}}[{\left(n_{1}-n_{0_{1}}\right)}^{2}+(n_{2}-{n_{0_{2}})}^{2}]{ℜ}_{n}^{-1}$. With the curves normalized to the case where there are no internal barriers ℳ, i.e., when *α*_*i*_ 0. We find that as we increase *α*_*i*_ from zero, for small values of *α*_*i*_, 𝔐 initially increases logarithmically, while further increases of *α*_*i*_ causes 𝔐 to saturate. The value of saturation is dependent on the ratio of *r/R*: with a high ratio the outer region is thinner keeping the walker closer to the boundary and yielding greater saturation value, whereas a smaller ratio results in a thicker outer region allowing the walker to remain closer to the initial condition leading to a smaller value of M. Note that the reason for the *r/R* = 0.92 curve not being on top of the others is due to the discretization of space when the outer region is very thin.

### VIII Two-Particle Coalescing Process

In this final example, we demonstrate the use of our frame to model certain inert interactions between particles. The interactions we consider are partial mutual exclusion and reversible binding, both of which play an important role in coalescing dynamics.

Coalescing processes are ubiquitous in biology and chemistry; they consist of two or more entities that interact to bind and form a new one with different movement characteristics. An example of a coalescing process is the search of a promoter region on DNA by transcription factors. These movement dynamics alternate between periods of 3D search in the cytoplasm and periods of restricted search along the 1D DNA [88]. Indeed, the reduction of dimensionality as a vehicle for accelerating up target search has been put forward before as a general concept in biology [89], and later investigated in the context of DNA dynamics [90–93]. While such studies focus on the difference between 3D diffusion of transcription factors in solution compared with reduction that occurs when diffusion along a 1D DNA strand, dimensionality reduction can also occur when one considers multiple interacting particles. We use our framework to study a system of relevance to the latter scenario: a first-passage process of two interacting particles in 1D.

**Fig. 14:**
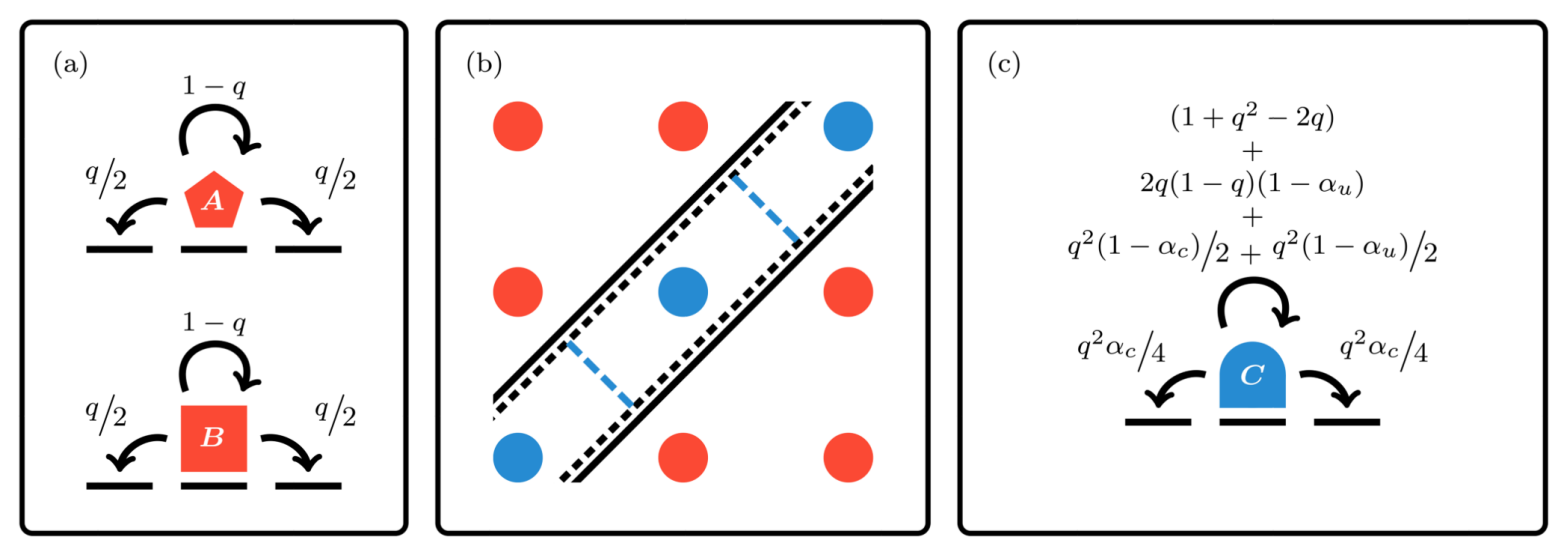
Schematic representation of a two-particle coalescing process, modelled as one-dimensional interacting random walkers. Panel (a) depicts the dynamics of particles ***A*** and ***B*** where *q* ∈ (0, 1] is the probability of moving at each time step. The combined dynamics of ***A*** and ***B*** can be represented via one next-nearest random walker in a 2D domain. This abstract domain is depicted in panel (b). The red circles represent locations where the two particles are on different sites, while the blue circles along the right diagonal are locations where they are colocated. In this space, the interaction of ***A*** and ***B*** is modelled with partially reflecting barriers. The placement of these barriers is illustrated by the solid, dashed, and blue-dashed lines, the precise locations and permeability are given in the Appendix C 2. The solid black lines are heterogeneities used to model the binding interactions, while the dashed black lines are used to control the unbinding interactions. The movement of ***C*** is represented by the 2D random walker moving along the diagonal. Its movement is slowed down, relative to ***A*** and ***B***, through the placement of partially reflecting barriers along the diagonal depicted by the dashed blue lines. The resulting movement dynamics of the complex ***C*** is shown in panel (c), where *α*_*c*_ ∈ [0, 1] represents the degree with which the movement of the complex ***C*** is slowed down.

We consider two particles labeled ***A*** and ***B*** that move independently on a 1D lattice with reflecting boundary conditions (see Fig. 14 for a schematic representation of the process). Their combined dynamics is described by a two-dimensional next-nearest propagator $\varphi_{n_{0}}(n,t)$, with $n_{0}=\left(n_{0_{1}},n_{0_{2}}\right)$ and ***n* =** (*n*_1_, *n*_2_). It represents the probability that the particle ***A*** and ***B*** are located, respectively, on the site *n*_1_ and *n*_2_ at time *t* given that they started, respectively, on $n_{0_{1}}$, and $n_{0_{2}}$. Two particles instantaneously form the complex ***C***, namely when they encounter each other, that is when ***n*** = (*m, m*) for 1 ⩽ *m* ⩽ *N*.

The interactions between particles is modelled through the placement of heterogeneities on the combined 2D lattice, yielding three control parameters, *α*_*e*_ ∈ [0, 1], *α*_*u*_ ∈ [0, 1], and *α*_*c*_ ∈ [0, 1] (see Appendix C2 for details regarding the placement of the defects). These parameters are used to constrain, respectively, the binding events via mutual exclusion of ***A*** and ***B***, the unbinding events of ***C*** and the mobility of ***C***. The parameter *α*_*u*_ is proportional to the unbinding probabilities, while *α*_*e*_ is proportional to the mutual exclusion probability. When *α*_*u*_ = 1 and *α*_*e*_ = 0, there is no interaction between the two particles. The other extreme represents strong interaction: when *α*_*e*_ = 1 there is mutual exclusion, whereas *α*_*u*_ = 0 results in a binding that is irreversible. The parameter *α*_*c*_ ∈ [0, 1] represents the fraction of the movement probability of complex ***C*** relative to the movement probability of the constituent particles ***A*** and ***B***. When *α*_*c*_ = 1 there is no slowing down, while *α*_*c*_ = 0 results in an immobile ***C***.

**Fig. 15:**
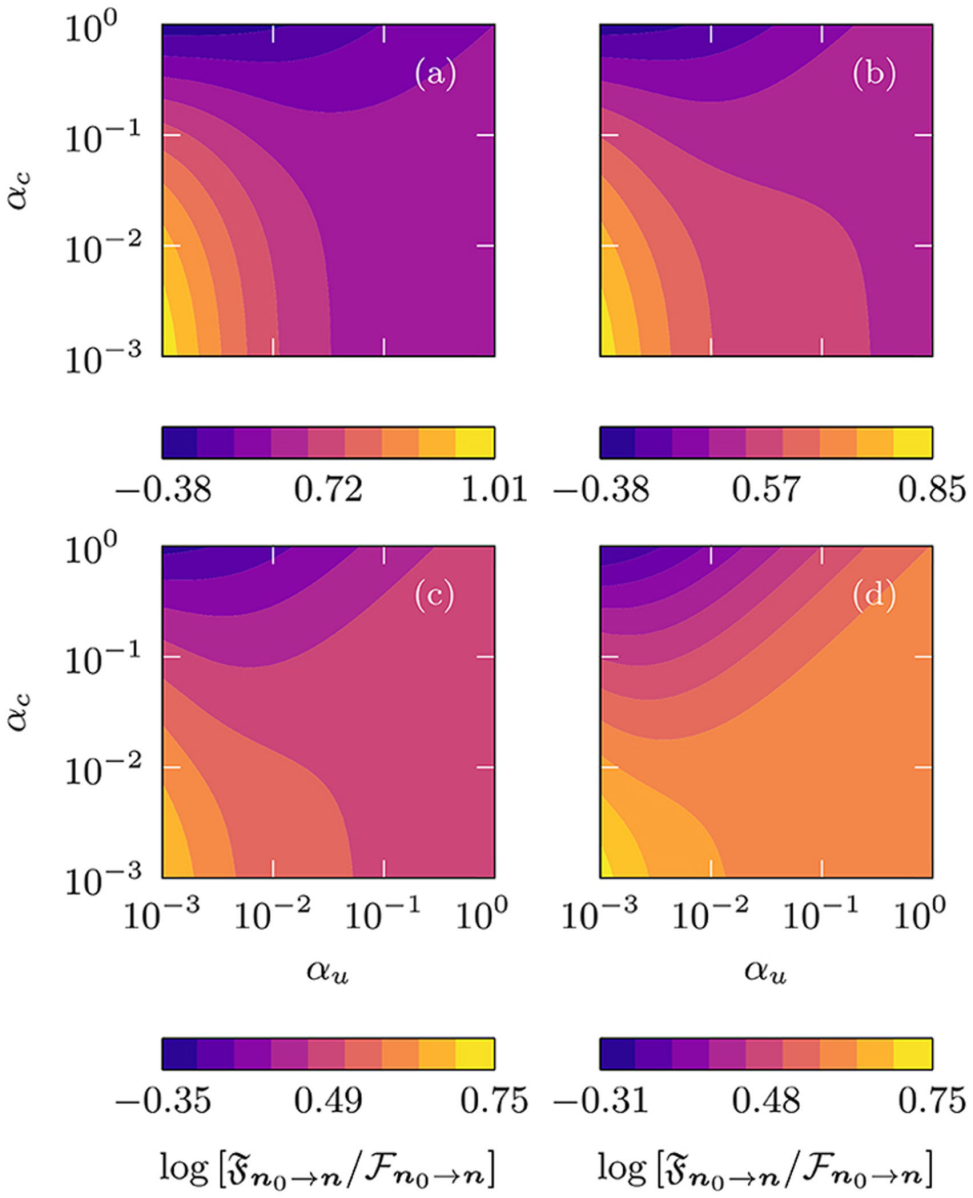
The ratio of the MFPT $\left(F_{n_{0}\to n}\right)$ of the coalescing system compared to the MFPT $\left({ℱ}_{n_{0}\to n}\right)$ of a homogeneous 2D next-nearest-neighbor walker as a function of the heterogeneity strength parameters (see Fig. 14 for detailed a description of the parameters involved). The reactive site is located at ***n*** = (100, 100) and the two particles are initially maximal distance away from each other, i.e., ***n***_0_ = (1, 100) and a target location ***n*** = (100, 100), on combined 2D domain of size ***N*** = (100, 100) with diffusion parameter *q* = 2*/*3. From panel (a) to (d) we have, respectively, the parameters *α*_*e*_ = 0, 0.5, 0.75, and 0.875.

In Fig. 15 we plot the log ratios of the MFPT, $F_{n_{0}\to n}$ for both particles to reach a site at the same time, compared with the 2D homogeneous next-nearest-neighbor analogue ${ℱ}_{n_{0}\to n}$. The latter corresponds with the case when *α*_*e*_ = 0 and *α*_*u*_ = *α*_*c*_ = 1. The panels (a)–(d) depict $F_{n_{0}\to n}$ for increasing values of *α*_*e*_. The smallest ratios are observed in the upper left quadrant, which corresponds with high cohesiveness of the complex ***C*** and with only a slight reduction to its mobility, given respectively by, low values of *α*_*u*_ and high values of *α*_*c*_. Within this parameter region, once the two particles bind they rarely separate, consequently the search in 2D reduces to a search in 1D with fewer sites to explore leading to smaller $F_{n_{0}\to n}$.

When there is no exclusion interaction, i.e., panel (a), the dynamics of a similar model was explored in Ref. [67]. In their analysis using asymptotics and simulations, equivalent features were observed. The most prominent feature of those and our observations is the minimization of the MFPT for a slow moving ***C***. In this regime, it is more favorable to have an intermediate unbinding probability, allowing the two particles to travel independently towards the target before recombining and hitting the target.

The ability to explore easily the parameter space of the model allows us to analyze the MFPT for different values of *α*_*e*_. By comparing the four panels we observe that as *α*_*e*_ increases, the overall magnitude of the MFPT ratio decreases. This is explained by the fact that for small and intermediate values of *α*_*e*_ the 2D walker is partially restricted to the upper or lower triangular regions of the domain, thereby reducing the overall exploratory space resulting in shorter search times. However, if *α*_*e*_ is increased further, i.e., when 0 ≪ *α*_*e*_ < 1, the particles will rarely coalesce, and the MFPT increases. In other words, shorter MFPTs can be achieved by having particles that mutually exclude one another with some probability.

## IX Conclusions

We have introduced an analytical framework to model explicitly any inert particle-environment interactions. The framework represents a significant advance in random walk theory. The defect technique for lattice random walks has so far only been used for locations with absorbing properties [94–96], whereas our generalization of the technique to include probability conserving, i.e., nonabsorbing defects, has facilitated new explicit expressions for propagators and various observables.

More specifically, we have constructed the discrete Master equation that describes the spatiotemporal dynamics of diffusing particles in disordered environments by representing the interactions as perturbed transition dynamics between lattice sites. To solve this Master equation we have generalized the defect technique to yield the generating function of the propagator in closed form. Using the propagator, we have derived useful quantities in the context of transport processes, namely, first-passage, return, and exit probabilities and their respective means. We have also uncovered the temporal dynamics that lead to the disorder indifference phenomenon of the mean first-passage time in quasi-1D systems.

Our framework is relevant to many empirical scenarios as one can represent in, great generality, environmental features that impede or promote movements. The presence of many such features are now readily observed at all scales, e.g., permeable boundaries at the interface for two different tissue, or the boundaries between neighboring territories of animals. In order to apply our framework to such scenarios, the modeling challenge is not in the discretization of spatiotemporal observations where the former naturally emerges from the resolution of the measuring apparatus, but in the appropriate choice of transition probabilities that define how an agent moves and interacts with the environmental features. However, this is only a minor inconvenience as one can proceed by defining the movement changes due to spatial heterogeneities relative to a diffusion coefficient in a homogeneous environment. Given that such a diffusion coefficient has been measured in a wide variety of scenarios, our framework allows testing of various properties of spatial heterogeneities and their impact on transport statistics.

In order to demonstrate such versatility, we have chosen three examples. In the first example, we consider transdermal drug delivery, an intercelluar transport process, where we represent the “brick-and-mortar” structure of the stratum corneum with the placement of reflecting and partially reflecting barriers. This representation allows us to study the effect that piercing has on the traversal time of a drug molecule. In the second example, we have examined the effect that an animal’s thigomotactic response has on the mean-squared displacement at log times. Lastly, in our third example, we have highlighted the ability of our formalism to study inert interactions between particles. We transformed these interactions and the ensuing dynamics into a single particle moving and interacting with quenched disorder in a higher-dimensional space. The setup allows us to model analytically the search statistics in a two-particle coalescing process, akin to the search of binding sites on the DNA by multiple transcription factors.

The strength of our result is in deriving the propagator in the presence of spatial heterogeneities ${\tilde{\Phi }}_{n_{0}}(n,z)$, as a function of the homogeneous propagator, i.e., the propagator in the absence of heterogeneities ${\tilde{\varphi }}_{n_{0}}(n,z)$. This modularity allows one to change the movement dynamics by selecting different forms of ${\tilde{\varphi }}_{n_{0}}(n,z)$. In place of the diffusive propagator one may employ a biased lattice random walk [69], or a walk in different topologies such as triangular lattices [96,97], Bethe lattices [98,99], or more generally a network [100].

The modularity carries through to the heterogeneous propagator. This means that in situations where homogeneous space is assumed, one can relax this assumption and replace the homogeneous propagator ${\tilde{\varphi }}_{n_{0}}(n,z)$ with the heterogeneous counterpart ${\tilde{\Phi }}_{n_{0}}(n,z)$. We have demonstrated this aspect by studying the first-passage probability to either of two targets using results previously derived considering a homogeneous lattice. Further theoretical exploration could include the analysis of cover time statistics [101,102], transmission dynamics [103,104], resetting walks [105–107], mortal walks [108], or random walks with internal degrees of freedom [109].

Directions for future applications span across spatial and temporal scales: the role of a building geometry or floor plan on infection dynamics in hospital wards and supermarkets [79,110,111]; the prediction of search pattern behavior of animals in different types of vegetation cover [112,113]; the heat transfer through layers of skin with differing thermal properties [114]; and the influence of topological defects on the diffusive properties in crystals [115,116] and territorial systems [117–119].

We conclude by drawing the reader’s attention to the following. As experimental technologies continue to evolve, observations of the dynamics of particle-environment interactions are increasing in number and resolution. The detailed description of the environment that these technologies bring presents a unique opportunity to rethink modeling techniques, moving away from macroscopic paradigms to a more microscopic prescription. We believe that the mathematical framework we have introduced to quantify the particleenvironment interactions will play a crucial role in connecting the microscopic dynamics to the macroscopic patterns observed across a vast array of systems.

## Supplementary Material

Appendix
