# Supplementary material for: Particle-environment interactions in arbitrary dimensions: A unifying analytic framework to model diffusion with inert spatial heterogeneities
Source: Phys Rev Res. Author manuscript; Available in PMC 2025 Apr 28. (PMC7617621; doi:10.1103/PhysRevResearch.5.043281)
Supplement: Appendix [file EMS204772-supplement-Appendix.pdf]

APPENDIX A: MEAN FIRST-PASSAGE STATISTICS

Using the renewal equation the first-passage probability to a target is given by the well-known relation

$$\widetilde{\mathbb{F}}_{\boldsymbol{n}_0}(\boldsymbol{n}, z) = \frac{\widetilde{\Phi}_{\boldsymbol{n}_0}(\boldsymbol{n}, z)}{\widetilde{\Phi}_{\boldsymbol{n}}(\boldsymbol{n}, z)} = \frac{(\widetilde{\varphi}_{\boldsymbol{n}_0}(\boldsymbol{n}, z) - 1)|\underline{\boldsymbol{H}}| + |\underline{\boldsymbol{H}}(\boldsymbol{n}, \boldsymbol{n}_0)|}{(\widetilde{\varphi}_{\boldsymbol{n}}(\boldsymbol{n}, z) - 1)|\underline{\boldsymbol{H}}| + |\underline{\boldsymbol{H}}(\boldsymbol{n}, \boldsymbol{n})|}, \tag{A1}$$

where $\underline{\boldsymbol{H}}$ and $\underline{\boldsymbol{H}}(\boldsymbol{n},\boldsymbol{n}_0)$ are given by, respectively, Eqs. (6) and (7) with the initial condition being $\boldsymbol{n}$. The mean of the distribution $\mathfrak{F}_{\boldsymbol{n}_0\to\boldsymbol{n}} = \frac{d}{dz}\widetilde{\mathbb{F}}_{\boldsymbol{n}_0}(\boldsymbol{n},z)|_{z=1}$ (see Appendix E), reported also in Eq. (8), is given by

$$\mathfrak{F}_{\boldsymbol{n}_0\to\boldsymbol{n}} = \frac{\mathcal{F}_{\boldsymbol{n}_0\to\boldsymbol{n}}\left|\underline{\boldsymbol{\mathcal{H}}} - 1/\mathcal{F}_{\boldsymbol{n}_0\to\boldsymbol{n}}\underline{\boldsymbol{\mathcal{H}}}^{(1)}\right|}{\left|\underline{\boldsymbol{\mathcal{H}}} - \underline{\boldsymbol{\mathcal{H}}}^{(2)}\right|}, \tag{A2}$$

where

$$\underline{\boldsymbol{\mathcal{H}}}_{i,j} = \frac{\lambda_{\boldsymbol{v}_i,\boldsymbol{u}_i}}{\mathcal{R}_{\boldsymbol{u}_i}}\mathcal{F}_{\langle \boldsymbol{u}_j-\boldsymbol{v}_j\rangle\to\boldsymbol{u}_i} - \frac{\lambda_{\boldsymbol{u}_i,\boldsymbol{v}_i}}{\mathcal{R}_{\boldsymbol{v}_i}}\mathcal{F}_{\langle \boldsymbol{u}_j-\boldsymbol{v}_j\rangle\to\boldsymbol{v}_i} + \delta_{i,j}, \tag{A3}$$

$$\underline{\boldsymbol{\mathcal{H}}}^{(1)}_{i,j} = \left(\frac{\lambda_{\boldsymbol{v}_i,\boldsymbol{u}_i}}{\mathcal{R}_{\boldsymbol{u}_i}}\mathcal{F}_{\langle \boldsymbol{n}_0-\boldsymbol{n}\rangle\to\boldsymbol{u}_i} - \frac{\lambda_{\boldsymbol{u}_i,\boldsymbol{v}_i}}{\mathcal{R}_{\boldsymbol{v}_i}}\mathcal{F}_{\langle \boldsymbol{n}_0-\boldsymbol{n}\rangle\to\boldsymbol{v}_i}\right) \times \mathcal{F}_{\langle \boldsymbol{u}_j-\boldsymbol{v}_j\rangle\to\boldsymbol{n}}, \tag{A4}$$

$$\underline{\boldsymbol{\mathcal{H}}}^{(2)}_{i,j} = \left(\frac{\lambda_{\boldsymbol{v}_i,\boldsymbol{u}_i}}{\mathcal{R}_{\boldsymbol{u}_i}} - \frac{\lambda_{\boldsymbol{u}_i,\boldsymbol{v}_i}}{\mathcal{R}_{\boldsymbol{v}_i}}\right)\mathcal{F}_{\langle \boldsymbol{u}_j-\boldsymbol{v}_j\rangle\to\boldsymbol{n}}. \tag{A5}$$

If the homogeneous propagator is diffusive with no bias and if the heterogeneity parameters are symmetric, i.e., $\lambda_{\boldsymbol{v},\boldsymbol{u}} = \lambda_{\boldsymbol{u},\boldsymbol{v}}$, $\underline{\boldsymbol{\mathcal{H}}}^{(2)} = 0$ and Eq. (8) can be simplified further

$$\mathfrak{F}_{\boldsymbol{n}_0\to\boldsymbol{n}} = \mathcal{F}_{\boldsymbol{n}_0\to\boldsymbol{n}} - 1 + \frac{\left|\underline{\boldsymbol{\mathcal{H}}} - \underline{\boldsymbol{\mathcal{H}}}^{(1)}\right|}{|\underline{\boldsymbol{\mathcal{H}}}|}. \tag{A6}$$

On the other hand, when one is only dealing with *only* sticky or slippery heterogeneities the elements of the matrices $\underline{\boldsymbol{\mathcal{H}}}$, $\underline{\boldsymbol{\mathcal{H}}}^{(1)}$, and $\underline{\boldsymbol{\mathcal{H}}}^{(2)}$ are given by Eqs. (E56) to (E58) and Eq. (E59).

1. Mean return time

Through the renewal equation, we also have the return probability relation

$$\widetilde{\mathbb{R}}(\boldsymbol{n},z) = 1 - \frac{1}{\widetilde{\Phi}_{\boldsymbol{n}}(\boldsymbol{n},z)} = \frac{(\widetilde{\varphi}_{\boldsymbol{n}}(\boldsymbol{n},z)-2)|\underline{\boldsymbol{H}}| + |\underline{\boldsymbol{H}}(\boldsymbol{n},\boldsymbol{n})|}{(\widetilde{\varphi}_{\boldsymbol{n}}(\boldsymbol{n},z)-1)|\underline{\boldsymbol{H}}| + |\underline{\boldsymbol{H}}(\boldsymbol{n},\boldsymbol{n})|}. \tag{A7}$$

By noticing the identical structures of Eqs. (A1) and (E60), one can use a similar procedure to the one used to derive the MFPT (see Appendix E 3) to show the mean return time (MRT) to be

$$\mathfrak{R}_{\boldsymbol{n}} = \frac{\mathcal{R}_{\boldsymbol{n}}|\underline{\boldsymbol{\mathcal{H}}}|}{\left|\underline{\boldsymbol{\mathcal{H}}} - \underline{\boldsymbol{\mathcal{H}}}^{(2)}\right|}. \tag{A8}$$

2. Mean exit times

The first-exit probability is given by $\widetilde{\mathbb{E}}_{\boldsymbol{n}_0}(z) = 1-(1-z)\widetilde{\mathbb{S}}_{\boldsymbol{n}_0}(z)$, where $\widetilde{\mathbb{S}}_{\boldsymbol{n}_0}(z)$ is the survival probability given by

$$\widetilde{\mathbb{S}}_{\boldsymbol{n}_0}(z) = \sum_{\boldsymbol{n}}\widetilde{\Phi}_{\boldsymbol{n}_0}(\boldsymbol{n},z). \tag{A9}$$

Substituting Eq. (5) into Eq. (A9) and evaluating the sum in $\boldsymbol{n}$ and simplifying the summation over k one finds

$$\widetilde{\mathbb{S}}_{\boldsymbol{n}_0}(z) = \widetilde{S}_{\boldsymbol{n}_0}(z) - 1 + \frac{|\underline{\boldsymbol{H}} - \underline{\boldsymbol{S}}(\boldsymbol{n}_0)|}{|\underline{\boldsymbol{H}}|} \tag{A10}$$

where $\widetilde{S}_{\boldsymbol{n}_0}(z) = \sum_{\boldsymbol{n}}\widetilde{\varphi}_{\boldsymbol{n}_0}(\boldsymbol{n},z)$ is the homogeneous survival probability, and where the elements of $\underline{\boldsymbol{H}}$ are given in Eq. (6) and

$$\underline{\boldsymbol{S}}(\boldsymbol{n}_0)_{i,j} = \widetilde{S}_{\langle \boldsymbol{u}_j-\boldsymbol{v}_j\rangle}(z)\left[\lambda_{\boldsymbol{v}_i,\boldsymbol{u}_i}\widetilde{\varphi}_{\boldsymbol{n}_0}(\boldsymbol{u}_i,z) - \lambda_{\boldsymbol{u}_i,\boldsymbol{v}_i}\widetilde{\varphi}_{\boldsymbol{n}_0}(\boldsymbol{v}_i,z)\right]. \tag{A11}$$

By taking the mean of the first-exit distribution, i.e., $\frac{d}{dz}\widetilde{\mathbb{E}}_{\boldsymbol{n}_0}(z)|_{z=1}$ gives Eq. (10). Simple expressions of the 1D problem are given in Appendix G.

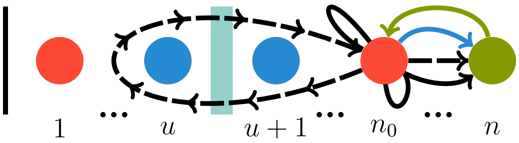

FIG. 16. A schematic representation of a one-dimensional heterogeneous lattice with a reflecting boundary to the left (vertical line) and with a permeable barrier between the sites u and $u+1$ represented by the shaded rectangle. The first-passage event can be split into mutually exclusive events represented by arrows of different colors. The blue arrows represent trajectories that never return to the initial site, while the black ones represent trajectories that return m times before reaching n. The green arrow represents first-passage trajectories that reach n_0 having starting at n. The solid arrows represent trajectories that are unaffected by the presence of the partially reflecting barrier between u and $u+1$, while the trajectories that are affected are represented by dashed arrows. Note that this schematic depicts the case when $1 \leqslant u \leqslant n_0 - 1$.

APPENDIX B: FIRST-PASSAGE QUANTITIES IN 1D SYSTEMS

1. The MFPT disorder indifference phenomenon

We start with a heterogeneous lattice reflecting boundary between $n=0$ and $n=1$, and a partially reflecting barrier between u and $u+1$, with $u < n_0 < n$ as depicted in Fig. 16. The trajectories that contribute to the first-passage probability can be split into mutually exclusive sets based on the number of return visits m to the initial site n_0.

We now formally represent the first-passage probability in terms of a set of mutually exclusive independent events. Let us define $F_{n_0}(n,t;m=0)$ as the first-passage probability to reach n for the first time at t having started at n_0 and having never returned to the initial site. Clearly, the trajectories that make up $F_{n_0}(n,t;m=0)$ (colored blue in Fig. 16), can never be affected by the presence of the barrier as they never move towards the barrier. The trajectories that *could* be affected by the presence of the barrier are those that return at-least once to the initial site before reaching n. The first-passage probability to visit n and having visited the initial site m times is constructed through the convolution (dashed trajectories in Fig. 16)

$$\mathbb{F}_{n_0}(n,t;m) = \sum_{t_1=0}^{t}\cdots\sum_{t_m=0}^{t_{m-1}} F_{n_0}(n,t-t_1;m=0) \times h_{n_0}(n,t_{m-1}-t_m)\cdots h_{n_0}(n,t_m), \tag{B1}$$

with $t_m \leqslant t_{m-1} \leqslant \cdots \leqslant t_1 \leqslant t$, and where

$$h_{n_0}(n,t) = \mathbb{R}(n_0,t) - \sum_{t'=0}^{t} F_{n_0}(n,t';m=0)F_n(n_0,t-t'). \tag{B2}$$

The function $h_{n_0}(n,t)$ represents the probability of returning without visiting the target and is constructed by considering the probability of returning to n_0 and subtracting those that reach n without returning to n_0 at some prior time and subsequently reaching n_0 from n. In z domain the relation can be written more conveniently as

$$\widetilde{\mathbb{F}}_{n_0}(n,z;m) = \widetilde{F}_{n_0}(n,z;m=0)[\widetilde{\mathbb{R}}(n_0,z) - \widetilde{F}_{n_0}(n,z;m=0)\widetilde{F}_n(n_0,z)]^m. \tag{B3}$$

Notice that the only term with the dependence on the barrier on the right-hand side (RHS) of Eq. (B3) is $\widetilde{\mathbb{R}}(n_0,z)$, and in the absence of the barrier $\widetilde{\mathbb{R}}(n_0,z)$ reduces to $\widetilde{R}(n_0,z)$. The full first-passage probability for the system with the barrier, which can be written as the sum of the mutually exclusive probabilities giving

$$\widetilde{\mathbb{F}}_{n_0}(n,z) = \widetilde{F}_{n_0}(n,z;m=0)\sum_{m=0}^{\infty}[\widetilde{\mathbb{R}}(n_0,z) - \widetilde{F}_{n_0}(n,z;m=0)\widetilde{F}_n(n_0,z)]^m. \tag{B4}$$

The relation given by Eq. (B4) is an alternative method of constructing the first-passage probability, i.e., it is not one of the standard approaches, which are through the survival probability or the ratio of propagators in z domain.

To confirm the normalization of the RHS of Eq. (B4) consider the following. By definition $F_{n_0}(n,t;m=0)$ is not normalized over t, hence, $\widetilde{F}_{n_0}(n,z=1;m=0)=p$ where $0<p<1$. Since all other terms in the RHS of Eq. (B4), namely, $\widetilde{F}_n(n_0,z)$ and $\widetilde{\mathbb{R}}(n_0,z)$ are normalized over time, we find that at $z=1$ the RHS becomes $\sum_{m=0}^{\infty} p(1-p)^m = 1$. Differentiating Eq. (B4) with respect to z and taking the limit $z\to 1$, we obtain the mean first-passage time

$$\mathfrak{F}_{n_0\to n} = \frac{\mathfrak{R}_n - p\mathcal{F}_{n\to n_0}}{p}. \tag{B5}$$

When the barrier is such that $\lambda_{u,u+1}=\lambda_{u+1,u}=\lambda$, the mean return time is equal to the reciprocal of the steady-state value, and $\mathfrak{R}_{n_0}$ becomes $\mathcal{R}_{n_0}$, i.e., the mean return time in the absence of the barrier.

To find p in Eq. (B5) explicitly, we first construct $\widetilde{F}_{n_0}(n,z;m=0)$ in terms of known quantities using the approach presented in Ref. [68] to construct time-dependent splitting probabilities. We write the two relations by considering the two splitting separately: the first-passage probability of reaching the target n and never returning to the initial condition $F_{n_0}(n,t;m=0)$; and the first-return probability to n_0 and having never reached the target site n. In time domain they are written via a convolution and are, respectively,

$$F_{n_0}(n,t;m=0) = F_{n_0}(n,t) - \sum_{t'=0}^{t}\mathbb{R}(n_0,t';n)F_{n_0}(n,t-t') \tag{B6}$$

and

$$\mathbb{R}(n_0,t;n) = \mathbb{R}(n_0,t) - \sum_{t'=0}^{t} F_{n_0}(n,t';m=0)F_n(n_0,t-t'), \tag{B7}$$

where $\mathbb{R}(n_0,t;n)$ is the probability of returning to the site n_0 at t and having never visited the target n. One can take the z transform and solve for $F_{n_0}(n,z;m=0)$ and $\mathbb{R}(n_0,t;n)$ giving, respectively,

$$\widetilde{F}_{n_0}(n,z;m=0) = \frac{\widetilde{F}_{n_0}(n,z) - \widetilde{\mathbb{R}}(n_0,z)\widetilde{F}_{n_0}(n,z)}{1-\widetilde{F}_{n_0}(n,z)\widetilde{F}_n(n_0,z)} \tag{B8}$$

and

$$\widetilde{\mathbb{R}}(n_0,z;n) = \frac{\widetilde{\mathbb{R}}(n_0,z) - \widetilde{F}_{n_0}(n,z)\widetilde{F}_n(n_0,z)}{1-\widetilde{F}_{n_0}(n,z)\widetilde{F}_n(n_0,z)}. \tag{B9}$$

Evaluating Eqs. (B8) and (B9) at $z=1$ gives, respectively, the fraction of all the first-passage trajectories that reach the target without returning to n_0 and the fraction of all trajectories that return to n_0 without ever reaching n. Using de L'Hôpital's rule once in Eq. (B8) we find

$$p = \frac{\mathfrak{R}_{n_0}}{\mathcal{F}_{n_0\to n} + \mathcal{F}_{n\to n_0}}. \tag{B10}$$

Inserting Eq. (B10) in Eq. (B5) one finds that $\mathfrak{F}_{n_0\to n} = \mathcal{F}_{n_0\to n}$.

2. MFPT linear dependence on disorder location

To understand the linear dependence in u, with $n_0 \leqslant u < n$, present in Eq. (12), we consider building up first-passage probability by convolution in time to go from n_0 to u first, then from u to $u+1$ and then from $u+1$ to n. In z domain one has

$$\widetilde{\mathbb{F}}_{n_0}(n,z) = \widetilde{F}_{n_0}(u,z)\widetilde{\mathbb{F}}_u(u+1,z)\widetilde{\mathbb{F}}_{u+1}(n,z), \tag{B11}$$

where the first term on the RHS has no dependence on the barrier as it is after the absorbing site u, while the other two terms are dependent on the barrier. Computing the mean of Eq. (B11), we obtain

$$\mathfrak{F}_{n_0\to n} = \mathcal{F}_{n_0\to u} + \mathfrak{F}_{u\to u+1} + \mathcal{F}_{u+1\to n}, \tag{B12}$$

where we have substituted $\mathfrak{F}_{u+1\to n} = \mathcal{F}_{u+1\to n}$ using the justification presented in the previous section. By using the relation $\mathcal{F}_{n_0\to n} = \mathcal{F}_{n_0\to s} + \mathcal{F}_{s\to n}$ with $n_0 < s < n$, one can rewrite Eq. (B12) to give

$$\mathfrak{F}_{n_0\to n} = \mathcal{F}_{n_0\to n} + \mathfrak{F}_{u\to u+1} - \mathcal{F}_{u\to u+1}. \tag{B13}$$

In the diffusive case, the MFPT to a neighboring site, $\mathcal{F}_{u\to u+1}$, is always proportional to twice the distance between u and the reflecting boundary to the left, i.e.,

$$\mathcal{F}_{u\to u+1} = \tfrac{2}{q}(u-s+1), \tag{B14}$$

with $s \leqslant u$ being the position of the reflecting boundary. By simplifying the general MFPT given in Eq. (8), we find

$$\mathfrak{F}_{u\to u+1} = \frac{2}{q-2\lambda}(u-s+1), \tag{B15}$$

which is analogous to Eq. (B14) but with the multiplicative (time rescale) factor increased to $2(q-2\lambda)^{-1}$. Letting $s=1$ we obtain Eq. (12).

APPENDIX C: PLACEMENT OF DEFECTS AND PARAMETER CHOICE OF THE MODELLING APPLICATIONS

1. Thigmotaxis

Two sets of defects must be placed, one set along a circle radius R to create a (circular) reflecting domain, while the second is used to divide this domain into two different regions (see Sec. VII) and is placed along a circle of radius r. To place defects on either circle one must first know which sites are within which circle. To determine this we use the Euclidean distance as a heuristic, with the site $\boldsymbol{n} = (n_1, n_2)$ being part of the circular domain if and only if $h(n_1, n_2) \leqslant R$, where $h(n_1, n_2) = [(n_1 - R - 1)^2 + (n_2 - R - 1)]^{1/2}$ with the size of the bounding square domain given by $\boldsymbol{N} = (2R + 1, 2R + 1)$. Similarly, a site is part of the inner region if and only if $h(n_1, n_2) \leqslant r$, while the outer region is given by $r < h(n_1, n_2) \leqslant R$. Given these site partitions, one can define two sets of defects, S_d and S_i describing, respectively, the impenetrable barriers to restrict the walker to a circular domain, and partially-reflecting inner barriers. In both cases $\boldsymbol{u}$ represents sites inside the circle of defects while $\boldsymbol{v}$ represents sites outside. For all $\{\boldsymbol{u}, \boldsymbol{v}\} \in S_i$ we have $\lambda_{\boldsymbol{v},\boldsymbol{u}} = \underline{\boldsymbol{A}}_{\boldsymbol{v},\boldsymbol{u}}$ and $\lambda_{\boldsymbol{u},\boldsymbol{v}}$ is irrelevant as the walker initially starts inside the circular domain. For all $\{\boldsymbol{u}, \boldsymbol{v}\} \in S_d$, we let $\lambda_{\boldsymbol{v},\boldsymbol{u}} = 0$ providing no resistance for the walker to enter the outer-region and $\lambda_{\boldsymbol{u},\boldsymbol{v}} = \alpha_i \underline{\boldsymbol{A}}_{\boldsymbol{u},\boldsymbol{v}}$ with $\alpha_i \in [0, 1]$.

2. Two-particle coalescing process

The interactions that need to modelled are binding and unbinding. Binding can occur via two distinct events. The first is when two particles are located on neighboring sites with $\boldsymbol{n} = (m + 1, m)$ and at the following time step, one of the particles remains at the same site while the second particle jumps onto the site occupied by the first resulting in $\boldsymbol{n} = (m + 1, m + 1)$ or $\boldsymbol{n} = (m, m)$. The second possible event occurs when the two particles are located two sites apart, i.e., $\boldsymbol{n} = (m - 1, m + 1)$ and at the following time step they both jump towards each other landing on $\boldsymbol{n} = (m, m)$. The reverse of these two events gives rise to unbinding of the complex $\boldsymbol{C}$. These transitions can be modified by placing paired defects of the forms: $\boldsymbol{u} = (m, m)$, $\boldsymbol{v} = (m + 1, m)$, and $\boldsymbol{u} = (m, m)$, $\boldsymbol{v} = (m, m + 1)$ for $1 \leqslant m \leqslant N - 1$ with $\lambda_{\boldsymbol{v},\boldsymbol{u}} = \frac{q}{2}(1 - q)(1 - \alpha_u)$, $\lambda_{\boldsymbol{u},\boldsymbol{v}} = \frac{q}{2}(1 - q)\alpha_e$; $\boldsymbol{u} = (m, m)$, $\boldsymbol{v} = (m, m - 1)$ and $\boldsymbol{u} = (m, m)$, $\boldsymbol{v} = (m - 1, m)$ for $2 \leqslant m \leqslant N$ with $\lambda_{\boldsymbol{v},\boldsymbol{u}} = \frac{q}{2}(1 - q)(1 - \alpha_u)$, $\lambda_{\boldsymbol{u},\boldsymbol{v}} = \frac{q}{2}(1 - q)\alpha_e$; $\boldsymbol{u} = (m, m)$, $\boldsymbol{v} = (m \mp 1, m \pm 1)$ for $2 \leqslant m \leqslant N - 1$ with $\lambda_{\boldsymbol{v},\boldsymbol{u}} = \frac{q^2}{4}(1 - \alpha_u)$, $\lambda_{\boldsymbol{u},\boldsymbol{v}} = \frac{q^2}{4}\alpha_e$.

Intuitively, the movement of the coalesced is slowed as it is more massive. To encode this detail we interpret jumps along the leading diagonal $\boldsymbol{n} = (m, m)$ for all $1 \leqslant m \leqslant N$ as the jumps made by the coalesced particle $\boldsymbol{C}$, and we slow its movement by placing paired defects of the form $\boldsymbol{u} = (m, m)$, $\boldsymbol{v} = (m + 1, m + 1)$ for all $1 \leqslant m \leqslant N - 1$ with $\lambda_{\boldsymbol{v},\boldsymbol{u}} = \lambda_{\boldsymbol{u},\boldsymbol{v}} = \frac{q^2}{4}(1 - \alpha_c)$.

APPENDIX D: DERIVATION OF THE HETEROGENEOUS PROPAGATOR

We consider a collection of heterogeneous connections as described in Sec. III, given by a set of M paired defects, $S = \{\{\boldsymbol{u}_1, \boldsymbol{v}_1\}, \dots, \{\boldsymbol{u}_M, \boldsymbol{v}_M\}\}$. When not on a defective site, that is when $\boldsymbol{n} \neq \boldsymbol{u}_k, \boldsymbol{v}_k$ for any k, the dynamics are given by

$$\Phi(\boldsymbol{n}, t + 1) = \sum_{\boldsymbol{m}} \underline{\boldsymbol{A}}_{\boldsymbol{n},\boldsymbol{m}} \Phi(\boldsymbol{m}, t), \quad \boldsymbol{n} \neq \boldsymbol{u}, \boldsymbol{v}, \ \forall \{\boldsymbol{u}, \boldsymbol{v}\} \in S, \tag{D1}$$

with $\underline{\boldsymbol{A}}_{\boldsymbol{n},\boldsymbol{m}}$ representing the transition probability from site $\boldsymbol{m}$ to site $\boldsymbol{n}$. When, instead, on any of the paired defective sites the dynamics are given by

$$\Phi(\boldsymbol{u}, t + 1) = \sum_{\boldsymbol{m}} \underline{\boldsymbol{A}}_{\boldsymbol{u},\boldsymbol{m}} \Phi(\boldsymbol{m}, t) + \lambda_{\boldsymbol{v},\boldsymbol{u}} \Phi(\boldsymbol{u}, t) - \lambda_{\boldsymbol{u},\boldsymbol{v}} \Phi(\boldsymbol{v}, t), \tag{D2}$$

and

$$\Phi(\boldsymbol{v}, t + 1) = \sum_{\boldsymbol{m}} \underline{\boldsymbol{A}}_{\boldsymbol{v},\boldsymbol{m}} \Phi(\boldsymbol{m}, t) - \lambda_{\boldsymbol{v},\boldsymbol{u}} \Phi(\boldsymbol{u}, t) + \lambda_{\boldsymbol{u},\boldsymbol{v}} \Phi(\boldsymbol{v}, t), \tag{D3}$$

where the bounds on $\lambda_{\boldsymbol{v},\boldsymbol{u}}$ and $\lambda_{\boldsymbol{u},\boldsymbol{v}}$ are given by Eqs. (2) and (3). Combining Eqs. (D1)–(D3) into a single equation and summing over all pairs in S gives the Master equation with defects

$$\Phi(\boldsymbol{n}, t + 1) = \sum_{\boldsymbol{m}} \underline{\boldsymbol{A}}_{\boldsymbol{n},\boldsymbol{m}} \Phi(\boldsymbol{n}, t) + \sum_{k=1}^{M} \delta_{\langle \boldsymbol{u}_k - \boldsymbol{v}_k \rangle, \boldsymbol{n}} \left[\lambda_{\boldsymbol{v}_k,\boldsymbol{u}_k} \Phi(\boldsymbol{u}_k, t) - \lambda_{\boldsymbol{u}_k,\boldsymbol{v}_k} \Phi(\boldsymbol{v}_k, t)\right], \tag{D4}$$

where $\delta_{\langle \boldsymbol{u}-\boldsymbol{v}\rangle,\boldsymbol{n}} = \delta_{\boldsymbol{u},\boldsymbol{n}} - \delta_{\boldsymbol{v},\boldsymbol{n}}$. Taking the z transform of Eq. (D4) we find

$$\widetilde{\Phi}(\boldsymbol{n}, z) - \Phi(\boldsymbol{n}, 0) = \sum_{\boldsymbol{m}} \underline{\boldsymbol{A}}_{\boldsymbol{n},\boldsymbol{m}} \widetilde{\Phi}(\boldsymbol{n}, z) + z \sum_{k=1}^{M} \delta_{\langle \boldsymbol{u}_k - \boldsymbol{v}_k \rangle, \boldsymbol{n}} \left[\lambda_{\boldsymbol{v}_k,\boldsymbol{u}_k} \widetilde{\Phi}(\boldsymbol{u}_k, z) - \lambda_{\boldsymbol{u}_k,\boldsymbol{v}_k} \widetilde{\Phi}(\boldsymbol{v}_k, z)\right]. \tag{D5}$$

Solving first the homogeneous difference equation, i.e., Eq. (D1) to get (in the absence of defects)

$$\widetilde{\Phi}(\boldsymbol{n}, z) = \sum_{\boldsymbol{m}} \widetilde{\varphi}_{\boldsymbol{m}}(\boldsymbol{n}, z) \Phi(\boldsymbol{m}, 0) \tag{D6}$$

where $\widetilde{\varphi}_{\boldsymbol{m}}(\boldsymbol{n}, z)$ is the propagator of the homogeneous problem {e.g., see Eq. (23) of Ref. [68] or Eq. (33) of Ref. [69]}, followed by a convolution (in time and space) with the inhomogeneous term in Eq. (D5) yields the formal solution

$$\widetilde{\Phi}(\boldsymbol{n}, z) = \sum_{\boldsymbol{m}} \widetilde{\varphi}_{\boldsymbol{m}}(\boldsymbol{n}, z) \Phi(\boldsymbol{m}, 0) + z \sum_{k=1}^{M} \widetilde{\varphi}_{\langle \boldsymbol{u}_k - \boldsymbol{v}_k \rangle}(\boldsymbol{n}, z) \left[\lambda_{\boldsymbol{v}_k,\boldsymbol{u}_k} \widetilde{\Phi}(\boldsymbol{u}_k, z) - \lambda_{\boldsymbol{u}_k,\boldsymbol{v}_k} \widetilde{\Phi}(\boldsymbol{v}_k, z)\right]. \tag{D7}$$

When the initial condition is localized, i.e., $\Phi(\boldsymbol{n}, 0) = \delta_{\boldsymbol{n},\boldsymbol{n}_0}$, we have the formal propagator

$$\widetilde{\Phi}_{\boldsymbol{n}_0}(\boldsymbol{n}, z) = \widetilde{\varphi}_{\boldsymbol{n}_0}(\boldsymbol{n}, z) + z \sum_{k=1}^{M} \widetilde{\varphi}_{\langle \boldsymbol{u}_k - \boldsymbol{v}_k \rangle}(\boldsymbol{n}, z) \times \left[\lambda_{\boldsymbol{v}_k,\boldsymbol{u}_k} \widetilde{\Phi}_{\boldsymbol{n}_0}(\boldsymbol{u}_k, z) - \lambda_{\boldsymbol{u}_k,\boldsymbol{v}_k} \widetilde{\Phi}_{\boldsymbol{n}_0}(\boldsymbol{v}_k, z)\right]. \tag{D8}$$

In order to find $\widetilde{\Phi}_{\boldsymbol{n}_0}(\boldsymbol{n}, z)$ in terms of the known propagator $\widetilde{\varphi}_{\boldsymbol{n}_0}(\boldsymbol{n}, z)$ we first create simultaneous equations for each

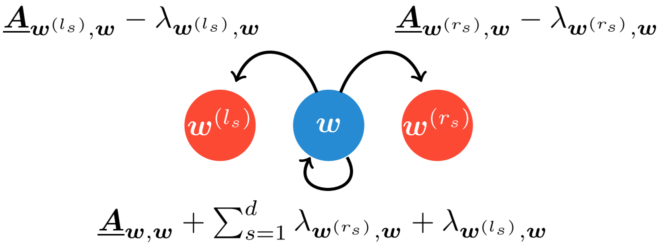

FIG. 17. A schematic representation showing the modified transition probabilities of a sticky (or slippery) heterogeneity. We highlight only the dynamics in the sth dimension, but the same is present for the other dimensions.

pair of defects in terms of the differences $\lambda_{\boldsymbol{v}_k,\boldsymbol{u}_k}\widetilde{\Phi}_{\boldsymbol{n}_0}(\boldsymbol{u}_k,z) - \lambda_{\boldsymbol{u}_k,\boldsymbol{v}_k}\widetilde{\Phi}_{\boldsymbol{n}_0}(\boldsymbol{v}_k,z)$ giving

$$\begin{aligned}\lambda_{\boldsymbol{v}_k,\boldsymbol{u}_k}\widetilde{\Phi}_{\boldsymbol{n}_0}(\boldsymbol{u}_k,z) &- \lambda_{\boldsymbol{u}_k,\boldsymbol{v}_k}\widetilde{\Phi}_{\boldsymbol{n}_0}(\boldsymbol{v}_k,z)\\ &= \lambda_{\boldsymbol{v}_k,\boldsymbol{u}_k}\widetilde{\varphi}_{\boldsymbol{n}_0}(\boldsymbol{u}_k,z) - \lambda_{\boldsymbol{u}_k,\boldsymbol{v}_k}\widetilde{\varphi}_{\boldsymbol{n}_0}(\boldsymbol{v}_k,z)\\ &\quad + z\sum_{\ell=1}^{M}\left[\lambda_{\boldsymbol{v}_k,\boldsymbol{u}_k}\widetilde{\varphi}_{\langle \boldsymbol{u}_\ell-\boldsymbol{v}_\ell\rangle}(\boldsymbol{u}_k,z) - \lambda_{\boldsymbol{u}_k,\boldsymbol{v}_k}\widetilde{\varphi}_{\langle \boldsymbol{u}_\ell-\boldsymbol{v}_\ell\rangle}(\boldsymbol{v}_k,z)\right]\\ &\quad \times\left[\lambda_{\boldsymbol{v}_\ell,\boldsymbol{u}_\ell}\widetilde{\Phi}_{\boldsymbol{n}_0}(\boldsymbol{u}_\ell,z) - \lambda_{\boldsymbol{u}_\ell,\boldsymbol{v}_\ell}\widetilde{\Phi}_{\boldsymbol{n}_0}(\boldsymbol{v}_\ell,z)\right],\end{aligned} \tag{D9}$$

whose solution via Cramer's rule, is given by

$$\lambda_{\boldsymbol{v}_k,\boldsymbol{u}_k}\widetilde{\Phi}_{\boldsymbol{n}_0}(\boldsymbol{u}_k,z) - \lambda_{\boldsymbol{u}_k,\boldsymbol{v}_k}\widetilde{\Phi}_{\boldsymbol{n}_0}(\boldsymbol{v}_k z) = -\frac{1}{z}\frac{|\underline{\boldsymbol{Y}}|}{|\underline{\boldsymbol{H}}|}, \tag{D10}$$

with $\underline{\boldsymbol{H}}$ defined in Eq. (6) and where $\underline{\boldsymbol{Y}}$ is the same as $\underline{\boldsymbol{H}}$, but with the kth column replaced by

$$\begin{aligned}&\left[\lambda_{\boldsymbol{v}_1,\boldsymbol{u}_1}\widetilde{\varphi}_{\boldsymbol{n}_0}(\boldsymbol{u}_1,z) - \lambda_{\boldsymbol{u}_1,\boldsymbol{v}_1}\widetilde{\varphi}_{\boldsymbol{n}_0}(\boldsymbol{v}_1,z),\ldots,\lambda_{\boldsymbol{v}_M,\boldsymbol{u}_M}\widetilde{\varphi}_{\boldsymbol{n}_0}(\boldsymbol{u}_M,z)\right.\\ &\left.\quad - \lambda_{\boldsymbol{u}_M,\boldsymbol{v}_M}\widetilde{\varphi}_{\boldsymbol{n}_0}(\boldsymbol{v}_M,z)\right]^{\mathsf{T}}.\end{aligned} \tag{D11}$$

Using Eq. (D10), Eq. (D8) becomes

$$\widetilde{\Phi}_{\boldsymbol{n}_0}(\boldsymbol{n},z) = \widetilde{\varphi}_{\boldsymbol{n}_0}(\boldsymbol{n},z) - \sum_{k=1}^{M}\widetilde{\varphi}_{\langle \boldsymbol{u}_k-\boldsymbol{v}_k\rangle}(\boldsymbol{n},z)\frac{|\underline{\boldsymbol{Y}}|}{|\underline{\boldsymbol{H}}|}. \tag{D12}$$

The summation in Eq. (D12) can be carried out explicitly giving

$$\widetilde{\Phi}_{\boldsymbol{n}_0}(\boldsymbol{n},z) = \widetilde{\varphi}_{\boldsymbol{n}_0}(\boldsymbol{n},z) - 1 + \frac{|\underline{\boldsymbol{H}} - \underline{\boldsymbol{G}}(\boldsymbol{n},\boldsymbol{n}_0)|}{|\underline{\boldsymbol{H}}|}. \tag{D13}$$

where

$$\begin{aligned}\underline{\boldsymbol{G}}(\boldsymbol{n},\boldsymbol{n}_0)_{i,j} &= \widetilde{\varphi}_{\langle \boldsymbol{u}_i-\boldsymbol{v}_i\rangle}(\boldsymbol{n},z)\\ &\quad \times\left[\lambda_{\boldsymbol{v}_i,\boldsymbol{u}_i}\widetilde{\varphi}_{\boldsymbol{n}_0}(\boldsymbol{u}_i,z) - \lambda_{\boldsymbol{u}_i,\boldsymbol{v}_i}\widetilde{\varphi}_{\boldsymbol{n}_0}(\boldsymbol{v}_i,z)\right]\end{aligned} \tag{D14}$$

and calling $\underline{\boldsymbol{H}}(\boldsymbol{n},\boldsymbol{n}_0) = \underline{\boldsymbol{H}} - \underline{\boldsymbol{G}}(\boldsymbol{n},\boldsymbol{n}_0)$ gives the solution presented in Eq. (5).

1. Sticky and slippery heterogeneities

We start with set of defective sites $S' = \{\boldsymbol{w}_1,\ldots,\boldsymbol{w}_L\}$ and use the notation $\boldsymbol{w}_i^{(l_s)}$ and $\boldsymbol{w}_i^{(r_s)}$ representing, respectively, the left and right neighbors of $\boldsymbol{w}_i$ in the sth dimension, given $\boldsymbol{w} = (w_1,\ldots,w_d)$, $\boldsymbol{w}^{(r_s)} = (w_1,\ldots w_s+1,\ldots,w_d)$ and $\boldsymbol{w}^{(l_s)} = (w_1,\ldots w_s-1,\ldots,w_d)$, with d the lattice dimension. A schematic representation of the jump probabilities on a defective site, $\boldsymbol{w}$ is given in Fig. 17, from which it is clear that to ensure positive probabilities one must have $\lambda_{\boldsymbol{w}^{(r_s)},\boldsymbol{w}} \leqslant \underline{\boldsymbol{A}}_{\boldsymbol{w}^{(r_s)},\boldsymbol{w}}$ and $\lambda_{\boldsymbol{w}^{(l_s)},\boldsymbol{w}} \leqslant \underline{\boldsymbol{A}}_{\boldsymbol{w}^{(l_s)},\boldsymbol{w}}$ for all $s = 1,\ldots,d$, and $0 \leqslant \underline{\boldsymbol{A}}_{\boldsymbol{w},\boldsymbol{w}} + \sum_{s=1}^{d}\lambda_{\boldsymbol{w}^{(r_s)},\boldsymbol{w}} + \lambda_{\boldsymbol{w}^{(l_s)},\boldsymbol{w}}$ for all $\boldsymbol{w} \in S'$. These conditions are a recast of the one given by Eqs. (2) and (3).

The full dynamics is described by the Master equation

$$\begin{aligned}\Phi(\boldsymbol{n},t+1) &= \sum_{\boldsymbol{m}}\underline{\boldsymbol{A}}_{\boldsymbol{n},\boldsymbol{m}}\Phi(\boldsymbol{m},t) + \sum_{k=1}^{L}\Phi(\boldsymbol{w}_k,t)\\ &\quad \times\left\{\sum_{s=1}^{d}\lambda_{\boldsymbol{w}_k^{(r_s)},\boldsymbol{w}_k}\delta_{\langle \boldsymbol{w}_k-\boldsymbol{w}_k^{(r_s)}\rangle,\boldsymbol{n}} + \lambda_{\boldsymbol{w}_k^{(l_s)},\boldsymbol{w}_k}\delta_{\langle \boldsymbol{w}_k-\boldsymbol{w}_k^{(l_s)}\rangle,\boldsymbol{n}}\right\}.\end{aligned} \tag{D15}$$

Using a localized initial condition $\Phi(\boldsymbol{n},0) = \delta_{\boldsymbol{n},\boldsymbol{n}_0}$ and proceeding as before by solving the homogeneous dynamics and convolution (in time and space) with the nonhomogeneous part of Eq. (D15) gives the formal solution

$$\widetilde{\Phi}_{\boldsymbol{n}_0}(\boldsymbol{n},z) = \widetilde{\varphi}_{\boldsymbol{n}_0}(\boldsymbol{n},z) + z\sum_{k=1}^{M}\widetilde{\Phi}_{\boldsymbol{n}_0}(\boldsymbol{w}_k,z)\widetilde{Q}_{\boldsymbol{w}_k}(\boldsymbol{n},z), \tag{D16}$$

where

$$\begin{aligned}\widetilde{Q}_{\boldsymbol{w}}(\boldsymbol{n},z) &= \sum_{s=1}^{d}\lambda_{\boldsymbol{w}^{(r_s)},\boldsymbol{w}}\widetilde{\varphi}_{\langle \boldsymbol{w}-\boldsymbol{w}^{(r_s)}\rangle}(\boldsymbol{n},z)\\ &\quad + \lambda_{\boldsymbol{w}^{(l_s)},\boldsymbol{w}}\widetilde{\varphi}_{\langle \boldsymbol{w}-\boldsymbol{w}^{(l_s)}\rangle}(\boldsymbol{n},z).\end{aligned} \tag{D17}$$

This formal solution is as special case of Eq. (D8) where $M = 2Ld$ where each of the sites in S' bears two paired defects for each of d dimensions. However, by noticing that the incoming connections of the sticky sites are left unmodified, i.e., $\lambda_{\boldsymbol{w},\boldsymbol{w}^{(l_s)}} = \lambda_{\boldsymbol{w},\boldsymbol{w}^{(r_s)}} = 0$ one can to simplify Eq. (D8) to Eq. (D16) thereby reducing the number unknowns by a factor of $2d$.

To find the full solution we let $\boldsymbol{n} = \boldsymbol{w}_k$ and solve the simultaneous equations

$$\widetilde{\Phi}_{\boldsymbol{n}_0}(\boldsymbol{w}_k,z) = \widetilde{\varphi}_{\boldsymbol{n}_0}(\boldsymbol{n},z) + z\sum_{\ell=1}^{M}\widetilde{\Phi}_{\boldsymbol{n}_0}(\boldsymbol{w}_\ell,z)\widetilde{Q}_{\boldsymbol{w}_\ell}(\boldsymbol{w}_k,z), \tag{D18}$$

with $k = 1,\ldots,M$ to get

$$\widetilde{\Phi}_{\boldsymbol{n}_0}(\boldsymbol{w}_k,z) = -\frac{1}{z}\frac{|\underline{\boldsymbol{Y}}|}{|\underline{\boldsymbol{H}}|} \tag{D19}$$

where, in this case, the matrix $\underline{\boldsymbol{H}}$ is simplified to

$$\underline{\boldsymbol{H}}_{i,j} = \widetilde{Q}_{\boldsymbol{w}_j}(\boldsymbol{w}_i,z) - \frac{1}{z}\delta_{i,j} \tag{D20}$$

and $\underline{\boldsymbol{Y}}$ is the same as $\underline{\boldsymbol{H}}$ but with the kth column replaced by $[\widetilde{\varphi}_{\boldsymbol{n}_0}(\boldsymbol{w}_1,z),\ldots,\widetilde{\varphi}_{\boldsymbol{n}_0}(\boldsymbol{w}_M,z)]^{\mathsf{T}}$. Substituting Eq. (D19) into Eq. (D16) and summing over k gives the full solution in Eq. (5) where

$$\underline{\boldsymbol{H}}(\boldsymbol{n},\boldsymbol{n}_0)_{i,j} = \underline{\boldsymbol{H}}_{i,j} - \widetilde{Q}_{\boldsymbol{w}_j}(\boldsymbol{n},z)\widetilde{\varphi}_{\boldsymbol{n}_0}(\boldsymbol{w}_i,z). \tag{D21}$$

APPENDIX E: DERIVATIONS OF FIRST-PASSAGE STATISTICS IN THE PRESENCE OF HETEROGENEITIES

1. Mean first-passage time with arbitrary type and number of heterogeneities

From the renewal equation, the generating function of the first-passage probability from $\boldsymbol{n}_0$ to $\boldsymbol{n}$ ($\boldsymbol{n} \neq \boldsymbol{n}_0$) is given by

$$\widetilde{\mathbb{F}}_{\boldsymbol{n}_0}(\boldsymbol{n}, z) = \frac{\widetilde{\Phi}_{\boldsymbol{n}_0}(\boldsymbol{n}, z)}{\widetilde{\Phi}_{\boldsymbol{n}}(\boldsymbol{n}, z)} = \frac{(\widetilde{\varphi}_{\boldsymbol{n}_0}(\boldsymbol{n}, z) - 1)|\underline{\boldsymbol{H}}| + |\underline{\boldsymbol{H}} - \underline{\boldsymbol{G}}(\boldsymbol{n}, \boldsymbol{n}_0)|}{(\widetilde{\varphi}_{\boldsymbol{n}}(\boldsymbol{n}, z) - 1)|\underline{\boldsymbol{H}}| + |\underline{\boldsymbol{H}} - \underline{\boldsymbol{G}}(\boldsymbol{n}, \boldsymbol{n})|}, \tag{E1}$$

where we have called $\underline{\boldsymbol{H}}(\boldsymbol{n}, \boldsymbol{n}_0) = \underline{\boldsymbol{H}} - \underline{\boldsymbol{G}}(\boldsymbol{n}, \boldsymbol{n}_0)$ with $\underline{\boldsymbol{H}}$ and $\underline{\boldsymbol{H}}(\boldsymbol{n}, \boldsymbol{n}_0)$ defined, respectively, in Eqs. (6) and (7). Note that the matrix $\underline{\boldsymbol{G}}(\boldsymbol{n}, \boldsymbol{m})$ can be written in the form $\boldsymbol{a}\boldsymbol{b}^{\mathsf{T}}$, where $\boldsymbol{a}$ and $\boldsymbol{b}$ are column vectors with elements $\boldsymbol{a}_i = \lambda_{\boldsymbol{v}_i,\boldsymbol{u}_i}\widetilde{\varphi}_{\boldsymbol{m}}(\boldsymbol{u}_i, z) - \lambda_{\boldsymbol{u}_i,\boldsymbol{v}_i}\widetilde{\varphi}_{\boldsymbol{m}}(\boldsymbol{v}_i, z)$ and $\boldsymbol{b}_i = \widetilde{\varphi}_{\langle \boldsymbol{u}_i - \boldsymbol{v}_i\rangle}(\boldsymbol{n}, z)$. We will exploit this property in the coming steps. Dividing both the numerator and denominator of Eq. (E1) by $\widetilde{\varphi}_{\boldsymbol{n}}(\boldsymbol{n}, z)$ gives

$$\widetilde{\mathbb{F}}_{\boldsymbol{n}_0}(\boldsymbol{n}, z) = \frac{(\widetilde{F}_{\boldsymbol{n}_0}(\boldsymbol{n}, z) - 1/\widetilde{\varphi}_{\boldsymbol{n}}(\boldsymbol{n}, z))|\underline{\boldsymbol{H}}| + 1/\widetilde{\varphi}_{\boldsymbol{n}}(\boldsymbol{n}, z)|\underline{\boldsymbol{H}} - \underline{\boldsymbol{G}}(\boldsymbol{n}, \boldsymbol{n}_0)|}{(1 - 1/\widetilde{\varphi}_{\boldsymbol{n}}(\boldsymbol{n}, z))|\underline{\boldsymbol{H}}| + 1/\widetilde{\varphi}_{\boldsymbol{n}}(\boldsymbol{n}, z)|\underline{\boldsymbol{H}} - \underline{\boldsymbol{G}}(\boldsymbol{n}, \boldsymbol{n})|}. \tag{E2}$$

Using the property

$$\alpha|\underline{\boldsymbol{A}} - \underline{\boldsymbol{B}}| = |\underline{\boldsymbol{A}} - \alpha\underline{\boldsymbol{B}}| - (1 - \alpha)|\underline{\boldsymbol{A}}| \tag{E3}$$

when α is a scalar and $\underline{\boldsymbol{B}} = \boldsymbol{a}\boldsymbol{b}^{\mathsf{T}}$ with $\boldsymbol{a}$ and $\boldsymbol{b}$ column vectors of appropriate size, we rewrite

$$\widetilde{\mathbb{F}}_{\boldsymbol{n}_0}(\boldsymbol{n}, z) = \frac{(\widetilde{F}_{\boldsymbol{n}_0}(\boldsymbol{n}, z) - 1)|\underline{\boldsymbol{H}}| + |\underline{\boldsymbol{H}} - 1/\widetilde{\varphi}_{\boldsymbol{n}}(\boldsymbol{n}, z)\underline{\boldsymbol{G}}(\boldsymbol{n}, \boldsymbol{n}_0)|}{|\underline{\boldsymbol{H}} - 1/\widetilde{\varphi}_{\boldsymbol{n}}(\boldsymbol{n}, z)\underline{\boldsymbol{G}}(\boldsymbol{n}, \boldsymbol{n})|}. \tag{E4}$$

Dividing through by $\prod_{k=1}^{M} \widetilde{\varphi}_{\boldsymbol{u}_k}(\boldsymbol{u}_k, z)\widetilde{\varphi}_{\boldsymbol{v}_k}(\boldsymbol{v}_k, z)$, where M is the total number of paired defects one finds

$$\widetilde{\mathbb{F}}_{\boldsymbol{n}_0}(\boldsymbol{n}, z) = \frac{\big(\widetilde{F}_{\boldsymbol{n}_0}(\boldsymbol{n}, z) - 1\big)|\underline{\boldsymbol{J}}| + |\underline{\boldsymbol{J}}(\boldsymbol{n}, \boldsymbol{n}_0)|}{|\underline{\boldsymbol{J}}(\boldsymbol{n}, \boldsymbol{n})|}. \tag{E5}$$

where the elements of the matrices $\underline{\boldsymbol{J}}$, $\underline{\boldsymbol{J}}(\boldsymbol{n}, \boldsymbol{n}_0)$ and $\underline{\boldsymbol{J}}(\boldsymbol{n}, \boldsymbol{n})$ are given in terms of first-passage and return probabilities

$$\underline{\boldsymbol{J}}_{i,j} = \lambda_{\boldsymbol{v}_i,\boldsymbol{u}_i}[1 - \widetilde{R}(\boldsymbol{v}_i, z)]\widetilde{F}_{\langle \boldsymbol{u}_j - \boldsymbol{v}_j\rangle}(\boldsymbol{u}_i, z) - \lambda_{\boldsymbol{u}_i,\boldsymbol{v}_i}[1 - \widetilde{R}(\boldsymbol{u}_i, z)]\widetilde{F}_{\langle \boldsymbol{u}_j - \boldsymbol{v}_j\rangle}(\boldsymbol{v}_i, z) - \delta_{i,j}z^{-1}[1 - \widetilde{R}(\boldsymbol{v}_i, z)][1 - \widetilde{R}(\boldsymbol{u}_i, z)], \tag{E6}$$

$$\underline{\boldsymbol{J}}(\boldsymbol{n}, \boldsymbol{n}_0)_{i,j} = \underline{\boldsymbol{J}}_{i,j} - \widetilde{F}_{\langle \boldsymbol{u}_j - \boldsymbol{v}_j\rangle}(\boldsymbol{n}, z)\big\{\lambda_{\boldsymbol{v}_i,\boldsymbol{u}_i}[1 - \widetilde{R}(\boldsymbol{v}_i, z)]\widetilde{F}_{\boldsymbol{n}_0}(\boldsymbol{u}_i, z) - \lambda_{\boldsymbol{u}_i,\boldsymbol{v}_i}[1 - \widetilde{R}(\boldsymbol{u}_i, z)]\widetilde{F}_{\boldsymbol{n}_0}(\boldsymbol{v}_i, z)\big\}, \tag{E7}$$

$$\underline{\boldsymbol{J}}(\boldsymbol{n}, \boldsymbol{n})_{i,j} = \underline{\boldsymbol{J}}_{i,j} - \widetilde{F}_{\langle \boldsymbol{u}_j - \boldsymbol{v}_j\rangle}(\boldsymbol{n}, z)\big\{\lambda_{\boldsymbol{v}_i,\boldsymbol{u}_i}[1 - \widetilde{R}(\boldsymbol{v}_i, z)]\widetilde{F}_{\boldsymbol{n}}(\boldsymbol{u}_i, z) - \lambda_{\boldsymbol{u}_i,\boldsymbol{v}_i}[1 - \widetilde{R}(\boldsymbol{u}_i, z)]\widetilde{F}_{\boldsymbol{n}}(\boldsymbol{v}_i, z)\big\}. \tag{E8}$$

The mean first-passage time is then given by

$$\mathcal{D}_z \cdot \widetilde{\mathbb{F}}_{\boldsymbol{n}_0}(\boldsymbol{n}, z)|_{z=1} = \left.\frac{(\mathcal{D}_z \cdot \mathbb{N})\mathbb{D} - (\mathcal{D}_z \cdot \mathbb{D})\mathbb{N}}{\mathbb{D}^2}\right|_{z\to 1}, \tag{E9}$$

where $\mathcal{D}_z^k \cdot f$ is the kth derivative of f with respect to z, $\mathbb{N} = (\widetilde{F}_{\boldsymbol{n}_0}(\boldsymbol{n}, z) - 1)|\underline{\boldsymbol{J}}| + |\underline{\boldsymbol{J}}(\boldsymbol{n}, \boldsymbol{n}_0)|$ and $\mathbb{D} = |\underline{\boldsymbol{J}}(\boldsymbol{n}, \boldsymbol{n})|$. When $z \to 1$, $|\underline{\boldsymbol{J}}|$, $|\underline{\boldsymbol{J}}(\boldsymbol{n}, \boldsymbol{n}_0)|$ and $|\underline{\boldsymbol{J}}(\boldsymbol{n}, \boldsymbol{n})|$ all reduce to zero and it becomes necessary to use de L'Hôpital's rule. In order to proceed, it helps to consider the kth derivative of a determinant of a matrix with size $M \times M$ given by

$$\mathcal{D}_z^k \cdot |\underline{\boldsymbol{A}}| = \sum_{k_1+\cdots+k_M=k} \frac{k!}{k_1!\cdots k_M!}\begin{vmatrix} \mathcal{D}_z^{k_1} \cdot \underline{\boldsymbol{A}}_{1,1} & \mathcal{D}_z^{k_2} \cdot \underline{\boldsymbol{A}}_{1,2} & \cdots & \mathcal{D}_z^{k_M} \cdot \underline{\boldsymbol{A}}_{1,M} \\ \mathcal{D}_z^{k_1} \cdot \underline{\boldsymbol{A}}_{1,2} & \mathcal{D}_z^{k_2} \cdot \underline{\boldsymbol{A}}_{2,2} & \cdots & \mathcal{D}_z^{k_M} \cdot \underline{\boldsymbol{A}}_{2,M} \\ \vdots & \vdots & \ddots & \vdots \\ \mathcal{D}_z^{k_1} \cdot \underline{\boldsymbol{A}}_{M,1} & \mathcal{D}_z^{k_2} \cdot \underline{\boldsymbol{A}}_{i,j} & \cdots & \mathcal{D}_z^{k_M} \cdot \underline{\boldsymbol{A}}_{M,M} \end{vmatrix}. \tag{E10}$$

From Eq. (E10) and the expressions in Eqs. (E6)–(E8) it becomes clear that each of the columns in the matrices must be differentiated at-least twice to give a nonzero determinant when $z \to 1$. The determinant must therefore be differentiated $2M$ times leading to de L'Hôpital's rule being used $4M$-times in Eq. (E9). Expanding the denominator using Leibniz general rule gives

$$\mathcal{D}_z^{4M} \cdot \mathbb{D}^2 = \sum_{k=0}^{4M} \binom{4M}{k}(\mathcal{D}_z^k \cdot \mathbb{D})(\mathcal{D}_z^{4M-k} \cdot \mathbb{D}) = \binom{4M}{2M}(\mathcal{D}_z^{2M} \cdot \mathbb{D})^2, \tag{E11}$$

where the only nonzero term is when $k = 2M$, expanding the first term in the numerator in Eq. (E9) yields

$$\mathcal{D}_z^{4M} \cdot [(\mathcal{D}_z \cdot \mathbb{N})\mathbb{D}] = \sum_{k=0}^{4M} \binom{4M}{k} \big(\mathcal{D}_z^{k+1} \cdot \mathbb{N}\big)\big(\mathcal{D}_z^{4M-k} \cdot \mathbb{D}\big) = \binom{4M}{2M-1} \big(\mathcal{D}_z^{2M} \cdot \mathbb{N}\big)\big(\mathcal{D}_z^{2M+1} \cdot \mathbb{D}\big) + \binom{4M}{2M} \big(\mathcal{D}_z^{2M+1} \cdot \mathbb{N}\big)\big(\mathcal{D}_z^{2M} \cdot \mathbb{D}\big), \tag{E12}$$

where the only surviving terms of the above summation are when $k = 2M - 1$, and $k = 2M$. Similarly for the second term in the numerator in Eq. (E9) the surviving terms are obtained when $k = 2M$ and $k = 2M + 1$, giving

$$\mathcal{D}_z^{4M} \cdot [(\mathcal{D}_z \cdot \mathbb{D})\mathbb{N}] = \sum_{k=0}^{4M} \binom{4M}{k} \big(\mathcal{D}_z^{k} \cdot \mathbb{N}\big)\big(\mathcal{D}_z^{4M-k+1} \cdot \mathbb{D}\big) = \binom{4M}{2M} \big(\mathcal{D}_z^{2M} \cdot \mathbb{N}\big)\big(\mathcal{D}_z^{2M+1} \cdot \mathbb{D}\big) + \binom{4M}{2M+1} \big(\mathcal{D}_z^{2M+1} \cdot \mathbb{N}\big)\big(\mathcal{D}_z^{2M} \cdot \mathbb{D}\big). \tag{E13}$$

Putting it all together gives

$$\mathcal{D}_z \cdot \widetilde{\mathbb{F}}_{\boldsymbol{n}_0}(\boldsymbol{n}, z)|_{z=1} = \frac{\big(\mathcal{D}_z^{2M+1} \cdot \mathbb{N}\big)\big(\mathcal{D}_z^{2M} \cdot \mathbb{D}\big) - \big(\mathcal{D}_z^{2M} \cdot \mathbb{N}\big)\big(\mathcal{D}_z^{2M+1} \cdot \mathbb{D}\big)}{(2M+1)\big[\mathcal{D}_z^{2M} \cdot \mathbb{D}\big]^2}\Bigg|_{z\to 1}. \tag{E14}$$

Considering the term $\mathcal{D}_z^{2M} \cdot \mathbb{N}$ we find that

$$\mathcal{D}_z^{2M} \cdot \mathbb{N}|_{z\to 1} = \mathcal{D}_z^{2M} \cdot \big[\big(\widetilde{F}_{\boldsymbol{n}_0}(\boldsymbol{n}, z) - 1\big)|\underline{\boldsymbol{J}}|\big]|_{z\to 1} + \mathcal{D}_z^{2M} \cdot |\underline{\boldsymbol{J}}(\boldsymbol{n}, \boldsymbol{n}_0)||_{z\to 1} \tag{E15}$$

$$= \mathcal{D}_z^{2M} \cdot |\underline{\boldsymbol{J}}(\boldsymbol{n}, \boldsymbol{n}_0)||_{z\to 1}, \tag{E16}$$

since $[\widetilde{F}_{\boldsymbol{n}_0}(\boldsymbol{n}, z) - 1]$ must be differentiated at least once and $|\underline{\boldsymbol{J}}|$ must be differentiated at least $2M$ times to give a nonzero contribution. From Eqs. (E7) and (E8) we observe that a nonzero contribution from $\underline{\boldsymbol{J}}(\boldsymbol{n}, \boldsymbol{n}_0)_{i,j}$, $\underline{\boldsymbol{J}}(\boldsymbol{n}, \boldsymbol{n})_{i,j}$ occurs when one differentiates the difference of first-passage probability $\widetilde{F}_{\langle \boldsymbol{u}_j - \boldsymbol{v}_j\rangle}(\boldsymbol{n}, z)$, and the return probability terms $[1 - \widetilde{R}(\boldsymbol{u}_i, z)]$ and $[1 - \widetilde{R}(\boldsymbol{v}_i, z)]$ at least once. As $\widetilde{F}_{\boldsymbol{n}_0}(\boldsymbol{u}_i, z = 1) = \widetilde{F}_{\boldsymbol{n}_0}(\boldsymbol{v}_i, z = 1) = \widetilde{F}_{\boldsymbol{n}}(\boldsymbol{u}_i, z = 1) = \widetilde{F}_{\boldsymbol{n}}(\boldsymbol{v}_i, z = 1) = 1$, we have

$$\mathcal{D}_z^{2M} \cdot \mathbb{N}|_{z\to 1} = \mathcal{D}_z^{2M} \cdot \underline{\boldsymbol{J}}(\boldsymbol{n}, \boldsymbol{n}_0)|_{z\to 1} = \mathcal{D}_z^{2M} \cdot \underline{\boldsymbol{J}}(\boldsymbol{n}, \boldsymbol{n})|_{z\to 1} = \mathcal{D}_z^{2M} \cdot \mathbb{D}|_{z\to 1}, \tag{E17}$$

and we can simplify Eq. (E14) to

$$\mathcal{D}_z \cdot \widetilde{\mathbb{F}}_{\boldsymbol{n}_0}(\boldsymbol{n}, z)|_{z=1} = \frac{\mathcal{D}_z^{2M+1} \cdot \mathbb{N} - \mathcal{D}_z^{2M+1} \cdot \mathbb{D}}{(2M+1)\mathcal{D}_z^{2M} \cdot \mathbb{D}}\Bigg|_{z\to 1}. \tag{E18}$$

At this stage one can compute the derivatives explicitly, for the case $\mathcal{D}_z^{2M} \cdot \underline{\boldsymbol{J}}$ in Eq. (E10). With all $k_i = 2$, differentiating each column twice and taking the limit $z \to 1$, gives (after canceling the 2^M term),

$$\mathcal{D}_z^{2M} \cdot \underline{\boldsymbol{J}}|_{z\to 1} = (-1)^M (2M)!|\underline{\boldsymbol{\mathcal{J}}}|, \tag{E19}$$

where

$$\underline{\boldsymbol{\mathcal{J}}}_{i,j} = \lambda_{\boldsymbol{v}_i,\boldsymbol{u}_i} \mathcal{R}_{\boldsymbol{v}_i} \mathcal{F}_{\langle \boldsymbol{u}_j - \boldsymbol{v}_j\rangle \to \boldsymbol{u}_i} - \lambda_{\boldsymbol{u}_i,\boldsymbol{v}_i} \mathcal{R}_{\boldsymbol{u}_i} \mathcal{F}_{\langle \boldsymbol{u}_j - \boldsymbol{v}_j\rangle \to \boldsymbol{v}_i} + \delta_{i,j} \mathcal{R}_{\boldsymbol{u}_i} \mathcal{R}_{\boldsymbol{v}_i}, \tag{E20}$$

and likewise for the other two matrices we find

$$\mathcal{D}_z^{2M} \cdot \underline{\boldsymbol{J}}(\boldsymbol{n}, \boldsymbol{n})|_{z\to 1} = \mathcal{D}_z^{2M} \cdot \underline{\boldsymbol{J}}(\boldsymbol{n}, \boldsymbol{n}_0)|_{z\to 1} = (-1)^M (2M)!|\underline{\boldsymbol{\mathcal{J}}} - \underline{\boldsymbol{\mathcal{J}}}^{(2)}|, \tag{E21}$$

where

$$\underline{\boldsymbol{\mathcal{J}}}^{(2)}_{i,j} = (\lambda_{\boldsymbol{v}_i,\boldsymbol{u}_i} \mathcal{R}_{\boldsymbol{v}_i} - \lambda_{\boldsymbol{u}_i,\boldsymbol{v}_i} \mathcal{R}_{\boldsymbol{u}_i}) \mathcal{F}_{\langle \boldsymbol{u}_j - \boldsymbol{v}_j\rangle \to \boldsymbol{n}}. \tag{E22}$$

Let us now consider the term $\mathcal{D}_z^{2M+1} \cdot \mathbb{N}$. We apply Leibniz rule and obtain

$$\mathcal{D}_z^{2M+1} \cdot \mathbb{N} = \sum_{\ell=0}^{2M+1} \binom{2M+1}{\ell} \big[\mathcal{D}_z^{\ell} \cdot \big(\widetilde{F}_{\boldsymbol{n}_0}(\boldsymbol{n}, z) - 1\big)\big]\big[\mathcal{D}_z^{2M+1-\ell} \cdot |\underline{\boldsymbol{J}}|\big] + \mathcal{D}_z^{2M+1} \cdot |\underline{\boldsymbol{J}}(\boldsymbol{n}, \boldsymbol{n}_0)|, \tag{E23}$$

the only surviving term in the summation is when $\ell = 1$ resulting in

$$\mathcal{D}_z^{2M+1} \cdot \mathbb{N} = (-1)^M (2M+1)! \mathcal{F}_{\boldsymbol{n}_0 \to \boldsymbol{n}} \big|\underline{\boldsymbol{\mathcal{J}}}\big| + \mathcal{D}_z^{2M+1} \cdot \big|\underline{\boldsymbol{J}}(\boldsymbol{n}, \boldsymbol{n}_0)\big|. \tag{E24}$$

Substituting the previous results into Eq. (E14) and simplifying yields

$$\mathcal{D}_z \cdot \widetilde{\mathbb{F}}_{\boldsymbol{n}_0}(\boldsymbol{n}, z)|_{z=1} = \frac{(-1)^M (2M+1)! \mathcal{F}_{\boldsymbol{n}_0 \to \boldsymbol{n}} |\underline{\boldsymbol{\mathcal{J}}}| + \mathcal{D}_z^{2M+1} \cdot |\underline{\boldsymbol{J}}(\boldsymbol{n}, \boldsymbol{n}_0)| - \mathcal{D}_z^{2M+1} \cdot |\underline{\boldsymbol{J}}(\boldsymbol{n}, \boldsymbol{n})|}{(-1)^M (2M+1)! |\underline{\boldsymbol{\mathcal{J}}} - \underline{\boldsymbol{\mathcal{J}}}^{(2)}|}\Bigg|_{z\to 1}. \tag{E25}$$

Consider the term $\mathcal{D}_z^{2M+1}\cdot|\underline{\boldsymbol{J}}(\boldsymbol{n},\boldsymbol{n}_0)|$ and $\mathcal{D}_z^{2M+1}\cdot|\underline{\boldsymbol{J}}(\boldsymbol{n},\boldsymbol{n})|$, using the multinomial expansion of the derivative of a determinant, i.e., Eq. (E10), one can see that the only surviving terms appear when $M-1$ columns are differentiated twice, while one column is differentiated three times, hence,

$$\mathcal{D}_z^{2M+1}\cdot\{|\underline{\boldsymbol{J}}(\boldsymbol{n},\boldsymbol{n}_0)|-|\underline{\boldsymbol{J}}(\boldsymbol{n},\boldsymbol{n})|\}=\frac{1}{3}\frac{(2M+1)!}{2^M}\sum_{\ell=1}^{M}|\underline{\underline{\widehat{\mathcal{J}}}}^{(\boldsymbol{n}_0,\ell)}|-|\underline{\underline{\widehat{\mathcal{J}}}}^{(\boldsymbol{n},\ell)}|, \tag{E26}$$

where

$$\underline{\underline{\widehat{\mathcal{J}}}}^{(\boldsymbol{n}_0,\ell)}_{i,j}=2(\delta_{j,\ell}-1)\big\{\underline{\mathcal{J}}_{i,j}-\underline{\mathcal{J}}^{(2)}_{i,j}\big\}+\delta_{j,\ell}\big\{\mathcal{D}_z^3\cdot\underline{\boldsymbol{J}}(\boldsymbol{n},\boldsymbol{n}_0)_{i,j}|_{z=1}\big\}, \tag{E27}$$

$$\underline{\underline{\widehat{\mathcal{J}}}}^{(\boldsymbol{n},\ell)}_{i,j}=2(\delta_{j,\ell}-1)\big\{\underline{\mathcal{J}}_{i,j}-\underline{\mathcal{J}}^{(2)}_{i,j}\big\}+\delta_{j,\ell}\big\{\mathcal{D}_z^3\cdot\underline{\boldsymbol{J}}(\boldsymbol{n},\boldsymbol{n})_{i,j}|_{z=1}\big\}. \tag{E28}$$

The multilinear property of the determinant allows us to rewrite Eq. (E26) as

$$\mathcal{D}_z^{2M+1}\cdot\{|\underline{\boldsymbol{J}}(\boldsymbol{n},\boldsymbol{n}_0)|-|\underline{\boldsymbol{J}}(\boldsymbol{n},\boldsymbol{n})|\}=\frac{1}{3}\frac{(2M+1)!}{2^M}\sum_{\ell=1}^{M}|\underline{\underline{\widehat{\mathcal{J}}}}^{(\ell)}|, \tag{E29}$$

where

$$\underline{\underline{\widehat{\mathcal{J}}}}^{(\ell)}_{i,j}=2(\delta_{j,\ell}-1)\big\{\underline{\mathcal{J}}_{i,j}-\underline{\mathcal{J}}^{(2)}_{i,j}\big\}+\delta_{j,\ell}\mathcal{D}_z^3\cdot\{\underline{\boldsymbol{J}}(\boldsymbol{n},\boldsymbol{n}_0)_{i,j}-\underline{\boldsymbol{J}}(\boldsymbol{n},\boldsymbol{n})_{i,j}\}|_{z=1}. \tag{E30}$$

Since

$$\underline{\boldsymbol{J}}(\boldsymbol{n},\boldsymbol{n}_0)_{i,j}-\underline{\boldsymbol{J}}(\boldsymbol{n},\boldsymbol{n})_{i,j}=\widetilde{F}_{\langle\boldsymbol{u}_j-\boldsymbol{v}_j\rangle}(\boldsymbol{n},z)\big\{\lambda_{\boldsymbol{v}_i,\boldsymbol{u}_i}[1-\widetilde{R}(\boldsymbol{v}_i,z)]\widetilde{F}_{\langle\boldsymbol{n}_0-\boldsymbol{n}\rangle}(\boldsymbol{u}_i,z)-\lambda_{\boldsymbol{u}_i,\boldsymbol{v}_i}[1-\widetilde{R}(\boldsymbol{u}_i,z)]\widetilde{F}_{\langle\boldsymbol{n}_0-\boldsymbol{n}\rangle}(\boldsymbol{v}_i,z)\big\}, \tag{E31}$$

each of the first-passage and return probabilities in Eq. (E31) must be differentiated at least once to give a nonzero contribution, hence,

$$\mathcal{D}_z^3\cdot\{\underline{\boldsymbol{J}}(\boldsymbol{n},\boldsymbol{n}_0)_{i,j}-\underline{\boldsymbol{J}}(\boldsymbol{n},\boldsymbol{n})_{i,j}\}|_{z=1}=6\mathcal{F}_{\langle\boldsymbol{u}_j-\boldsymbol{v}_j\rangle\to\boldsymbol{n}}\big\{\lambda_{\boldsymbol{v}_i,\boldsymbol{u}_i}\mathcal{R}_{\boldsymbol{v}_i}\mathcal{F}_{\langle\boldsymbol{n}_0-\boldsymbol{n}\rangle\to\boldsymbol{u}_i}-\lambda_{\boldsymbol{u}_i,\boldsymbol{v}_i}\mathcal{R}_{\boldsymbol{u}_i}\mathcal{F}_{\langle\boldsymbol{n}_0-\boldsymbol{n}\rangle\to\boldsymbol{v}_i}\big\}, \tag{E32}$$

where the factor 6 comes from repeated application of the product rule. To carry out the summation in Eq. (E29) we employ the following property of determinants. Given two matrices, $\underline{\boldsymbol{A}}$ and $\underline{\boldsymbol{B}}$ of size $M\times M$, where $\underline{\boldsymbol{B}}=\boldsymbol{a}\boldsymbol{b}^{\mathsf{T}}$ and where $\boldsymbol{a}$ and $\boldsymbol{b}$ are two column vectors, the following relation

$$\sum_{\ell=1}^{M}|\underline{\boldsymbol{A}}^{(\ell)}|=|\underline{\boldsymbol{A}}|-|\underline{\boldsymbol{A}}-\underline{\boldsymbol{B}}| \tag{E33}$$

holds when $\underline{\boldsymbol{A}}^{(\ell)}$ is the same as $\underline{\boldsymbol{A}}$, but with the ℓth column replaced by the ℓth column of $\underline{\boldsymbol{B}}$. Comparing Eqs. (E29) and (E32) with Eq. (E33), we observe that $\underline{\boldsymbol{A}}\to\underline{\mathcal{J}}-\underline{\mathcal{J}}^{(2)}$,

$$\boldsymbol{a}\to\big[\lambda_{\boldsymbol{v}_1,\boldsymbol{u}_1}\mathcal{R}_{\boldsymbol{v}_1}\mathcal{F}_{\langle\boldsymbol{n}_0-\boldsymbol{n}\rangle\to\boldsymbol{u}_1}-\lambda_{\boldsymbol{u}_1,\boldsymbol{v}_1}\mathcal{R}_{\boldsymbol{u}_1}\mathcal{F}_{\langle\boldsymbol{n}_0-\boldsymbol{n}\rangle\to\boldsymbol{u}_1},\cdots,\lambda_{\boldsymbol{v}_M,\boldsymbol{u}_M}\mathcal{R}_{\boldsymbol{v}_M}\mathcal{F}_{\langle\boldsymbol{n}_0-\boldsymbol{n}\rangle\to\boldsymbol{u}_M}-\lambda_{\boldsymbol{u}_M,\boldsymbol{v}_M}\mathcal{R}_{\boldsymbol{u}_M}\mathcal{F}_{\langle\boldsymbol{n}_0-\boldsymbol{n}\rangle\to\boldsymbol{u}_M}\big]^{\mathsf{T}}, \tag{E34}$$

and

$$\boldsymbol{b}\to 6\big[\mathcal{F}_{\langle\boldsymbol{u}_1-\boldsymbol{v}_1\rangle\to\boldsymbol{n}},\cdots,\mathcal{F}_{\langle\boldsymbol{u}_M-\boldsymbol{v}_M\rangle\to\boldsymbol{n}}\big]^{\mathsf{T}}, \tag{E35}$$

and carrying out the summation we obtain

$$\frac{1}{3}\frac{(2M+1)!}{2^M}\sum_{\ell=1}^{M}|\underline{\underline{\widehat{\mathcal{J}}}}^{(\ell)}|=\frac{1}{3}\frac{(2M+1)!}{2^M}\big\{|-2(\underline{\mathcal{J}}-\underline{\mathcal{J}}^{(2)})|-|-2(\underline{\mathcal{J}}-\underline{\mathcal{J}}^{(2)})-6\underline{\mathcal{J}}^{(1)}|\big\}, \tag{E36}$$

with

$$\underline{\mathcal{J}}^{(1)}_{i,j}=\big(\lambda_{\boldsymbol{v}_i,\boldsymbol{u}_i}\mathcal{R}_{\boldsymbol{v}_i}\mathcal{F}_{\langle\boldsymbol{n}_0-\boldsymbol{n}\rangle\to\boldsymbol{u}_i}-\lambda_{\boldsymbol{u}_i,\boldsymbol{v}_i}\mathcal{R}_{\boldsymbol{u}_i}\mathcal{F}_{\langle\boldsymbol{n}_0-\boldsymbol{n}\rangle\to\boldsymbol{v}_i}\big)\mathcal{F}_{\langle\boldsymbol{u}_j-\boldsymbol{v}_j\rangle\to\boldsymbol{n}}. \tag{E37}$$

From Eq. (E36), we can factor out (–2) from the determinants using the property $|\alpha\underline{\boldsymbol{A}}|=\alpha^M|\underline{\boldsymbol{A}}|$ with $\underline{\boldsymbol{A}}$ an $M\times M$ determinant to yield

$$\frac{1}{3}\frac{(2M+1)!}{2^M}\sum_{\ell=1}^{M}|\underline{\underline{\widehat{\mathcal{J}}}}^{(\ell)}|=\frac{(-1)^M(2M+1)!}{3}\big\{|(\underline{\mathcal{J}}-\underline{\mathcal{J}}^{(2)})|-|(\underline{\mathcal{J}}-\underline{\mathcal{J}}^{(2)})+3\underline{\mathcal{J}}^{(1)}|\big\}. \tag{E38}$$

The factor $1/3$ can be taken into the determinants using the property given in Eq. (E3). For the first determinant on the RHS of Eq. (E38) we can equate $\underline{\boldsymbol{A}}\to\underline{\mathcal{J}}$, from Eq. (E22) we can equate

$$\boldsymbol{a}\to\big[\lambda_{\boldsymbol{v}_1,\boldsymbol{u}_1}\mathcal{R}_{\boldsymbol{v}_1}-\lambda_{\boldsymbol{u}_1,\boldsymbol{v}_1}\mathcal{R}_{\boldsymbol{u}_1},\ldots,\lambda_{\boldsymbol{v}_M,\boldsymbol{u}_M}\mathcal{R}_{\boldsymbol{v}_M}-\lambda_{\boldsymbol{u}_1,\boldsymbol{v}_1}\mathcal{R}_{\boldsymbol{u}_M}\big]^{\mathsf{T}}, \tag{E39}$$

and

$$\boldsymbol{b} \to \left[\mathcal{F}_{\langle \boldsymbol{u}_1-\boldsymbol{v}_1\rangle\to \boldsymbol{n}}, \ldots, \mathcal{F}_{\langle \boldsymbol{u}_M-\boldsymbol{v}_M\rangle\to \boldsymbol{n}}\right]^{\mathsf{T}}, \tag{E40}$$

to derive the relation

$$\tfrac{1}{3}|\underline{\mathcal{J}} - \underline{\mathcal{J}}^{(2)}| = |\underline{\mathcal{J}} - 1/3\underline{\mathcal{J}}^{(2)}| - \tfrac{2}{3}|\underline{\mathcal{J}}|. \tag{E41}$$

Similarly, for the second determinant on the RHS of Eq. (E38), we can equate $\underline{\boldsymbol{A}} \to \underline{\boldsymbol{J}}$, and using Eq. (E37) we identify

$$\boldsymbol{a} \to \big[\lambda_{\boldsymbol{v}_1,\boldsymbol{u}_1}\mathcal{R}_{\boldsymbol{v}_1}\big(1 - 3\mathcal{F}_{\langle \boldsymbol{n}_0-\boldsymbol{n}\rangle\to \boldsymbol{u}_1}\big) - \lambda_{\boldsymbol{u}_1,\boldsymbol{v}_1}\mathcal{R}_{\boldsymbol{u}_1}\big(1 - 3\mathcal{F}_{\langle \boldsymbol{n}_0-\boldsymbol{n}\rangle\to \boldsymbol{v}_1}\big),$$
$$\cdots, \lambda_{\boldsymbol{v}_M,\boldsymbol{u}_M}\mathcal{R}_{\boldsymbol{v}_M}\big(1 - 3\mathcal{F}_{\langle \boldsymbol{n}_0-\boldsymbol{n}\rangle\to \boldsymbol{u}_M}\big) - \lambda_{\boldsymbol{u}_1,\boldsymbol{v}_1}\mathcal{R}_{\boldsymbol{u}_M}\big(1 - 3\mathcal{F}_{\langle \boldsymbol{n}_0-\boldsymbol{n}\rangle\to \boldsymbol{v}_M}\big)\big]^{\mathsf{T}}, \tag{E42}$$

and

$$\boldsymbol{b} \to \left[\mathcal{F}_{\langle \boldsymbol{u}_1-\boldsymbol{v}_1\rangle\to \boldsymbol{n}}, \cdots, \mathcal{F}_{\langle \boldsymbol{u}_M-\boldsymbol{v}_M\rangle\to \boldsymbol{n}}\right]^{\mathsf{T}}, \tag{E43}$$

to obtain the relation

$$\tfrac{1}{3}|\underline{\mathcal{J}} - \underline{\mathcal{J}}^{(2)} + 3\underline{\mathcal{J}}^{(1)}| = |\underline{\mathcal{J}} - 1/3\underline{\mathcal{J}}^{(2)} + \underline{\mathcal{J}}^{(1)}| - \tfrac{2}{3}|\underline{\mathcal{J}}|. \tag{E44}$$

Using the relations Eqs. (E41) and (E44) to simplify Eq. (E38) yields

$$\frac{1}{3}\frac{(2M+1)!}{2^M}\sum_{\ell=1}^{M}\big|\underline{\underline{\widehat{\mathcal{J}}}}^{(\ell)}\big| = (-1)^M(2M+1)!\{|\underline{\mathcal{J}} - 1/3\underline{\mathcal{J}}^{(2)}| - |\underline{\mathcal{J}} - 1/3\underline{\mathcal{J}}^{(2)} + \underline{\mathcal{J}}^{(1)}|\}. \tag{E45}$$

Lastly, employing the property

$$|\underline{\boldsymbol{A}} - \boldsymbol{a}\boldsymbol{b}^{\mathsf{T}}| - |\underline{\boldsymbol{A}} - \boldsymbol{c}\boldsymbol{b}^{\mathsf{T}}| = |\underline{\boldsymbol{A}} - (\boldsymbol{a}-\boldsymbol{c})\boldsymbol{b}^{\mathsf{T}}| - |\underline{\boldsymbol{A}}| \tag{E46}$$

with $\underline{\boldsymbol{A}} \to \underline{\mathcal{J}}$, $3\boldsymbol{a}$ with Eq. (E39), $3\boldsymbol{b}$ with Eq. (E40), and $3\boldsymbol{c}$ with Eq. (E42), we can simplify Eq. (E45) to

$$\frac{1}{3}\frac{(2M+1)!}{2^M}\sum_{\ell=1}^{M}\big|\underline{\underline{\widehat{\mathcal{J}}}}^{(\ell)}\big| = (-1)^M(2M+1)!\{|\underline{\mathcal{J}} - \underline{\mathcal{J}}^{(1)}| - |\underline{\mathcal{J}}|\}. \tag{E47}$$

Putting it all together gives the final mean first-passage time with M paired defects

$$\mathfrak{T}_{\boldsymbol{n}_0\to\boldsymbol{n}} = \frac{(\mathcal{F}_{\boldsymbol{n}_0\to\boldsymbol{n}} - 1)|\underline{\mathcal{J}}| + |\underline{\mathcal{J}} - \underline{\mathcal{J}}^{(1)}|}{|\underline{\mathcal{J}} - \underline{\mathcal{J}}^{(2)}|}. \tag{E48}$$

If we divide through all the terms by $\prod_{(\boldsymbol{u},\boldsymbol{v})\in S}\mathcal{R}_{\boldsymbol{u}}\mathcal{R}_{\boldsymbol{v}}$, we obtain the mean in Eq. (8), also reported in Eq. (A2).

2. Mean first passage in the presence of sticky or slippery sites

To build the first-passage probability with sticky and slippery heterogeneities (see Appendix D 1 and Fig. 17) we use the propagator given in Eq. (5), where the matrices $\underline{\boldsymbol{H}}$ and $\underline{\boldsymbol{H}}(\boldsymbol{n},\boldsymbol{n}_0)$ are given, respectively, by Eqs. (D20) and (D21). As the procedure is similar to the one used to derive the general MFPT given in Eq. (8), we outline only the key steps.

Starting from

$$\widetilde{\mathbb{F}}_{\boldsymbol{n}_0}(\boldsymbol{n},z) = \frac{\widetilde{\Phi}_{\boldsymbol{n}_0}(\boldsymbol{n},z)}{\widetilde{\Phi}_{\boldsymbol{n}}(\boldsymbol{n},z)} = \frac{(\widetilde{\varphi}_{\boldsymbol{n}_0}(\boldsymbol{n},z) - 1)|\underline{\boldsymbol{H}}| + |\underline{\boldsymbol{H}} - \underline{\boldsymbol{G}}(\boldsymbol{n},\boldsymbol{n}_0)|}{(\widetilde{\varphi}_{\boldsymbol{n}}(\boldsymbol{n},z) - 1)|\underline{\boldsymbol{H}}| + |\underline{\boldsymbol{H}} - \underline{\boldsymbol{G}}(\boldsymbol{n},\boldsymbol{n})|}, \tag{E49}$$

where we have called $\underline{\boldsymbol{H}}(\boldsymbol{n},\boldsymbol{n}_0) = \underline{\boldsymbol{H}} - \underline{\boldsymbol{G}}(\boldsymbol{n},\boldsymbol{n}_0)$, with $\underline{\boldsymbol{H}}$ and $\underline{\boldsymbol{H}}(\boldsymbol{n},\boldsymbol{n}_0)$ given, respectively, by Eqs. (D20) and (D21). Since matrix $\underline{\boldsymbol{G}}(\boldsymbol{n},\boldsymbol{m}) = \boldsymbol{a}\boldsymbol{b}^{\mathsf{T}}$, where $\boldsymbol{a}$ and $\boldsymbol{b}$ are column vectors with elements $\boldsymbol{a}_i = \widetilde{\varphi}_{\boldsymbol{m}}(\boldsymbol{w}_i,z)$ and $\boldsymbol{b}_i = \widetilde{Q}_{\boldsymbol{w}_i}(\boldsymbol{n},z)$ with $\widetilde{Q}_{\boldsymbol{w}}(\boldsymbol{n},z)$ given by Eq. (D17). Dividing both the numerator and denominator of Eq. (E49) by $\widetilde{\varphi}_{\boldsymbol{n}_0}(\boldsymbol{n},z)\prod_{i=1}^{M}\widetilde{\varphi}_{\boldsymbol{w}_i}(\boldsymbol{w}_i,z)$ we find

$$\widetilde{\mathbb{F}}_{\boldsymbol{n}_0}(\boldsymbol{n},z) = \frac{\big(\widetilde{F}_{\boldsymbol{n}_0}(\boldsymbol{n},z) - 1\big)|\underline{\boldsymbol{J}}| + |\underline{\boldsymbol{J}}(\boldsymbol{n},\boldsymbol{n}_0)|}{|\underline{\boldsymbol{J}}(\boldsymbol{n},\boldsymbol{n})|}, \tag{E50}$$

where we rewrite the elements of the matrices in terms of first-passage and first-return probabilities giving the definitions

$$\underline{\boldsymbol{J}}_{i,j} = \widetilde{Q}_{\boldsymbol{w}_j}(\boldsymbol{w}_i,z) - z^{-1}\delta_{i,j}[1 - \widetilde{R}(\boldsymbol{w}_i,z)], \tag{E51}$$

$$\underline{\boldsymbol{J}}(\boldsymbol{n},\boldsymbol{n})_{i,j} = \underline{\boldsymbol{J}}_{i,j} - \widetilde{F}_{\boldsymbol{n}}(\boldsymbol{w}_i,z)\widetilde{Q}_{\boldsymbol{w}_j}(\boldsymbol{n},z), \tag{E52}$$

$$\underline{\boldsymbol{J}}(\boldsymbol{n},\boldsymbol{n}_0)_{i,j} = \underline{\boldsymbol{J}}_{i,j} - \widetilde{F}_{\boldsymbol{n}_0}(\boldsymbol{w}_i,z)\widetilde{Q}_{\boldsymbol{w}_j}(\boldsymbol{n},z), \tag{E53}$$

with

$$\widetilde{Q}_{\boldsymbol{w}_j}(\boldsymbol{n}, z) = \sum_{s=1}^{d} \lambda_{\boldsymbol{w}_j^{(rs)}, \boldsymbol{w}_j} \widetilde{F}_{\langle \boldsymbol{w}_j - \boldsymbol{w}_j^{(rs)} \rangle}(\boldsymbol{n}, z) + \lambda_{\boldsymbol{w}_j^{(ls)}, \boldsymbol{w}_j} \widetilde{F}_{\langle \boldsymbol{w}_j - \boldsymbol{w}_j^{(ls)} \rangle}(\boldsymbol{n}, z). \tag{E54}$$

Note that Eqs. (E6)–(E8) are now different from Eqs. (E51)–(E53).

The derivative with respect to z yields Eq. (E9); however, to evaluate the limit $z \to 1$, one must employ de L'Hôpital's rule $2M$ times on Eq. (E9), where previously the rule was used $4M$ times. This is because the elements of the matrices given by Eqs. (E51)–(E53) need only be differentiated once to give nonzero contributions, i.e., the determinants, $|\underline{\boldsymbol{H}}|$, $|\underline{\boldsymbol{H}}(\boldsymbol{n}, \boldsymbol{n})|$ and $|\underline{\boldsymbol{H}}(\boldsymbol{n}, \boldsymbol{n}_0)|$ must be differentiated M times. Following through one finds, instead Eq. (E55),

$$\mathcal{D}_z \cdot \widetilde{\mathbb{F}}_{\boldsymbol{n}_0}(\boldsymbol{n}, z)\big|_{z=1} = \left.\frac{\mathcal{D}_z^{M+1} \cdot \mathbb{N} - \mathcal{D}_z^{M+1} \cdot \mathbb{D}}{(M+1)\mathcal{D}_z^{M} \cdot \mathbb{D}}\right|_{z \to 1}. \tag{E55}$$

After computing the derivatives explicitly one obtains Eq. (8), but this time

$$\underline{\boldsymbol{\mathcal{H}}}_{i,j} = \frac{1}{\mathcal{R}_{\boldsymbol{w}_i}} \mathcal{Q}_{\boldsymbol{w}_j}(\boldsymbol{w}_i) + \delta_{i,j}, \tag{E56}$$

$$\underline{\boldsymbol{\mathcal{H}}}_{i,j}^{(1)} = \frac{1}{\mathcal{R}_{\boldsymbol{w}_i}} \mathcal{F}_{\langle \boldsymbol{n}_0 - \boldsymbol{n} \rangle \to \boldsymbol{w}_i} \mathcal{Q}_{\boldsymbol{w}_j}(\boldsymbol{n}), \tag{E57}$$

$$\underline{\boldsymbol{\mathcal{H}}}_{i,j}^{(2)} = \frac{1}{\mathcal{R}_{\boldsymbol{w}_i}} \mathcal{Q}_{\boldsymbol{w}_j}(\boldsymbol{n}), \tag{E58}$$

and with

$$\mathcal{Q}_{\boldsymbol{w}_j}(\boldsymbol{n}) = \sum_{s=1}^{d} \lambda_{\boldsymbol{w}_j^{(rs)}, \boldsymbol{w}_j} \mathcal{F}_{\langle \boldsymbol{w}_j - \boldsymbol{w}_j^{(rs)} \rangle \to \boldsymbol{n}} + \lambda_{\boldsymbol{w}_j^{(ls)}, \boldsymbol{w}_j} \mathcal{F}_{\langle \boldsymbol{w}_j - \boldsymbol{w}_j^{(ls)} \rangle \to \boldsymbol{n}}. \tag{E59}$$

3. Mean first-return time

Through the renewal equation we also have the return probability relation

$$\widetilde{\mathbb{R}}(\boldsymbol{n}, z) = 1 - \frac{1}{\widetilde{\Phi}_{\boldsymbol{n}}(\boldsymbol{n}, z)} = \frac{(\widetilde{\varphi}_{\boldsymbol{n}}(\boldsymbol{n}, z) - 2)|\underline{\boldsymbol{H}}| + |\underline{\boldsymbol{H}}(\boldsymbol{n}, \boldsymbol{n})|}{(\widetilde{\varphi}_{\boldsymbol{n}}(\boldsymbol{n}, z) - 1)|\underline{\boldsymbol{H}}| + |\underline{\boldsymbol{H}}(\boldsymbol{n}, \boldsymbol{n})|}. \tag{E60}$$

Dividing both the numerator and denominator of Eq. (E60) by $\widetilde{\varphi}_{\boldsymbol{n}}(\boldsymbol{n}, z) \prod_{i=1}^{M} \widetilde{\varphi}_{\boldsymbol{w}_i}(\boldsymbol{w}_i, z)$ and simplifying gives

$$\widetilde{\mathbb{R}}(\boldsymbol{n}, z) = \frac{(\widetilde{R}(\boldsymbol{n}, z) - 1)|\underline{\boldsymbol{J}}| + |\underline{\boldsymbol{J}}(\boldsymbol{n}, \boldsymbol{n})|}{|\underline{\boldsymbol{J}}(\boldsymbol{n}, \boldsymbol{n})|}, \tag{E61}$$

where $\underline{\boldsymbol{J}}$, $\underline{\boldsymbol{J}}(\boldsymbol{n}, \boldsymbol{n}_0)$ and $\underline{\boldsymbol{J}}(\boldsymbol{n}, \boldsymbol{n})$ are given by Eqs. (E6), (E7), and (E8) in the case of the general paired defect. Whereas for the sticky-slippery defects one would use Eqs. (E51), (E53), and (E52). In either case Eq. (E61) is structurally identical to Eq. (E5) and one employs the same procedure as the one employed to derive the MFPT to obtain, also given in Eq. (9),

$$\mathfrak{R}_{\boldsymbol{n}} = \frac{\mathcal{R}_{\boldsymbol{n}}|\underline{\boldsymbol{\mathcal{H}}}|}{|\underline{\boldsymbol{\mathcal{H}}} - \underline{\boldsymbol{\mathcal{H}}}^{(2)}|} \tag{E62}$$

where $\underline{\boldsymbol{\mathcal{H}}}$ and $\underline{\boldsymbol{\mathcal{H}}}^{(2)}$ are given, respectively, by Eqs. (A3) and (A5) in the case of paired defects, while for sticky-slippery defects one would use the definitions in Eqs. (E56) and (E58).

APPENDIX F: STEADY-STATE PROBABILITY

Using the final value theorem, the steady-state probability is given by

$$(1-z)\widetilde{\Phi}_{\boldsymbol{n}_0}(\boldsymbol{n}, z)|_{z\to 1} = (1-z)\widetilde{\varphi}_{\boldsymbol{n}_0}(\boldsymbol{n}, z)|_{z\to 1} - \left.\frac{(1-z)|\underline{\boldsymbol{H}}| - (1-z)|\underline{\boldsymbol{H}} - \underline{\boldsymbol{G}}(\boldsymbol{n}, \boldsymbol{n}_0)|}{|\underline{\boldsymbol{H}}|}\right|_{z\to 1}, \tag{F1}$$

taking the limit requires a similar procedure as the one given for the derivation of the MFPT see Appendix E, and after some algebra one finds

$$(1-z)\widetilde{\Phi}_{\boldsymbol{n}_0}(\boldsymbol{n}, z)|_{z\to 1} = \frac{1}{\mathcal{R}_{\boldsymbol{n}}} \frac{|\underline{\boldsymbol{\mathcal{H}}} - \underline{\boldsymbol{\mathcal{H}}}^{(2)}|}{|\underline{\boldsymbol{\mathcal{H}}}|}, \tag{F2}$$

which (as expected) is the reciprocal of the MRT and where the elements of $\boldsymbol{\mathcal{H}}$, $\boldsymbol{\mathcal{H}}^{(1)}$, and $\boldsymbol{\mathcal{H}}^{(2)}$ are defined, respectively, in Eqs. (A3), (A4), and (A5) for the general case, and Eqs. (E56), (E57), and (E58) when the heterogeneities are only sticky and slippery.

APPENDIX G: FIRST-PASSAGE STATISTICS IN ONE-DIMENSIONAL DOMAINS

Here we display some explicit miscellaneous expressions for 1D systems that we have omitted from the main text.

1. Mean first-passage time in periodic domains

In a periodic domain when $n \leqslant u < n_0$ or $n > u \geqslant n_0$ the MFPT can be shown to be equal to

$$\mathfrak{F}_{n_0\to n} = \mathcal{F}_{n_0\to n} + \frac{\lambda(n-n_0-\text{sign}(n-n_0))[N\,\text{sign}(n-n_0)+1-2(n-u)]}{p\big[N\frac{p}{2}+\lambda(1-N)\big]}, \tag{G1}$$

while when both n and n_0 are to the right or left of the barrier ones finds

$$\mathfrak{F}_{n_0\to n} = \mathcal{F}_{n_0\to n} + \frac{\lambda(n-n_0)[N\,\text{sign}(u-n)+1-2(n-u)]}{p\big[N\frac{p}{2}+\lambda(1-N)\big]}, \tag{G2}$$

where $\text{sign}(m)=1$ for $m \geqslant 0$ and $\text{sign}(m)=-1$ for $m<0$ is the discrete signum function.

2. Mean return time in 1D

Using Eq. (9) one can show that the mean return time in 1D with a single barrier at u with periodic boundary conditions is given by

$$\mathfrak{R}_n = \begin{cases} \frac{\frac{q}{2}[N^2-(N^2-N)(\lambda_u+\lambda_v)]}{N(q/2-\lambda_v)+\lambda_u(n-u)-\lambda_v(n-u-1)}, & n \leqslant u, \\ \frac{\frac{q}{2}[N^2-(N^2-N)(\lambda_u+\lambda_v)]}{N(q/2-\lambda_u)+\lambda_u(n-u)-\lambda_v(n-u-1)}, & n \geqslant u+1. \end{cases} \tag{G3}$$

When the barrier is impenetrable in both directions, i.e., $\lambda_{u+1,u}, \lambda_{u,u+1} \to \frac{q}{2}$, the mean return time with periodic boundary condition remains $\mathfrak{R}_n = N$ as we have transformed the periodic boundary condition to a reflecting one. However, to recover the analog with reflecting boundary condition requires careful consideration of each of the terms in

$$\mathfrak{R}_n^{(r)} = \begin{cases} N\Big[\frac{q/2-\lambda_{u+1,u}}{q/2-\lambda_{u,u+1}}\Big] - u\Big[\frac{\lambda_{u,u+1}-\lambda_{u+1,u}}{q/2-\lambda_{u,u+1}}\Big] & n \leqslant u \\ N - u\Big[\frac{\lambda_{u,u+1}-\lambda_{u+1,u}}{q/2-\lambda_{u+1,u}}\Big] & n \geqslant u+1 \end{cases}. \tag{G4}$$

When $n \leqslant u$, by expanding the first term in Eq. (G4) it is obvious to see that it gives no contribution to $\mathfrak{R}_n^{(r)}$,

$$N \lim_{\substack{\lambda_{u+1,u}\to\frac{q}{2}\\ \lambda_{u,u+1}\to\frac{q}{2}}} \left(\frac{\frac{q}{2}-\lambda_{u+1,u}}{\frac{q}{2}-\lambda_{u,u+1}}\right) = \frac{Nq}{2} \lim_{\lambda_{u,u+1}\to\frac{q}{2}} \left(\frac{1}{\frac{q}{2}-\lambda_{u,u+1}}\right) - \frac{Nq}{2} \lim_{\lambda_{u,u+1}\to\frac{q}{2}} \left(\frac{1}{\frac{q}{2}-\lambda_{u,u+1}}\right) = 0, \tag{G5}$$

while the second term gives

$$u \lim_{\substack{\lambda_{u+1,u}\to\frac{q}{2}\\ \lambda_{u,u+1}\to\frac{q}{2}}} \left(\frac{\lambda_{u,u+1}-\lambda_{u+1,u}}{\frac{q}{2}-\lambda_{u,u+1}}\right) = \frac{uq}{2} \lim_{\lambda_{u,u+1}\to\frac{q}{2}} \left(\frac{1}{\frac{q}{2}-\lambda_{u,u+1}}\right) - u \lim_{\lambda_{u,u+1}\to\frac{q}{2}} \left(\frac{\lambda_{u,u+1}}{\frac{q}{2}-\lambda_{u,u+1}}\right) = u, \tag{G6}$$

similar argument can be made for the case when $n > u$ to give $\mathfrak{R}_n^{(r)} = N - u$.

3. Mean exit time

In 1D, one find simple expressions for the mean exit times, using known expressions for the defect free exit time $\mathcal{E}_{n_0} = (N-n_0)(n_0-1)/q$ [68], and the overall survival probability of the 1D diffusive propagator with absorbing boundaries, given by

$$\lim_{z\to 1} \widetilde{\varphi}_{n_0}(n,z) = \frac{1}{q}\frac{2(N-n_>)(n_<-1)}{N-1}, \tag{G7}$$

where $n_> = \frac{1}{2}[|n-n_0|+(n+n_0)]$ and $n_< = \frac{1}{2}[|n-n_0|-(n+n_0)]$. When $n_0 \geqslant u+1$, we find the simple relatively relation

$$\mathfrak{E}_{n_0} = \mathcal{E}_{n_0} + \frac{(2u-N)(N-n_0)[u(\lambda_u-\lambda_v)-\lambda_u]}{q\big[N\big(\frac{q}{2}-\lambda_v\big)+\lambda_u+\lambda_v-u(\lambda_u-\lambda_v)-\frac{q}{2}\big]}, \tag{G8}$$

whereas $n_0 \leqslant u$ yields

$$\mathfrak{E}_{n_0} = \mathcal{E}_{n_0} + \frac{(2u-N)(1-n_0)[(u-N)(\lambda_u-\lambda_v)-\lambda_v]}{q\big[N\big(\frac{q}{2}-\lambda_v\big)+\lambda_u+\lambda_v-u(\lambda_u-\lambda_v)-\frac{q}{2}\big]}. \tag{G9}$$

APPENDIX H: EFFICIENT EVALUATION OF THE PROPAGATOR IN FINITE DOMAINS: THE BLOCK IN THE MATRIX CONSTRUCTION

When the number of paired defects is sufficiently small, e.g., $M \lessapprox 10$ it is convenient to compute the elements of the matrices in Eqs. (5) and (8) directly using, respectively, the homogeneous propagators and mean first-passage times. Whereas, for larger values of M it is more efficient to evaluate the heterogeneous propagator using a block matrix construction containing eigenvectors and eigenvalues of the transition matrix. In what follows we describe the procedure for a 1D system while the extension to higher dimension will be addressed in the following subsection. We define the matrices containing the right eigenvectors as

$$\underline{\boldsymbol{R}}_{i,k} = g^{(\gamma,r)}(u_i, k) \quad \text{and} \quad \underline{\boldsymbol{R}}'_{i,k} = g^{(\gamma,r)}(v_i, k), \tag{H1}$$

where $g^{(\gamma,r)}(u_i, k)$ and $g^{(\gamma,r)}(v_i, k)$ are, respectively the u_ith and v_ith component of the kth right eigenvector given by Eq. (H16), with the type of boundary condition described by γ. Similarly for the matrices containing the left eigenvectors we define

$$\underline{\boldsymbol{L}}_{k,i} = g^{(\gamma,\ell)}(u_i, k) \quad \text{and} \quad \underline{\boldsymbol{L}}'_{k,i} = g^{(\gamma,\ell)}(v_i, k), \tag{H2}$$

where $g^{(\gamma,\ell)}(u_i, k)$ and $g^{(\gamma,\ell)}(v_i, k)$ are, respectively, the u_ith and v_ith component of the kth right eigenvector given by Eq. (H16), while for the matrices of eigenvalues we define a diagonal matrix $\underline{\boldsymbol{K}}$ with elements $\underline{\boldsymbol{K}}_{k,k} = s^{(\gamma)}(k)$ where $s^{(\gamma)}(k)$ is the kth eigenvalue of the homogeneous system given by, e.g., Eq. (4) of Ref. [68] or Eq. (22) of Ref. [69]. For the dependence on the occupation site n and the initial site n_0 we define the row vector $\boldsymbol{r}_n$ and the column $\boldsymbol{\ell}_{n_0}$ containing, respectively, the nth and n_0th components of the right and left eigenvectors. Finally we define the diagonal matrices

$$\underline{\boldsymbol{\Lambda}}_{i,i} = \lambda_{u_i,v_i} \quad \text{and} \quad \underline{\boldsymbol{\Lambda}}'_{i,i} = \lambda_{v_i,u_i}, \tag{H3}$$

which contain all the heterogeneous parameter values. The size of these matrices depend on the number of heterogeneities, M, the size of the domain N or both: the matrices $\underline{\boldsymbol{R}}$ and $\underline{\boldsymbol{R}}'$ are of size $M \times N$; the matrices $\underline{\boldsymbol{L}}$ and $\underline{\boldsymbol{L}}'$ are of size $N \times M$; the matrices $\underline{\boldsymbol{\Lambda}}$ and $\underline{\boldsymbol{\Lambda}}'$ are of size $M \times M$; and lastly $\underline{\boldsymbol{K}}$ is of size $N \times N$. From these definitions it follows that

$$\underline{\boldsymbol{H}} = \underline{\boldsymbol{Y}} - z^{-1}\underline{\boldsymbol{I}} \quad \text{and} \quad \underline{\boldsymbol{G}}(n, n_0) = \boldsymbol{os} \tag{H4}$$

where

$$\underline{\boldsymbol{Y}} = [\underline{\boldsymbol{\Lambda}}\underline{\boldsymbol{R}} - \underline{\boldsymbol{\Lambda}}'\underline{\boldsymbol{R}}'][\underline{\boldsymbol{I}} - z\underline{\boldsymbol{K}}]^{-1}[\underline{\boldsymbol{L}} - \underline{\boldsymbol{L}}'] \tag{H5}$$

$$\boldsymbol{o} = [\underline{\boldsymbol{\Lambda}}\underline{\boldsymbol{R}} - \underline{\boldsymbol{\Lambda}}'\underline{\boldsymbol{R}}'][\underline{\boldsymbol{I}} - z\underline{\boldsymbol{K}}]^{-1}\boldsymbol{\ell}_{n_0} \tag{H6}$$

$$\boldsymbol{s} = \boldsymbol{r}_n[\underline{\boldsymbol{I}} - z\underline{\boldsymbol{K}}]^{-1}[\underline{\boldsymbol{L}} - \underline{\boldsymbol{L}}'], \tag{H7}$$

By defining the block matrices

$$\underline{\boldsymbol{X}}_L = \begin{pmatrix} \underline{\boldsymbol{L}} - \underline{\boldsymbol{L}}' & \boldsymbol{\ell}_{n_0} \end{pmatrix} \quad \text{and} \quad \underline{\boldsymbol{X}}_R = \begin{pmatrix} \boldsymbol{u}\underline{\boldsymbol{R}} - \boldsymbol{u}'\underline{\boldsymbol{R}}' \\ \boldsymbol{r}_n \end{pmatrix} \tag{H8}$$

we find

$$\underline{\boldsymbol{X}}_R[\underline{\boldsymbol{I}} - z\underline{\boldsymbol{K}}]^{-1}\underline{\boldsymbol{X}}_L = \begin{pmatrix} \underline{\boldsymbol{Y}} & \boldsymbol{o} \\ \boldsymbol{s}^{\mathsf{T}} & \widetilde{\varphi}_{n_0}(n, z) \end{pmatrix}. \tag{H9}$$

A similar approach can be used for the matrices involved in the MFPT given by Eqs. (A3), (A4), and (A5).

1. The block matrix construction in higher dimensions

In higher dimension the transition dynamics of a lattice random walk are described by a tensor and extending the block matrix construction hinges on "flattening" the vector coordinates to a scalar. There are many methods one can employ to achieve this and we outline a suitable one below. Given the site $\boldsymbol{n} = (n_1, \dots, n_d)$ in a d-dimensional lattice of size $\boldsymbol{N} = N_1, \dots N_d$, we define

$$\hat{n} = 1 + \sum_{i=1}^{d} \left(\prod_{j=1}^{i-1} N_j \right) (n_i - 1) \tag{H10}$$

and

$$\hat{k} = 1 + \sum_{i=1}^{d} \left(\prod_{j=1}^{i-1} N_j \right) (k_i - 1), \tag{H11}$$

where $\hat{n}$ represents the "flattened" site while $\hat{k}$ is the "flattened" eigen-index. Using these indices we define

$$\underline{\boldsymbol{R}}_{i,\hat{k}} = \prod_{j=1}^{d} g_r^{(\gamma_j)}(u_{i_j}, k_j) \quad \text{and} \quad \underline{\boldsymbol{R}}'_{i,\hat{k}} = \prod_{j=1}^{d} g_r^{(\gamma_j)}(v_{i_j}, k_j), \tag{H12}$$

where the products ares over jth component of the sites, e.g., $\boldsymbol{u}_i = (u_{i_1}, \dots, u_{i_d})$. Similarly, the matrices containing the left eigenvectors are defined as

$$\underline{\boldsymbol{L}}_{\hat{k},i} = \prod_{j=1}^{d} g_\ell^{(\gamma_j)}(u_{i_j}, k_j) \quad \text{and} \quad \underline{\boldsymbol{L}}'_{\hat{k},i} = \prod_{j=1}^{d} g_\ell^{(\gamma_j)}(v_{i_j}, k_j), \tag{H13}$$

leaving the matrix of eigenvalues defined as $\underline{\boldsymbol{K}}_{\hat{k},\hat{k}} = \frac{1}{d}\sum_{j=1}^{d} s^{(\gamma)}(k_j)$. The ith element of the vectors containing the dependence on $\boldsymbol{n}$, and $\boldsymbol{n}_0$ is given, respectively, by $\boldsymbol{r}_{\boldsymbol{n}_i} = \prod_{j=1}^{d} g_r^{(\gamma_j)}(n_{i_j}, k_j)$ and $\boldsymbol{\ell}_{\boldsymbol{n}_{0i}} = \prod_{j=1}^{d} g_\ell^{(\gamma_j)}(n_{0_{i_j}}, k_j)$. The remainder of the procedure is identical to the 1D case outlined previously.

2. Eigenvectors and eigenvalues of transition matrix of the one-dimensional lattice random walk

Since the high-dimensional eigenvectors and eigenvalues are composed of the 1D case, repeated here the quantities of interest diffusive case as an example. The Master equation governing a random walk on a finite lattice with N distinct sites ($1 \leqslant n \leqslant N$) is written as

$$\varphi(n, t + t) = \sum_{m=1}^{N} \underline{\boldsymbol{A}}_{n,m}\varphi(m, t), \tag{H14}$$

where $\underline{\boldsymbol{A}}$ is the transition matrix. The matricial form allows one to write easily the propagator as

$$\varphi_{n_0}(n, t) = \sum_{k=1}^{N} g_r^{(\gamma)}(n, k) g_\ell^{(\gamma)}(n_0, k) s^{(k)}(k)^t, \tag{H15}$$

where $g_r^{(\gamma)}(n, k)$ and $g_\ell^{(\gamma)}(n, k)$ are the nth component of, respectively, the kth right and left eigenvectors of the transition matrix and $s^{(\gamma)}(k)$ is the kth eigenvalue. The right eigenvectors

are given by

$$g_r^{(\gamma)}(n,k) = \begin{cases} \frac{a_k}{\sqrt{N}} \cos\left[\left(n-\frac{1}{2}\right)\frac{(k-1)\pi}{N}\right] & \gamma = r, \\ \frac{1}{\sqrt{N}} \exp\left[\frac{2\pi n i(k-1)}{N}\right] & \gamma = p, \\ \sqrt{\frac{2}{N}} \sin\left[\left(\frac{n-1}{N-1}\right)k\pi\right] & \gamma = a, \\ \frac{2}{\sqrt{2N-1}} \cos\left[\left(n-\frac{1}{2}\right)\frac{2k-1}{2N-1}\pi\right] & \gamma = m, \end{cases} \tag{H16}$$

with $a_k = 2$ for $k = 1$ and $a_k = 1$ for all other values of k, while the left eigenvectors $g_\ell^{(\gamma)}(n,k)$ are identical to Eq. (H16) for all cases except the periodic boundary condition $\gamma = p$, where instead it is given by $g_\ell^{(p)}(n,k) = 1/g_r^{(p)}(n,k)$; finally the eigenvalues can be found in Eq. (4) of Ref. [68]. For periodic ($\gamma = p$) and reflecting domains ($\gamma = r$) with N distinct sites we have $k \in [1, N]$ eigenvalues and eigenvectors, for absorbing boundary conditions ($\gamma = a$) with absorbing sites at $n = 1$ and $n = N$ we have $k \in [1, N-2]$ eigenvalues, lastly for mixed boundary condition ($\gamma = m$) with a reflecting end at $n = 1$ and an absorbing one at $n = N$ we have $k \in [1, N-1]$ eigenvalues.
